# Total Synthesis and Biological Evaluation of 22-Hydroxyacuminatine and the Related Natural Products Norketoyobyrine and Naucleficine

**DOI:** 10.3390/molecules30122650

**Published:** 2025-06-19

**Authors:** Shohta Mizuno, Takashi Nishiyama, Hana Bessho, Tetsuya Nakamura, Tomoki Oe, Nanako Hayashi, Yuhzo Hieda, Toshio Motoyashiki, Toshiyuki Hata, Noriyuki Hatae, Tominari Choshi

**Affiliations:** 1 Faculty of Pharmacy and Pharmaceutical Sciences, Fukuyama University, 1 Sanzo, Gakuen-cho, Fukuyama 729-0292, Hiroshima, Japan; s.mizuno@fukuyama-u.ac.jp (S.M.); t_nishiyama@fukuyama-u.ac.jp (T.N.); t-nakamura@fukuyama-u.ac.jp (T.N.); hieda@fukuyama-u.ac.jp (Y.H.); motoyashiki@fukuyama-u.ac.jp (T.M.); hata@fukuyama-u.ac.jp (T.H.); 2 Faculty of Pharmaceutical Sciences, Yokohama University of Pharmacy, 601 Matano, Totsuka-ku, Yokohama 245-0066, Kanagawa, Japan

**Keywords:** total synthesis, 22-hydroxyacuminatine, norketoyobyrine, naucleficine, antiproliferative activity

## Abstract

Aromathecin compounds—which contain the same indolizine core structure as camptothecin-like compounds—are expected to show anticancer activity. Among them, 22-hydroxyacuminatine—which has a substituent on the E-ring of the pentacyclic scaffold—exhibits topoisomerase 1 inhibitory activity; therefore, the development of efficient methods for its synthesis has been actively pursued. Herein, we report a versatile synthetic methodology for introducing various substituents on the E-ring, leading to the total synthesis of 22-hydroxyacuminatine as a model compound of the aromathecin family. The synthesis comprises the following key steps: the synthesis of an isoquinoline *N*-oxide via the thermal cyclization of 2-alkynylbenzaldehyde oxime, the subsequent Reissert–Henze-type reaction to yield an isoquinolone, and the construction of the indolizine moiety (CD-ring) through C–N bond formation via the Mitsunobu reaction. Consequently, a pentacyclic benz[6,7]indolizino[1,2-*b*]quinolin-11(13*H*)-one framework is obtained. Using this methodology, the total synthesis of the natural products norketoyobyrine and naucleficine and an intermediate of the latter, which are indoloquinolizidine-type alkaloids, was achieved, and their antiproliferative activity against HCT-116 human colon cancer cells and HepG2 human liver cancer cells was assessed. Naucleficine and its intermediate exhibited moderate antiproliferative activity against HCT-116 cells, with IC_50_ values of 55.58 and 41.40 μM, respectively.

## 1. Introduction

**Figure 1:**
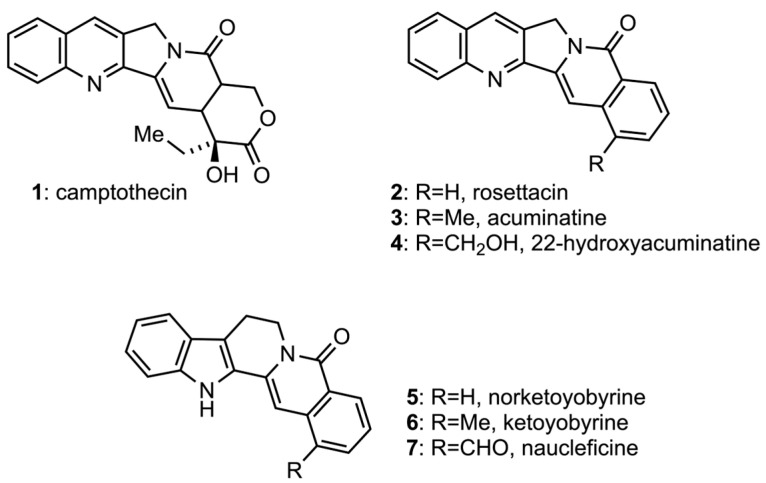
Structures of natural products containing the indolizinone or quinolizinone scaffold.

Camptothecin (CPT: **1**) was isolated from the Chinese tree *Camptotheca acuminata* by Wani et al. in 1966 and reportedly had topoisomerase I (Top1) inhibitory activity (Figure 1) [1]. Subsequently, **1** served as a lead compound for the development of pharmaceuticals, including irinotecan, which are now clinically used. Meanwhile, rosettacin (**2**), in which the lactone E-ring moiety of **1** is replaced by a benzene ring, was synthesized while developing the synthesis method for **1** [2]. Later, in 1992, 22-hydroxyacuminatine (**4**), having the skeleton of **2**, was isolated along with **1** from the seeds of *C. acuminata* [3]. Compound **4** has reportedly displayed weak Top1 inhibitory activity. Compounds **2** and **4**, as well as acuminatine (**3**), are the three members of the aromathecin family targeted for intense synthetic research [4,5]. In particular, the total synthesis of **4** has been reported by six research groups [6,7,8,9,10,11]. These derivatives and other compounds containing the indolizine scaffold have been the subject of drug discovery studies.

Norketoyobyrine (**5**), ketoyobyrine (**6**), and naucleficine (**7**) have a pentacyclic structure similar to that of aromathecins and belong to the indoloquinolizidine alkaloid family. They have been isolated from the *Nauclea latifolia* plant and used in folk medicine (Figure 1) [12]. These products have attracted considerable interest in synthesis owing to their structural features. Various synthetic approaches have been developed for norketoyobyrine (**5**) [11,13,14,15,16,17,18,19,20,21,22,23,24,25] and ketoyobyrine (**6**) [13,26,27]—a degradation product of yohimbine. Naucleficine (**7**) is isolated from the stems of *Nauclea officinalis* (Pierre ex Pitard) and has been used as an anti-inflammatory and antibacterial agent in Chinese folk medicine [28]. The total synthesis of **7** has been reported by three research groups so far [11,29,30]. These products **5**–**7** can be considered as derivatives of aromathecin that underwent a skeletal transformation of its B- and C-rings to conduct structure–activity relationship (SAR) studies. To this aim, performing the total synthesis of these compounds would be highly beneficial.

Our research group has been interested in the unique structure and pharmacological action of condensed heteroaromatic compounds and has been searching for highly active compounds based on these naturally occurring compounds and their derivatives. To date, we have achieved the total synthesis of β-carboline alkaloids (*R*)-(–)-pyridindolols [31,32] and 8-oxoprotoberberine alkaloids alangiumkaloids A and B [33] using a series of key steps, namely, the thermal cyclization of 2-alkynylaryl aldoxime to afford fused pyridine *N*-oxide, followed by the synthesis of fused pyridone via a Reissert–Henze-type reaction.

**Scheme 1:**
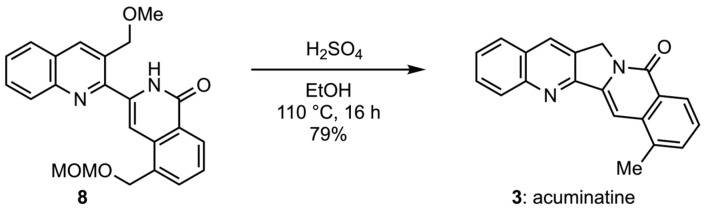
Synthesis of acuminatine by constructing a C-ring.

Furthermore, we have previously reported a synthetic route to **2** by applying a similar series of reactions as the key steps [34]. Herein, we describe the application of this method to the synthesis of **4** (Scheme 1). During the final step for constructing the pentacyclic scaffold by forming the C-ring, isoquinolone **8** was treated with H_2_SO_4_ in EtOH at 110 °C. As expected, a C-ring was formed; however, the 7-methoxymethoxymethyl group was converted to a methyl group, and **3** was obtained in 79% yield. This result, whose reason remains unclear, indicates that this methodology might not be applicable to the synthesis of derivatives with certain substituents on the E-ring.

Therefore, a versatile strategy is still required to synthesize the natural product 22-hydroxyacuminatine (**4**) and its derivatives bearing substituents on the E-ring. In this study, we improved the previously reported synthetic method and applied it to the synthesis of indoloquinolizidine-type alkaloids.

## 2. Results and Discussion

**Scheme 2:**
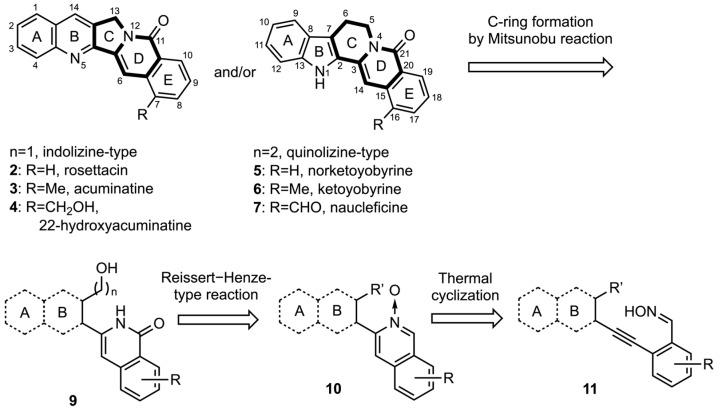
Retrosynthetic analysis of indolizine-type and quinolizine-type pentacyclic scaffolds.

As a starting point of our investigation, we considered intramolecular cyclization under mild conditions to form a C-ring. The retrosynthetic analysis of the pentacyclic scaffolds of indolizine-type **2**–**4** and quinolizine-type **5**–**7** is shown in Scheme 2. The construction of the indolizine and quinolizine scaffolds would be achieved via a C-ring formation in the final stage. To this aim, we considered the Mitsunobu reaction between the alcohol moiety and the amide nitrogen of isoquinolone **9** to form a C–N bond. Precursor **9** would be synthesized via the Reissert–Henze-type reaction of isoquinoline *N*-oxide **10**, formed by the thermal cyclization of 2-alkynylbenzaldehyde oxime **11**. Therefore, we investigated the optimal substrate for converting **10** to **9**.

**Scheme 3:**
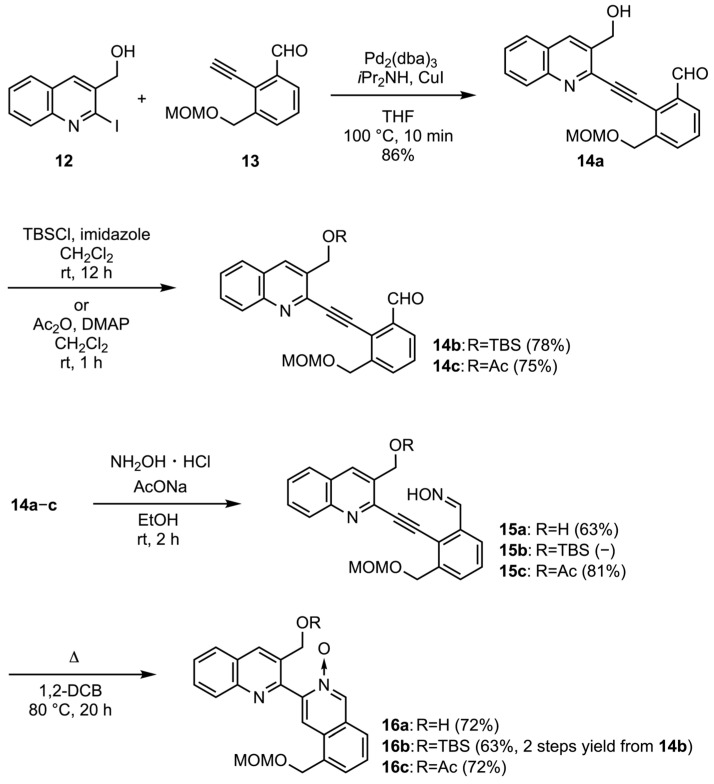
Synthesis of isoquinoline *N*-oxides **16a**–**c**.

As shown in Scheme 3, 2-arylethynylbenzaldehyde **14a** was obtained in 86% yield via the Sonogashira reaction of 3-hydroxymethyl-2-iodoquinoline (**12**) [34] and 2-ethynylbenzaldehyde **13** [33] in the presence of CuI, *i*Pr_2_NH and Pd_2_(dba)_3_. To investigate the optimal synthetic route, *tert*-butyldimethylsilyl (TBS) and acetyl (Ac) groups were selected as protecting groups for the hydroxyl group of **14a**. Compounds **14b**,**c** were obtained by introducing each protecting group to **14a** under general conditions. Subsequently, the three 2-arylethynylbenzaldehydes **14a**–**c** were treated with NH_2_OH to obtain oximes **15a**–**c**, followed by heating in 1,2-dichlorobenzene (DCB) at 80 °C to obtain *N*-oxides **16a**–**c** of the key precursor in moderate yield. Oxime **15b** was unstable and thus was subjected to a thermal cyclization without purification to obtain *N*-oxide **16b** in 63% yield over two steps.

Subsequently, we investigated the conversion of *N*-oxides **16a**–**c** to the desired isoquinolones **17a**–**c** in Ac_2_O at 80 °C with or without microwave (MW) irradiation. The Reissert–Henze-type reaction of *N*-oxides **16** produced 4-hydroxyisoquinolines **18** along with the desired isoquinolones **17**. The product ratios are summarized in Table 1.

Initially, when unprotected *N*-oxide **16a** was heated in Ac_2_O at 80 °C, many products were observed on thin-layer chromatography (TLC). Among them, isoquinolone **17a** and 4-OH **18a** were not obtained, and only small amounts of **17c** and **18c**, which are acetylated hydroxymethyl groups of **17a** and **18a**, were isolated (Table 1, entries 1 and 2). Substrate **16a** did not afford isoquinolone **17c** in the absence or presence of MW irradiation.

Moreover, when *N*-oxide **16b** containing a TBS group was heated in Ac_2_O at 80 °C, **16b** disappeared as revealed by TLC monitoring, but isoquinolone **17b** was obtained in a low 20% yield (Table 1, entry 3). The yield of **17b** could be improved to 36%, and the reaction time was considerably shortened by reacting **16b** under MW irradiation (Table 1, entry 4). However, the yield was still not satisfactory for proceeding to the next step.

Furthermore, we investigated the conversion of *O*-Ac-*N*-oxide **16c** to isoquinolone **17c**. When heated at 80 °C, **17c** and **18c** were obtained in 20% and 27% yields, respectively (Table 1, entry 5). The product ratio of **17c** improved when heating under MW irradiation, affording a yield of 50% (Table 1, entry 6). Considering these results, the Ac group is the most suitable protecting group for the hydroxymethyl group in *N*-oxide **16**.

**Scheme 4:**
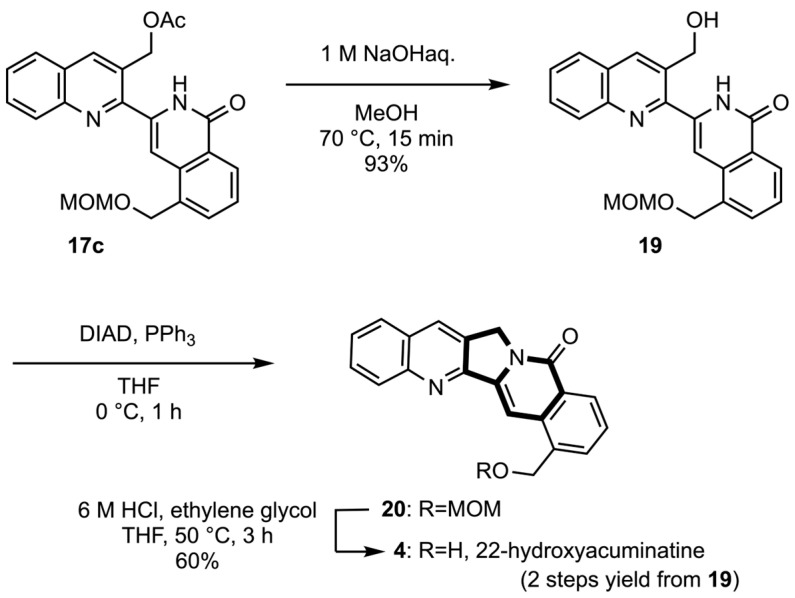
Synthesis of 22-hydroxyacuminatine (**4**).

As a key precursor for the C-ring formation, alcohol **19** was obtained in 93% yield by treating **17c** with 1 M NaOH in MeOH (Scheme 4). Further, **19** was subjected to the Mitsunobu reaction in the presence of diisopropyl azodicarboxylate (DIAD) and PPh_3_, which promoted the C-ring formation to obtain the desired pentacyclic compound **20**. However, **20** was obtained as a mixture with the by-product triphenylphosphine oxide and could not be separated. Therefore, the crude **20** was treated with 6 M HCl and ethylene glycol in THF to deprotect the MOM group [11], affording the desired **4** in 60% yield over two steps. Thus, the total synthesis of 22-hydroxyacuminatine (**4**) as a model compound with a substituent on the E-ring was achieved via an eight-step sequence in 10.5% overall yield. The physical and spectroscopic data of synthesized **4** were consistent with previously reported values [6].

**Scheme 5:**
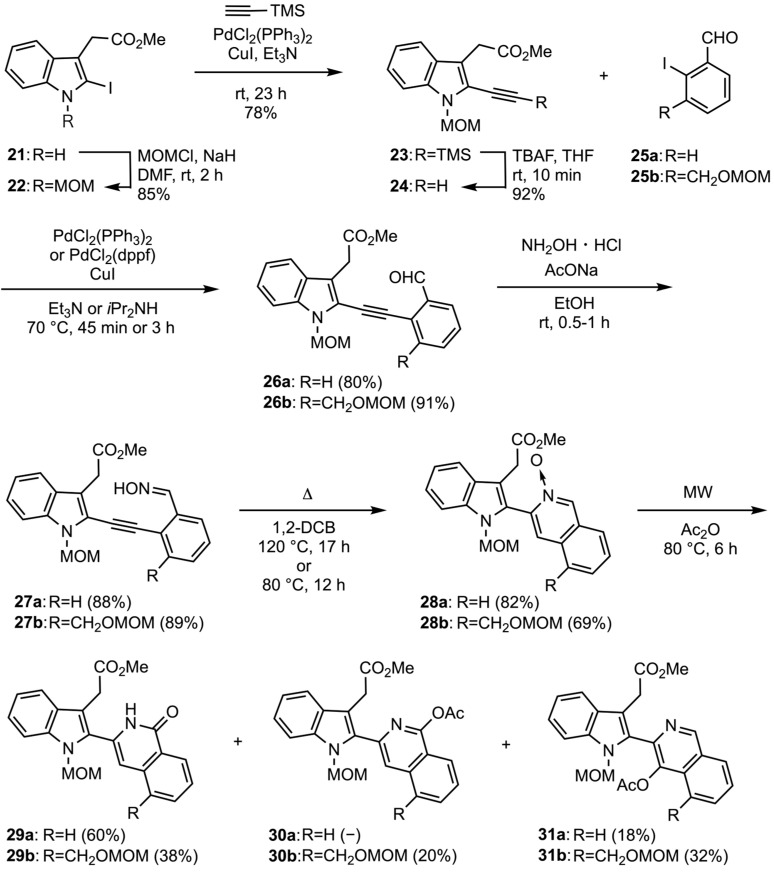
Synthesis of isoquinolones **29a** and **29b**.

Having established an improved synthetic method for the benzo[6,7]indolizino[1,2-*b*]quinoline-11(13*H*)-one scaffold—the core structure of the aromathecin family—and achieved the total synthesis of **4**, we addressed the synthesis of indoloquinolizidine-type alkaloids by applying the same methodology (Scheme 5). Methyl indolylacetate **21** [35] was treated with MOMCl and NaH in DMF to give *N*-MOM-indolylacetate **22** in 85% yield. Subsequently, the Sonogashira coupling of **22** and trimethylsilyl (TMS)-acetylene in the presence of CuI, Et_3_N, and PdCl_2_(PPh_3_)_2_ produced TMS-acetylene **23** in 78% yield. Further, the desilylation of **23** using TBAF provided 2-ethynylindole **24** in 92% yield. The Sonogashira coupling of **24** and 2-iodobenzaldehyde **25a** in the presence of CuI, Et_3_N, and PdCl_2_(PPh_3_)_2_ produced 2-alkynylbenzaldehyde **26a** in 80% yield. The treatment of **26a** with NH_2_OH in EtOH furnished 2-alkynylbenzaldehyde oxime **27a** in 88% yield. Subsequently, heating **27a** in 1,2-DCB at 80 °C to give isoquinoline *N*-oxide **28a** in 82% yield followed by heating **28a** in Ac_2_O at 80 °C under MW irradiation furnished isoquinolone **29a** and 4-OAc **31a** in 60% and 18% yields, respectively.

**Scheme 6:**
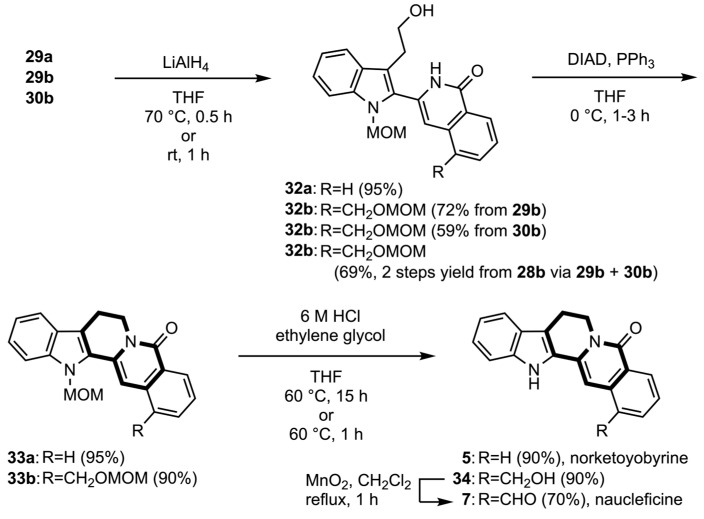
Synthesis of indoloquinolizine-type alkaloids **5** and **7**.

Isoquinoline **29a** was reduced with LiAlH_4_ to give alcohol **32a** in 95% yield. The Mitsunobu reaction of **32a** with DIAD and PPh_3_ formed the C-ring, giving indoloquinolizine **33a** in 95% yield (Scheme 6). Finally, **33a** was treated with 6 M HCl in the presence of ethylene glycol to remove the MOM group, giving **5** in 90% yield. Using the same synthetic route, we also attempted the total synthesis of naucleficine (**7**). In this case, when isoquinolone **29b** was synthesized from *N-*oxide **28b** via the Reissert–Henze-type reaction as shown in Scheme 5, 1-OAc-isoquinoline **30b** was produced in 20% yield along with the desired **29b** (38%) and 4-OAc **31b** (32%). Subsequently, the reduction of **29b** and **30b** with LiAlH_4_ afforded alcohol **32b** in 72% and 59% yields, respectively. Therefore, after obtaining **29b** and **30b** from *N*-oxide **28b**, the mixture was reduced with LiAlH_4_, and alcohol **32b** was afforded as a single product in 69% yield over two steps (Scheme 6). After the Mitsunobu reaction of **32b** to obtain indoloquinolizine **33b** in 90% yield, the two MOM groups of **33b** were removed by treating with 6 M HCl, furnishing alcohol **34** in 90% yield. Finally, **34** was oxidized with MnO_2_ to produce **7** in 70% yield. Thus, we achieved the total synthesis of the indoloquinolizidine-type alkaloids norketoyobyrine (**5**: 10 steps, total yield 17.2%) and naucleficine (**7**: 11 steps, total yield 13.3%).

The physical and spectroscopic data of synthesized **5** and **7** were consistent with the previously reported values [11]. Furthermore, ^1^H NMR, ^13^C NMR, and mass spectroscopy characterization of all the synthesized compounds supported the identified structures (Supplementary Materials).

Furthermore, we compared the total synthesis yields of the three natural products that we synthesized with other methodologies that have already been published, and we summarized the results in Table 2.

**Figure 2:**
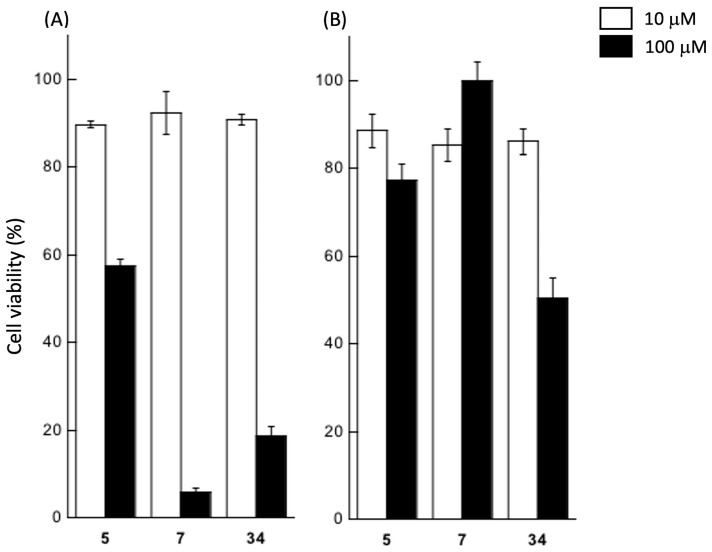
Antiproliferative activities of indoloquinolizine-type natural products **5** and **7** and intermediate **34** against (**A**) HCT-116 and (**B**) HepG2 cells. The open and filled columns correspond to the treatment with compound concentrations of 10 and 100 μM, respectively.

On preliminary work, the indolizine derivatives **2**, **3,** and **4** were indicated weak antiproliferative activity against HCT-116 cells. The synthesized indoloquinolizine-type natural products **5** and **7** and intermediate **34** were assessed for their antiproliferative activity against HCT-116 human colon and HepG2 human liver cancer cells. Figure 2 summarizes the results of the tumor cell viability treatments, which were performed at compound concentrations of 10 and 100 μM for 72 h. Naucleficine (**7**) and intermediate **34** at a concentration of 100 μM showed moderate antiproliferative activities against HCT-116 cells, with IC_50_ values of 55.58 and 41.40 μM, respectively (Table 3). Meanwhile, tested compounds were indicated to have low or almost lost their activity against HepG2 cells.

## 3. Materials and Methods

### 3.1. Chemistry

All non-aqueous reactions were carried out under an atmosphere of nitrogen in dried glassware unless otherwise noted. Solvents were dried and distilled according to standard protocols. Analytical thin-layer chromatography was performed with Silica gel 60PF_254_ (Merck KGaA, Darmstadt, Germany). Silica gel column chromatography was performed with Silica gel 60 (70–230 mesh, Kanto Co. Lit., Tokyo, Japan). All melting points were determined on micro-melting point apparatus MP-500D (Yanaco Technical Science Co., Ltd., Tokyo, Japan) and are uncorrected. Proton nuclear magnetic resonance (^1^H-NMR) spectra were recorded on JEOL JNM-ECZ400S (JEOL Ltd., Tokyo, Japan). Chemical shifts are reported relative to Me_4_Si (δ 0.00). Multiplicity is indicated by one or more of the following: s (singlet); d (doublet); t (triplet); q (quartet); m (multiplet); and br (broad). Carbon nuclear magnetic resonance (^13^C-NMR) spectra were recorded on a JEOL JNM-ECZ400S at 100 MHz. Chemical shifts are reported relative to CDCl_3_ (δ 77.0) and DMSO-*d*_6_ (δ 39.7). Infrared spectra were recorded with ATR method using a Shimadzu FTIR-8000 spectrophotometer (Shimadzu corporation, Kyoto, Japan) and DuraScop (Sensir Technologies, NC, USA). Low and high-resolution mass spectra were recorded on JEOL JMS-700 spectrometers (JEOL Ltd., Tokyo, Japan) by direct inlet system. The microwave assisted reaction was carried out at 180 W and 2450 MHz with Discover (CEM corporation, NC, USA).

#### 3.1.1. 2-(3-Hydroxymethylquinolin-2-yl)ethynyl-3-[(methoxymethoxy)methyl]benzaldehyde (**14a**)

To a solution of 2-iodoquinoline **12** (95 mg, 0.33 mmol), CuI (6 mg, 0.033 mmol), Pd_2_(dba)_3_ (13 mg, 0.013 mmol) and *i*Pr_2_NH (0.3 mL, 2.31 mmol) in THF (1.5 mL), a solution of 2-ethynylbenzaldehyde **13** (81 mg, 0.40 mmol) in THF (1.5 mL) was added. The reaction mixture was stirred at 100 °C for 10 min. After cooling to ambient temperature, the reaction mixture was filtered through Celite pad, washed with EtOAc, and the filtrate was evaporated in vacuo. The residue was purified by column chromatography (EtOAc/hexane 1:3 *v*/*v*) to give the 2-alkynylbenzaldehyde **14a** (124 mg, 86%) as a white solid. mp was 59–61 °C (EtOAc-hexane). IR (ATR) ν = 1685, 2190, and 3629 cm^−1^. ^1^H-NMR (400 MHz, CDCl_3_) δ 3.41 (s, 3H), 3.52 (br s, 1H), 4.79 (s, 2H), 5.00 (s, 2H), 5.06 (s, 2H), 7.54–7.60 (m, 2H), 7.74 (t, *J* = 7.8 Hz, 1H), 7.80 (d, *J* = 7.8 Hz, 1H), 7.84 (d, *J* = 7.8 Hz, 1H), 7.94 (d, *J* = 7.8 Hz, 1H), 8.12 (d, *J* = 7.8 Hz, 1H), 8.25 (s, 1H), and 10.70 (s, 1H). ^13^C-NMR (400 MHz, CDCl_3_) δ 55.6, 62.8, 67.1, 85.6, 95.9, 98.2, 123.7, 127.5 (2C), 127.8, 127.9, 129.1, 129.4, 130.1, 133.8, 135.0, 135.2, 136.8, 141.8, 142.0, 147.6, and 191.5. MS *m*/*z*: 361 (M^+^). HRMS (EI): calcd for C_22_H_19_NO_4_ 361.1314; found 361.1332.

#### 3.1.2. 2-{[3-(*tert*-Butyldimethylsilyloxy)methyl]quinolin-2-yl}ethynyl-3-[(methoxymethoxy)methyl]benzaldehyde (**14b**)

A solution of alcohol **14a** (120 mg, 0.33 mmol) in CH_2_Cl_2_ (2 mL) was dropwise added to a suspension of imidazole (56 mg, 0.82 mmol) in CH_2_Cl_2_ (1 mL) under ice cooling. After stirring at same temperature for 15 min, TBSCl (123 mg, 0.82 mmol) in CH_2_Cl_2_ (1 mL) was added to the reaction mixture and then was stirred at rt for 12 h. After quenching with H_2_O, the reaction mixture was extracted with CH_2_Cl_2_. The organic layer was washed with brine, dried with Na_2_SO_4_, and evaporated in vacuo. The residue was purified by column chromatography (EtOAc/hexane 1:3 *v*/*v*) to give the silyl ether **14b** (123 mg, 78%) as a yellow solid. mp was 74–75 °C (EtOAc-hexane). IR (ATR) ν = 1697, 2168 cm^−1^. ^1^H-NMR (400 MHz, CDCl_3_) δ 0.19 (s, 6H), 1.00 (s, 9H), 3.42 (s, 3H), 4.82 (s, 2H), 5.00 (s, 2H), 5.14 (s, 2H), 7.54–7.61 (m, 2H), 7.73 (t, *J* = 7.8 Hz, 1H), 7.84–7.88 (m, 2H), 7.95 (d, *J* = 7.8 Hz, 1H), 8.12 (d, *J* = 7.8 Hz, 1H), 8.33 (s, 1H), and 10.77 (s, 1H). ^13^C-NMR (100 MHz, CDCl_3_) δ -5.3 (2C), 18.4, 25.9 (3C), 55.6, 62.2, 67.0, 85.7, 96.3, 97.9, 123.7, 126.7, 127.6 (2C), 127.7, 129.1, 129.4, 129.6, 132.7, 133.0, 135.9, 136.7, 140.3, 142.0, 147.3, and 191.2. MS *m*/*z*: 475 (M^+^). HRMS (EI): calcd for C_28_H_33_NO_4_Si 475.2179; found 475.2181.

#### 3.1.3. 2-[3-(Acetoxymethyl)quinolin-2-yl]ethynyl-3-[(methoxymethoxy)methyl]benzaldehyde (**14c**)

A solution of alcohol **14a** (30 mg, 0.083 mmol) in CH_2_Cl_2_ (1 mL) was dropwise added to a suspension of DMAP (12 mg, 0.10 mmol) in CH_2_Cl_2_ (1 mL) under ice cooling. After stirring at same temperature for 15 min, Ac_2_O (9 μL, 0.10 mmol) was added to the reaction mixture and then was stirred at rt for 1 h. After quenching with H_2_O, the reaction mixture was extracted with CH_2_Cl_2_. The organic layer was washed with brine, dried with Na_2_SO_4_, and evaporated in vacuo. The residue was purified by column chromatography (EtOAc/hexane 1:3 *v*/*v*) to give the compound **14c** (25 mg, 75%) as a yellow solid. mp was 110–111 °C (EtOAc-hexane). IR (ATR) ν = 1685, 1736, and 2198 cm^−1^. ^1^H-NMR (400 MHz, CDCl_3_) δ 2.17 (s, 3H), 3.43 (s, 3H), 4.84 (s, 2H), 5.01 (s, 2H), 5.53 (s, 2H), 7.57 (t, *J* = 7.8 Hz, 1H), 7.62 (t, *J* = 7.8 Hz, 1H), 7.78 (t, *J* = 7.8 Hz, 1H), 7.85–7.87 (m, 2H), 7.96 (d, *J* = 7.8 Hz, 1H), 8.13 (d, *J* = 7.8 Hz, 1H), 8.27 (s, 1H), and 10.77 (s, 1H). ^13^C-NMR (100 MHz, CDCl_3_) δ 20.9, 55.6, 63.5, 67.0, 85.7, 96.4, 97.9, 123.5, 126.6, 127.2, 127.7, 128.0, 129.3, 129.5, 130.3, 130.5, 132.9, 136.2, 136.8, 142.0, 142.2, 147.9, 170.6, and 191.4. MS *m*/*z*: 403 (M^+^). HRMS (EI): calcd for C_24_H_21_NO_5_ 403.1420; found 403.1411.

#### 3.1.4. 2-(3-Hydroxymethylquinolin-2-yl)ethynyl-3-[(methoxymethoxy)methyl]benzaldehyde oxime (**15a**)

A mixture of benzaldehyde **14a** (100 mg, 0.25 mmol), NH_2_OH·HCl (34 mg, 0.50 mmol), and AcONa (40 mg, 0.50 mmol) in EtOH (5 mL) was stirred at rt for 2 h. After removal of solvent, the residue was diluted with H_2_O and then filtered off to give the oxime **15a** (65 mg, 63%) as a yellow solid. Mp was 220–221 °C (EtOAc-hexane). IR (ATR) ν = 1736, 2191, 3444, and 3610 cm^−1^. ^1^H-NMR (400 MHz, DMSO-*d*_6_) δ 4.76 (s, 2H), 4.85 (s, 2H), 4.91 (d, *J* = 5.5 Hz, 3H), 5.66 (t, *J* = 5.5 Hz, 1H), 7.52 (t, *J* = 7.8 Hz, 1H), 7.59 (d, *J* = 7.8 Hz, 1H), 7.65 (t, *J* = 7.8 Hz, 1H), 7.78 (t, *J* = 7.8 Hz, 1H), 7.86 (d, *J* = 7.8 Hz, 1H), 8.03–8.06 (m, 2H), 8.44 (s, 1H), 8.68 (s, 1H), and 11.72 (s, 1H). ^13^C-NMR (100 MHz, DMSO-*d*_6_) δ 55.0, 60.3, 67.0, 86.7, 95.8, 96.7, 119.1, 123.9, 127.2, 127.7, 127.9, 128.4, 128.7, 129.7, 129.8, 133.0, 134.8, 136.9, 140.9, 141.2, 146.0, and 146.6. MS *m*/*z*: 376 (M^+^). HRMS (EI): calcd for C_22_H_20_N_2_O_4_ 376.1423; found 376.1441.

#### 3.1.5. 2-[3-(Acetoxymethyl)quinolin-2-yl]ethynyl-3-[(methoxymethoxy)methyl]benzaldehyde oxime (**15c**)

The same procedure as above was carried out with 2-arylethynylbenzaldehyde **14c** (96 mg, 0.23 mmol) to give the oxime **15c** (78 mg, 81%) as a pink solid. mp was 138–139 °C (EtOAc-hexane). IR (ATR) ν = 1751, 2233, and 3629 cm^−1^. ^1^H-NMR (400 MHz, DMSO-*d*_6_) δ 2.12 (s, 3H), 3.29 (s, 3H), 4.74 (s, 2H), 4.84 (s, 2H), 5.47 (s, 2H), 7.53 (t, *J* = 7.8 Hz, 1H), 7.59 (d, *J* = 7.8 Hz, 1H), 7.69 (t, *J* = 7.8 Hz, 1H), 7.82–7.87 (m, 2H), 8.05–8.08 (m, 2H), 8.49 (s, 1H), 8.65 (s, 1H), and 11.72 (s, 1H). ^13^C-NMR (100 MHz, DMSO-*d*_6_) δ 20.7, 55.0, 63.0, 67.0, 86.7, 95.7, 96.6, 118.8, 123.9, 126.8, 128.1 (2C), 128.5, 128.6, 129.9, 130.5, 130.8, 135.0, 136.1, 141.3, 141.8, 145.9, 147.1, and 170.3. MS *m*/*z*: 418 (M^+^). HRMS (EI): calcd for C_24_H_22_N_2_O_5_ 418.1529; found 418.1533.

#### 3.1.6. 3-(3-Hydroxymethylquinolin-2-yl)-5-[(methoxymethoxy)methyl]isoquinoline N-oxide (**16a**)

A solution of oxime **15a** (65 mg, 0.16 mmol) in 1,2-dichlorobenzene (4 mL) was stirred at 80 °C for 20 h. After removal of the solvent, the residue was purified by column chromatography (EtOAc/hexane 1:1 *v*/*v*) to give the *N*-oxide **16a** (43 mg, 72%) as a white solid. mp was 239–240 °C (EtOAc-hexane). IR (ATR) ν = 1230, 3610 cm^−1^. ^1^H-NMR (400 MHz, CDCl_3_) δ 3.36 (s, 3H), 4.57 (s, 2H), 4.71 (s, 2H), 5.02 (s, 2H), 5.61 (br s, 1H), 7.63–7.67 (m, 1H), 7.69 (d, *J* = 7.8 Hz, 1H), 7.74 (d, *J* = 7.8 Hz, 1H), 7.78 (t, *J* = 7.8 Hz, 1H), 7.82 (d, *J* = 7.8 Hz, 1H), 7.95 (d, *J* = 7.8 Hz, 1H), 8.15 (d, *J* = 7.8 Hz, 1H), 8.27 (s, 1H), 8.40 (s, 1H), and 8.99 (s, 1H). ^13^C-NMR (100 MHz, CDCl_3_) δ 55.7, 63.7, 66.2, 95.8, 123.2, 125.5, 127.7, 127.8 (2C), 128.7, 129.4, 129.7, 130.0, 130.1 (2C), 133.8, 134.2, 137.8, 138.1, 146.4, 147.6, and 151.9 MS *m*/*z*: 376 (M^+^). HRMS (EI): calcd for C_22_H_20_N_2_O_4_ 376.1423; found 376.1417.

#### 3.1.7. 2-{3-[(*tert*-Butyldimethylsilyloxy)methyl]quinolin-2-yl}-5-[(methoxymethoxy)methyl]isoquinoline N-oxide (**16b**)

A mixture of benzaldehyde **15b** (43 mg, 0.090 mmol), NH_2_OH·HCl (12 mg, 0.18 mmol), and AcONa (11 mg, 0.14 mmol) in EtOH (3 mL) was stirred at rt for 2 h. The resulting mixture was quenched with water and extracted with EtOAc. The organic layer was washed with water and brine, dried with Na_2_SO_4_, and evaporated in vacuo. Next, 1,2-dichlorobenzene (2 mL) was added to the residue, and the mixture was stirred at 80 °C for 12 h. After removal of the solvent, the residue was purified by column chromatography (EtOAc/hexane 1:1 *v*/*v*) to give the *N*-oxide **16b** (28 mg, 63%) as a black oil. IR (ATR) ν = 1254 cm^−1^. ^1^H-NMR (400 MHz, CDCl_3_) δ –0.07 (s, 3H), 0.02 (s, 3H), 0.83 (s, 9H), 3.36 (s, 3H), 4.70 (s, 2H), 4.77 (d, *J* = 13.7 Hz, 1H), 4.95 (d, *J* = 11.9 Hz, 1H), 5.02 (d, *J* = 11.9 Hz, 1H), 5.11 (d, *J* = 13.7 Hz, 1H), 7.59–7.67 (m, 3H), 7.71–7.76 (m, 2H), 7.93 (d, *J* = 7.8 Hz, 1H), 8.13 (d, *J* = 7.8 Hz, 1H), 8.19 (s, 1H), 8.37 (s, 1H), and 8.90 (s, 1H). ^13^C-NMR (100 MHz, CDCl_3_) δ -5.5 (2C), 18.2, 25.8 (3C), 55.6, 62.2, 66.2, 95.7, 122.6, 124.9, 127.4, 127.6 (2C), 128.3, 129.0, 129.3 (3C), 130.2, 133.9, 134.0, 135.3, 136.5, 146.5, 146.9, and 150.9. MS *m*/*z*: 490 (M^+^). HRMS (EI): calcd for C_28_H_34_N_2_O_4_Si 490.2288; found 490.2276.

#### 3.1.8. 2-(3-Acetoxymethylquinolin-2-yl)-5-[(methoxymethoxy)methyl]isoquinoline N-oxide (**16c**)

The *N*-oxide **16c** (yield 72%) was prepared according to a synthetic method for **16a** as a black solid. mp was 169–171 °C (EtOAc-hexane). IR (ATR) ν = 1230, 1743 cm^−1^. ^1^H-NMR (400 MHz, CDCl_3_) δ 1.94 (s, 3H), 3.37 (s, 3H), 4.71 (s, 2H), 4.97 (d, *J* = 11.9 Hz, 1H), 5.05 (d, *J* = 11.9 Hz, 1H), 5.27 (d, *J* = 13.3 Hz, 1H), 5.45 (d, *J* = 13.3 Hz, 1H), 7.61–7.67 (m, 3H), 7.76 (t, *J* = 7.8 Hz, 2H), 7.93 (d, *J* = 7.8 Hz, 1H), 8.15 (d, *J* = 7.8 Hz, 1H), 8.26 (s, 1H), 8.33 (s, 1H), and 8.91 (s, 1H). ^13^C-NMR (100 MHz, CDCl_3_) δ 20.7, 55.6, 63.5, 66.2, 95.7, 122.9, 125.0 (2C), 127.6, 127.7, 127.9, 129.2, 129.4, 130.0 (2C), 130.4, 134.0, 135.6 (2C), 136.7, 146.0, 147.3, 151.4, and 170.3. MS *m*/*z*: 418 (M^+^). HRMS (EI): calcd for C_24_H_22_N_2_O_5_ 418.1529; found 418.1535.

#### 3.1.9. 3-{3-[(*tert*-Butyldimethylsilyloxy)methyl]quinolin-2-yl}-5-[(methoxymethoxy)methyl]isoquinolin-1-one (**17b**)

A solution of *N*-oxide **16b** (25 mg, 0.051 mmol) in Ac_2_O (1 mL) was heated at 80 °C under microwave irradiation for 2 h. After removal of the solvent, the residue was purified by column chromatography (EtOAc/hexane 2:3 *v*/*v*) to give the isoquinolone **17b** (9 mg, 36%) as a yellow solid. mp was 114–115 °C (EtOAc-hexane). IR (ATR) ν = 1651, 3305 cm^−1^. ^1^H-NMR (400 MHz, CDCl_3_) δ 0.21 (s, 6H), 1.00 (s, 9H), 3.40 (s, 3H), 4.73 (s, 2H), 4.97 (s, 2H), 5.20 (s, 2H), 7.43 (s, 1H), 7.53 (t, *J* = 7.8 Hz, 1H), 7.62 (t, *J* = 7.8 Hz, 1H), 7.75 (d, *J* = 7.8 Hz, 1H), 7.78 (t, *J* = 7.8 Hz, 1H), 7.90 (d, *J* = 7.8 Hz, 1H), 8.16 (d, *J* = 7.8 Hz, 1H), 8.49 (d, *J* = 7.8 Hz, 2H), and 10.73 (br s, 1H). ^13^C-NMR (100 MHz, CDCl_3_) δ-5.1 (2C), 18.4, 25.9 (3C), 55.5, 63.1, 66.7, 95.2, 105.1, 127.2, 127.3, 127.5, 127.7 (2C), 127.8, 129.2, 130.3, 132.4, 133.3, 133.6, 136.2, 136.4, 136.6, 146.2, 148.3, and 162.6. MS *m*/*z*: 490 (M^+^). HRMS (EI): calcd for C_28_H_34_N_2_O_4_Si 490.2288; found 490.2284.

#### 3.1.10. 3-(3-Acetoxymethylquinolin-2-yl)-5-[(methoxymethoxy)methyl]isoquinolin-1-one (**17c**) and 3-[3-(Acetoxymethyl)quinolin-2-yl]-4-hydroxy-3-[(methoxymethoxy)methyl]isoquinoline (**18c**)

A solution of *N*-oxide **16c** (30 mg, 0.071 mmol) in Ac_2_O (2 mL) was heated at 80 °C under MW irradiation for 5 h. After removal of the solvent, the residue was purified by column chromatography (EtOAc/hexane 1:1 *v*/*v*) to give the isoquinolone **17c** (15 mg, 50%) and the 4-acetoxyisoquinoline **18c** (11 mg, 37%).

**17c**: yellow solid, mp 149–150 °C (EtOAc-hexane). IR (ATR) ν = 1643, 1743, and 3286 cm^−1^. ^1^H-NMR (400 MHz, CDCl_3_) δ 2.25 (s, 3H), 3.39 (s, 3H), 4.71 (s, 2H), 4.95 (s, 2H), 5.54 (s, 2H), 7.54 (t, *J* = 7.8 Hz, 1H), 7.57 (s, 1H), 7.64 (t, *J* = 7.8 Hz, 1H), 7.75 (d, *J* = 7.8 Hz, 1H), 7.83 (t, *J* = 7.8 Hz, 1H), 7.90 (d, *J* = 7.8 Hz, 1H), 8.17 (d, *J* = 7.8 Hz, 1H), 8.45 (s, 1H), 8.50 (d, *J* = 7.8 Hz, 1H), and 10.36 (br s, 1H). ^13^C-NMR (100 MHz, CDCl_3_) δ 21.0, 55.5, 64.1, 66.6, 95.1, 105.3, 126.5, 127.2, 127.3, 127.4, 127.5, 127.8, 128.1, 129.2, 131.1, 133.4, 133.7, 135.3, 136.3, 140.7, 146.8, 149.7, 162.6, and 170.6. MS *m*/*z*: 418 (M^+^). HRMS (EI): calcd for C_24_H_22_N_2_O_5_ 418.1529; found 418.1523.

**18c**: yellow solid, mp 108–109 °C (EtOAc-hexane). IR (ATR) ν = 1732 cm^−1^. ^1^H-NMR (400 MHz, CDCl_3_) δ 2.08 (s, 3H), 3.47 (s, 3H), 4.80 (s, 2H), 5.16 (s, 2H), 5.71 (s, 2H), 7.60 (t, *J* = 7.8 Hz, 1H), 7.65 (t, *J* = 7.8 Hz, 1H), 7.76 (t, *J* = 7.8 Hz, 1H), 7.82 (d, *J* = 7.8 Hz, 1H), 7.90 (d, *J* = 7.8 Hz, 1H), 8.01 (d, *J* = 7.8 Hz, 1H), 8.20 (d, *J* = 7.8 Hz, 1H), 8.36 (s, 1H), 8.70 (s, 1H), and 9.36 (s, 1H). ^13^C-NMR (100 MHz, CDCl_3_) δ 21.0, 55.7, 64.3, 66.5, 95.9, 117.7, 127.0, 127.4 (2C), 127.5, 127.8, 128.3, 128.9, 129.4, 129.8, 130.4, 133.9, 135.0, 136.0, 147.2, 151.8, 152.2, 156.3, and 170.7. MS *m*/*z*: 418 (M^+^). HRMS (EI): calcd for C_24_H_22_N_2_O_5_ 418.1529; found 418.1518.

#### 3.1.11. 3-(3-Hydroxymethylquinolin-2-yl)-5-[(methoxymethoxy)methyl]isoquinolin-1-one (**19**)

To a solution of isoquinolone **17c** (9 mg, 0.020 mmol) in MeOH (1 mL), 1 M NaOH aq. (0.1 mL) was added dropwise under ice cooling and was stirred at 70 °C for 15 min. After cooling at ambient temperature, the resulting mixture was quenched with water and extracted with EtOAc. The organic layer was washed with water and brine, dried with Na_2_SO_4_, and evaporated in vacuo. The residue was purified by column chromatography (EtOAc/hexane 3:7 *v*/*v*) to give the alcohol **19** (7 mg, 93%) as a white solid. mp was 199–200 °C (EtOAc-hexane). IR (ATR) ν = 1620, 3286, 3737 cm^−1^. ^1^H-NMR (400 MHz, DMSO-*d*_6_) δ 3.28 (s, 3H), 4.70 (s, 2H), 4.80 (d, *J* = 5.0 Hz, 2H), 4.85 (s, 2H), 5.78 (t, *J* = 5.0 Hz, 1H), 7.16 (s, 1H), 7.54 (t, *J* = 7.8 Hz, 1H), 7.67 (t, *J* = 7.8 Hz, 1H), 7.77–7.83 (m, 2H), 8.08–8.12 (m, 2H), 8.26 (d, *J* = 7.8 Hz, 1H), 8.55 (s, 1H), and 11.48 (br s, 1H). ^13^C-NMR (100 MHz, DMSO-*d*_6_) δ 55.0, 60.5, 66.6, 95.5, 103.1, 126.2, 126.7, 126.8, 127.5 (2C), 127.6, 127.8, 128.8, 130.0, 133.0, 133.6, 133.9, 136.0, 138.0, 145.9, 151.3, and 161.9. MS *m*/*z*: 376 (M^+^). HRMS (EI): calcd for C_22_H_20_N_2_O_4_ 376.1423; found 376.1433.

#### 3.1.12. 22-Hydroxyacuminatine (**4**)

To a solution of alcohol **19** (8 mg, 0.021 mmol) and PPh_3_ (8 mg, 0.032 mmol) in THF (1 mL), DIAD (1.9 M in toluene, 0.017 mL, 0.032 mmol) was added dropwise under ice cooling, and was stirred at 0 °C for 1 h. The resulting mixture was quenched with water and extracted with EtOAc. The organic layer was washed with water and brine, dried with Na_2_SO_4_, and evaporated in vacuo. To a suspension of the residue and ethylene glycol (0.1 mL) in THF (0.5 mL), 6 M HCl (0.5 mL) was added dropwise under ice cooling and then stirred at 50 °C for 3 h. After cooling to ambient temperature, the residue was alkalified with 1 M aqueous NaOH. The resulting mixture was extracted with EtOAc. The organic layer was washed with water and brine, dried with Na_2_SO_4_, and evaporated in vacuo. The residue was filtered off to give 22-hydroxyacuminatine (**4**) (4 mg, 60%) as a white solid. mp 255–256 °C (EtOAc-hexane lit. [3] mp 258–260 °C). IR (ATR) ν = 1651, 3359 cm^−1^. ^1^H-NMR (400 MHz, DMSO-*d*_6_) δ 4.95 (d, *J* = 5.5 Hz, 2H), 5.36 (s, 2H), 5.50 (t, *J* = 5.5 Hz, 1H), 7.54–7.63 (m, 1H), 7.69 (t, *J* = 7.8 Hz, 1H), 7.73 (s, 1H), 7.81 (d, *J* = 7.8 Hz, 1H), 7.83–7.87 (m, 1H), 8.11 (d, *J* = 7.8 Hz, 1H), 8.19 (d, *J* = 7.8 Hz, 1H), 8.30 (d, *J* = 7.8 Hz, 1H), and 8.66 (s, 1H). ^13^C-NMR (100 MHz, DMSO-*d*_6_) δ 49.6, 61.2, 96.2, 125.9 (2C), 126.7, 127.3, 127.9, 128.5, 128.9, 129.7, 130.2, 131.1, 131.3, 135.3, 138.5, 140.1, 148.0, 153.4, and 159.9. MS *m*/*z*: 314 (M^+^). HRMS (EI): calcd for C_20_H_14_N_2_O_2_ 314.1055; found 314.1061.

#### 3.1.13. Methyl [2-Iodo-N-(methoxymethyl)indol-3-yl]acetate (**22**)

A solution of methyl indolylacetate **21** (470 mg, 1.49 mmol) in DMF (8 mL) was added dropwise to a suspension of NaH (71 mg, 1.79 mmol) in DMF (4 mL) under ice cooling. After stirring at same temperature for 30 min, MOMCl (0.13 mL, 1.79 mmol) was added to the reaction mixture and then was stirred at rt for 2 h. After quenching with H_2_O, the reaction mixture was extracted with EtOAc. The organic layer was washed with brine, dried with Na_2_SO_4_, and evaporated in vacuo. The residue was purified by column chromatography (EtOAc/hexane 1:3 *v*/*v*) to give the *N*-MOM-indolylacetate **22** (454 mg, 85%) as a yellow solid. mp was 83–85 °C (EtOAc-hexane). IR (ATR) ν = 1728 cm^−1^. ^1^H-NMR (400 MHz, CDCl_3_) δ 3.31 (s, 3H), 3.69 (s, 3H), 3.79 (s, 2H), 5.54 (s, 2H), 7.16 (t, *J* = 7.8 Hz, 1H), 7.20 (t, *J* = 7.8 Hz, 1H), 7.48 (d, *J* = 7.8 Hz, 1H), and 7.55 (d, *J* = 7.8 Hz, 1H). ^13^C-NMR (100 MHz, CDCl_3_) δ 33.6, 52.1, 56.0, 77.5, 88.0, 110.1, 116.1, 118.5, 120.7, 122.7, 128.4, 138.6, and 171.3. MS *m*/*z*: 359 (M^+^). HRMS (EI): calcd for C_13_H_14_INO_3_ 359.0018; found 359.0022.

#### 3.1.14. Methyl [2-Trimethylsilylethynyl-N-(methoxymethyl)lindol-3-yl]acetate (**23**)

A solution of 2-iodoindole **22** (200 mg, 0.56 mmol), CuI (11 mg, 0.056 mmol), PdCl_2_(PPh_3_)_2_ (23 mg, 0.022 mmol), and TMS-acetylene (0.091 mL, 0.66 mmol) in Et_3_N (5 mL, 35.6 mmol) was stirred at rt for 23 h. Afterward, the reaction mixture was filtered through Celite pad, washed with EtOAc, and the filtrate was evaporated in vacuo. The residue was purified by column chromatography (EtOAc/hexane 1:9 *v*/*v*) to give the TMS-acetylene **23** (143 mg, 78%) as a yellow oil. IR (ATR) ν = 1736, 2152 cm^−1^. ^1^H-NMR (400 MHz, CDCl_3_) δ 0.30 (s, 9H), 3.28 (s, 3H), 3.70 (s, 3H), 3.87 (s, 2H), 5.57 (s, 2H), 7.17 (t, *J* = 7.8 Hz, 1H), 7.30 (t, *J* = 7.8 Hz, 1H), 7.44 (d, *J* = 7.8 Hz, 1H), and 7.57 (d, *J* = 7.8 Hz, 1H). ^13^C-NMR (100 MHz, CDCl_3_) δ -0.14 (3C), 31.2, 52.0, 56.0, 75.1, 94.4, 105.3, 110.3, 115.1, 119.6, 121.0, 121.3, 124.1, 127.1, 136.6, and 171.6. MS *m*/*z*: 329 (M^+^). HRMS (EI): calcd for C_18_H_23_NO_3_Si 329.1447; found 329.1451.

#### 3.1.15. Methyl [2-Ethynyl-N-(methoxymethyl)indol-3-yl]acetate (**24**)

A solution of TBAF (1.0 M in THF, 2.19 mL, 2.19 mmol) was added dropwise to a solution of TMS-acetylene **23** (600 mg, 1.82 mmol) in THF (20 mL) at 0 °C. After being stirred at rt for 10 min, the reaction mixture was quenched with water and then extracted with EtOAc. The organic layer was washed with brine, dried over Na_2_SO_4_, and evaporated in vacuo. The residue was purified by column chromatography using EtOAc-hexane (1:4, *v*/*v*) as an eluent to give the 2-ethynylindole **24** (430 mg, 92%) as a red solid. mp was 92–94 °C (EtOAc-hexane). IR (ATR) ν = 1732, 2102 cm^−1^. ^1^H-NMR (400 MHz, CDCl_3_) δ 3.29 (s, 3H), 3.68 (s, 1H), 3.70 (s, 3H), 3.90 (s, 2H), 5.59 (s, 2H), 7.19 (t, *J* = 7.8 Hz, 1H), 7.32 (t, *J* = 7.8 Hz, 1H), 7.45 (d, *J* = 7.8 Hz, 1H), and 7.60 (d, *J* = 7.8 Hz, 1H). ^13^C-NMR (100 MHz, CDCl_3_) δ 31.0, 52.1, 56.0, 73.9, 75.1, 86.9, 110.3, 115.8, 119.7, 120.1, 121.1, 124.3, 127.0, 136.7, and 171.4. MS *m*/*z*: 257 (M^+^). HRMS (EI): calcd for C_15_H_15_NO_3_ 257.1052; found 257.1046.

#### 3.1.16. Methyl {2-[2-(2-Formylphenyl)ethynyl]-N-(methoxymethyl)indol-3-yl}acetate (**26a**)

A solution of 2-ethynylindole **24** (81 mg, 0.32 mmol), CuI (2.7 mg, 0.014 mmol), PdCl_2_(PPh_3_)_2_ (6 mg, 0.0086 mmol), and 2-iodobenzaldehyde **25a** (66 mg, 0.29 mmol) in Et_3_N (2.6 mL, 18 mmol) was stirred at 70 °C for 45 min. Afterward, the reaction mixture was filtered through Celite pad, washed with EtOAc, and the filtrate was evaporated in vacuo. The residue was purified by column chromatography (EtOAc/hexane 1:9 *v*/*v*) to give the 2-alkynylbenzaldehyde **26a** (83 mg, 80%) as a yellow solid. mp was 75–77 °C (EtOAc-hexane). IR (ATR) ν = 1689, 1736, and 2202 cm^−1^. ^1^H-NMR (400 MHz, CDCl_3_) δ 3.35 (s, 3H), 3.71 (s, 3H), 3.98 (s, 2H), 5.70 (s, 2H), 7.22 (t, *J* = 7.8 Hz, 1H), 7.35 (t, *J* = 7.8 Hz, 1H), 7.49 (d, *J* = 7.8 Hz, 1H), 7.53 (d, *J* = 7.8 Hz, 1H), 7.61–7.66 (m, 2H), 7.71 (d, *J* = 7.8 Hz, 1H), 7.97 (d, *J* = 7.8 Hz, 1H), and 10.59 (s, 1H). ^13^C-NMR (100 MHz, CDCl_3_) δ 31.3, 52.2, 56.2, 75.3, 86.1, 95.1, 110.3, 116.3, 119.8, 120.5, 121.2, 124.6, 125.2, 127.3, 128.6, 129.0, 133.3, 133.8, 135.6, 137.2, 171.3, and 191.1. MS *m*/*z*: 361 (M^+^). HRMS (EI): calcd for C_22_H_19_NO_4_ 361.1314; found 361.1322.

#### 3.1.17. Methyl {2-[2-(2-Formyl-3-[(methoxymethoxy)methyl]phenyl)ethynyl]-N-(methoxymethylindol-3-yl}acetate (**26b**)

A solution of 2-ethynylindole **24** (266 mg, 1.04 mmol), CuI (20 mg, 0.10 mmol), PdCl_2_(PPh_3_)_2_ (36 mg, 0.052 mmol), and 2-iodobenzaldehyde **25b** (377 mg, 1.25 mmol) in *i*Pr_2_NH (8 mL) was stirred at 100 °C for 3 h. Afterward, the reaction mixture was filtered through Celite pad, washed with EtOAc, and the filtrate was evaporated in vacuo. The residue was purified by column chromatography (EtOAc/hexane 1:9 *v*/*v*) to give the 2-alkynylbenzaldehyde **26b** (410 mg, 91%) as a yellow solid. mp was 76–78 °C (EtOAc-hexane). IR (ATR) ν = 1693, 1724, 2198 cm^−1^. ^1^H-NMR (400 MHz, CDCl_3_) δ 3.34 (s, 3H), 3.42 (s, 3H), 3.71 (s, 3H), 4.00 (s, 2H), 4.80 (s, 2H), 4.98 (s, 2H), 5.71 (s, 2H), 7.21 (d, *J* = 7.8 Hz, 1H), 7.35 (t, *J* = 7.8 Hz, 1H), 7.49 (d, *J* = 7.8 Hz, 1H), 7.53 (d, *J* = 7.8 Hz, 1H), 7.66 (d, *J* = 7.8 Hz, 1H), 7.83 (d, *J* = 7.8 Hz, 1H), 7.91 (d, *J* = 7.8 Hz, 1H), and 10.64 (s, 1H). ^13^C-NMR (100 MHz, CDCl_3_) δ 31.3, 52.2, 55.5, 56.0, 66.8, 75.3, 91.3, 92.2, 96.1, 110.3, 116.6, 119.8, 120.3, 121.2, 123.5, 124.7, 127.3, 127.7, 128.7, 132.9, 135.9, 137.4, 141.2, 171.2, and 191.2. MS *m*/*z*: 435 (M^+^). HRMS (EI): calcd for C_25_H_25_NO_6_ 435.1682; found 435.1696.

#### 3.1.18. Methyl {2-[22-Hydroxyiminophenyl)ethynyl]-N-(methoxymethyl)indol-3-yl}acetate (**27a**)

A mixture of benzaldehyde **26a** (184 mg, 0.51 mmol), NH_2_OH·HCl (70 mg, 1.02 mmol), and AcONa (83 mg, 1.02 mmol) in EtOH (4 mL) was stirred at rt for 1.5 h. After removal of solvent, the residue was diluted with H_2_O and then filtered off to give the oxime **27a** (170 mg, 88%) as a yellow solid. mp was 110–113 °C (EtOAc-hexane). IR (ATR) ν = 1701, 1732, 2218, and 3062 cm^−1^. ^1^H-NMR (400 MHz, CDCl_3_) δ 3.36 (s, 3H), 3.73 (s, 3H), 4.03 (s, 2H), 5.68 (s, 2H), 7.22 (t, *J* = 7.8 Hz, 1H), 7.33 (t, *J* = 7.8 Hz, 1H), 7.38–7.40 (m, 2H), 7.49 (d, *J* = 7.8 Hz, 1H), 7.60–7.62 (m, 1H), 7.67 (d, *J* = 7.8 Hz, 1H), 7.70–7.72 (m, 1H), 8.46 (br s, 1H), and 8.59 (s, 1H). ^13^C-NMR (100 MHz, CDCl_3_) δ 31.5, 52.4, 56.2, 75.3, 84.4, 97.0, 110.2, 115.3, 119.8, 121.1, 121.2, 121.2, 124.3, 127.2, 127.4, 128.9, 129.3, 132.8, 133.0, 137.1, 148.8, and 172.3. MS *m*/*z*: 376 (M^+^). HRMS (EI): calcd for C_22_H_20_N_2_O_4_ 376.1423; found 376.1427.

#### 3.1.19. Methyl {2-[2-(2-Hydroxyimino-3-[(methoxymethoxy)methyl]phenyl)ethynyl]-N-methoxymethylindol-3-yl}acetate (**27b**)

The same procedure as above was carried out with 2-arylethynylbenzaldehyde **26b** (450 mg, 1.03 mmol) to give the oxime **27b** (414 mg, 89%) as a white solid. Mp was 94–95 °C (EtOAc-hexane). IR (ATR) ν = 1736, 2164, and 3302 cm^−1^. ^1^H-NMR (400 MHz, CDCl_3_) δ 3.35 (s, 3H), 3.42 (s, 3H), 3.71 (s, 3H), 4.03 (s, 2H), 4.78 (s, 2H), 4.92 (s, 2H), 5.68 (s, 2H), 7.21 (t, *J* = 7.8 Hz, 1H), 7.33 (t, *J* = 7.8 Hz, 1H), 7.39 (t, *J* = 7.8 Hz, 1H), 7.48 (d, *J* = 7.8 Hz, 1H), 7.57 (d, *J* = 7.8 Hz, 1H), 7.66–7.70 (m, 2H), 8.45 (s, 1H), and 8.68 (s, 1H). ^13^C-NMR (100 MHz, CDCl_3_) δ 31.4, 52.3, 55.4, 56.0, 67.4, 75.3, 89.8, 93.9, 95.9, 110.2, 115.6, 119.9 (2C), 121.0, 121.1, 124.4, 126.0, 127.4, 128.7, 128.7, 133.5, 137.3, 140.6, 149.0, and 172.0. MS *m*/*z*: 450 (M^+^). HRMS (EI): calcd for C_25_H_26_N_2_O_6_ 450.1791; found 450.1799.

#### 3.1.20. 3-[3-(2-Methoxy-2-oxoethyl)-1-(methoxymethyl)indol-2-yl]isoquinoline N-oxide (**28a**)

A solution of oxime **27a** (45 mg, 0.12 mmol) in 1,2-dichlorobenzene (3 mL) was stirred at 120 °C for 17 h. After removal of the solvent, the residue was purified by column chromatography (EtOAc/hexane 1:1 *v*/*v*) to give the *N*-oxide **28a** (37 mg, 82%) as a brown oil. IR (ATR) ν = 1315, 1736 cm^−1^. ^1^H-NMR (400 MHz, CDCl_3_) δ 3.09 (s, 3H), 3.55 (d, *J* = 15.6 Hz, 1H), 3.69 (s, 3H), 3.83 (d, *J* = 15.6 Hz, 1H), 5.35 (d, *J* = 11.4 Hz, 1H), 5.67 (d, *J* = 11.4 Hz, 1H), 7.22 (t, *J* = 7.8 Hz, 1H), 7.34 (t, *J* = 7.8 Hz, 1H), 7.55 (d, *J* = 7.8 Hz, 1H), 7.62–7.71 (m, 3H), 7.79 (d, *J* = 7.8 Hz, 1H), 7.88 (d, *J* = 7.8 Hz, 1H), 8.17 (s, 1H), and 8.94 (s, 1H). ^13^C-NMR (100 MHz, CDCl_3_) δ 30.9, 52.1, 56.0, 76.4, 110.4, 111.1, 119.7, 120.5, 123.8, 124.6, 127.2, 127.6, 128.5, 129.0, 129.2, 129.7, 129.8, 129.9, 137.0, 137.4, 138.2, and 172.1. MS *m*/*z*: 376 (M^+^). HRMS (EI): calcd for C_22_H_20_N_2_O_4_ 376.1423; found 376.1441.

#### 3.1.21. 3-[3-(2-Methoxy-2-oxoethyl)-1-(methoxymethyl)indol-2-yl)-5-[(methoxymethoxy)methyl]isoquinoline N-oxide (**28b**)

The same procedure as above was carried out with the oxime **27b** (30 mg, 0.067 mmol) to give the *N*-oxide **28b** (21 mg, 69%) as a brown oil. IR (ATR) ν = 1323, 1731 cm^−1^. ^1^H-NMR (400 MHz, CDCl_3_) δ 3.10 (s, 3H), 3.36 (s, 3H), 3.54 (d, *J* = 15.6 Hz, 1H), 3.70 (s, 3H), 3.84 (d, *J* = 15.6 Hz, 1H), 4.73 (s, 2H), 4.94 (d, *J* = 12.3 Hz, 1H), 5.05 (d, *J* = 12.3 Hz, 1H), 5.37 (d, *J* = 11.0 Hz, 1H), 5.67 (d, *J* = 11.0 Hz, 1H), 7.21 (t, *J* = 7.8 Hz, 1H), 7.34 (t, *J* = 7.8 Hz, 1H), 7.55 (d, *J* = 7.8 Hz, 1H), 7.62–7.68 (m, 2H), 7.71 (d, *J* = 7.8 Hz, 1H), 7.75 (d, *J* = 7.8 Hz, 1H), 8.43 (s, 1H) and 8.94 (s, 1H). ^13^C-NMR (100 MHz, CDCl_3_) δ 31.1, 52.1, 55.6, 56.0, 66.3, 95.9, 110.5, 111.2, 119.8 (2C), 120.6, 123.9, 124.9, 126.1, 127.1, 127.7, 129.3, 129.7, 129.9, 130.4, 134.3, 137.2, 137.5, 138.1 and 172.0. MS *m*/*z*: 450 (M^+^). HRMS (EI): calcd for C_25_H_26_N_2_O_6_ 450.1791; found 450.1795.

#### 3.1.22. Methyl 2-[1-(methoxymethyl)-2-(1-oxo-1,2-dihydroisoquinolin-3-yl)indol-3-yl]acetate (**29a**) and Methyl 2-[2-(4-acetoxyisoquinolin-3-yl)-1-(methoxymethyl)indol-3-yl]acetate (**31a**)

A solution of *N*-oxide **28a** (15 mg, 0.040 mmol) in Ac_2_O (1 mL) was heated at 80 °C under MW irradiation for 6 h. After removal of the solvent, the residue was purified by column chromatography (EtOAc/hexane 1:1 *v*/*v*) to give the isoquinolone **29a** (9 mg, 60%) and the 4-acetoxyisoquinoline **31a** (3 mg, 18%).

**29a**: White solid, mp was 150–151 °C (EtOAc-hexane). IR (ATR) ν = 1624, 1739, and 3649 cm^−1^. ^1^H-NMR (400 MHz, CDCl_3_) δ 3.46 (s, 3H), 3.79 (s, 3H), 3.86 (s, 2H), 5.48 (s, 2H), 6.90 (s, 1H), 7.26 (t, *J* = 7.8 Hz, 1H), 7.37 (t, *J* = 7.8 Hz, 1H), 7.51–7.57 (m, 2H), 7.63 (d, *J* = 7.8 Hz, 1H), 7.67–7.73 (m, 2H), 8.47 (d, *J* = 7.8 Hz, 1H), and 9.98 (br s, 1H). ^13^C-NMR (100 MHz, CDCl_3_) δ 30.9, 52.6, 56.5, 74.9, 109.2, 109.9, 110.5, 119.6, 121.3, 124.4, 125.9, 126.8, 127.4 (2C), 127.7, 129.6, 132.3, 132.9, 137.4, 137.6, 162.8, and 172.8. MS *m*/*z*: 376 (M^+^). HRMS (EI): calcd for C_22_H_20_N_2_O_4_ 376.1423; found 376.1431.

**31a**: Brown oil. IR (ATR) ν = 1732, 1774 cm^−1^. ^1^H-NMR (400 MHz, CDCl_3_) δ 2.16 (s, 3H), 3.16 (s, 3H), 3.63 (s, 3H), 3.64–3.75 (m, 2H), 5.34 (d, *J* = 11.0 Hz, 1H), 5.56 (d, *J* = 11.0 Hz, 1H), 7.22 (t, *J* = 7.8 Hz, 1H), 7.32 (t, *J* = 7.8 Hz, 1H), 7.59 (d, *J* = 7.8 Hz, 1H), 7.63 (d, *J* = 7.8 Hz, 1H), 7.74 (t, *J* = 7.8 Hz, 1H), 7.82 (t, *J* = 7.8 Hz, 1H), 7.88 (d, *J* = 7.8 Hz, 1H), 8.11 (d, *J* = 7.8 Hz, 1H), and 9.30 (s, 1H). ^13^C-NMR (100 MHz, CDCl_3_) δ 20.4, 30.7, 51.8, 55.9, 75.5, 109.7, 110.8, 119.6, 120.5, 121.1, 123.1, 127.8, 128.0, 128.5, 129.4, 130.5, 131.4, 132.8, 136.5, 137.7, 142.2, 150.3, 168.5, and 172.0. MS *m*/*z*: 418 (M^+^). HRMS (EI): calcd for C_24_H_22_N_2_O_5_ 418.1529; found 418.1527.

#### 3.1.23. Methyl 2-{2-[5-[(Methoxymethoxy)methyl]-1-oxo-1,2-dihydroisoquinolin-3-yl]-1-(methoxymethyl)indol-3-yl}acetate (**29b**), 1-Acetoxy-3-[3-methoxycarbonylmethyl-N-(methoxymethyl)indol-2-yl]-5-[(methoxymethoxy)methyl]isoquinoline (**30b**) and 4-Acetoxy-3-[3-methoxycarbonylmethyl-N-(methoxymethyl)indol-2-yl]-5-[(methoxymethoxy)methyl]isoquinoline (**31b**)

The same procedure as above was carried out with the oxime **28b** (32 mg, 0.071 mmol) to give the isoquinolone **29b** (12 mg, 38%), the 1-acetoxyisoquinoline **30b** (7 mg, 20%), and the 4-acetoxyisoquinoline **31b** (11 mg, 32%).

**29b:** Orange oil. IR (ATR) ν = 1643, 1736, and 3672 cm^−1^. ^1^H-NMR (400 MHz, CDCl_3_) δ 3.39 (s, 3H), 3.49 (s, 3H), 3.79 (s, 3H), 3.87 (s, 2H), 4.73 (s, 2H), 4.89 (s, 2H), 5.49 (s, 2H), 7.17 (s, 1H), 7.26 (t, *J* = 7.8 Hz, 1H), 7.38 (t, *J* = 7.8 Hz, 1H), 7.50–7.54 (m, 2H), 7.69 (d, *J* = 7.8 Hz, 1H), 7.75 (d, *J* = 7.8 Hz, 1H), 8.47 (d, *J* = 7.8 Hz, 1H), and 10.11 (br s, 1H). ^13^C-NMR (100 MHz, CDCl_3_) δ 30.9, 52.6, 55.5, 56.5, 66.8, 74.9, 95.8, 105.7, 109.9, 110.7, 119.7, 121.3, 124.4, 126.4, 126.9, 127.5, 127.8, 129.8, 132.5, 133.3, 133.4, 136.2, 137.7, 162.8, and 172.7. MS *m*/*z*: 450 (M^+^). HRMS (EI): calcd for C_25_H_26_N_2_O_6_ 450.1791; found 450.1783.

**30b**: Orange oil. IR (ATR) ν = 1736, 1778 cm^−1^. ^1^H-NMR (400 MHz, CDCl_3_) δ 2.51 (s, 3H), 3.18 (s, 3H), 3.41 (s, 3H), 3.74 (s, 3H), 3.88 (s, 2H), 4.78 (s, 2H), 5.08 (s, 2H), 5.75 (s, 2H), 7.22 (t, *J* = 7.8 Hz, 1H), 7.32 (t, *J* = 7.8 Hz, 1H), 7.56 (d, *J* = 7.8 Hz, 1H), 7.65 (t, *J* = 7.8 Hz, 1H), 7.72 (d, *J* = 7.8 Hz, 1H), 7.85 (d, *J* = 7.8 Hz, 1H), 8.03 (d, *J* = 7.8 Hz, 1H), and 8.39 (s, 1H). ^13^C-NMR (100 MHz, CDCl_3_) δ 21.3, 31.3, 52.2, 55.6, 56.0, 66.6, 75.2, 96.0, 109.8, 110.6, 118.8, 119.7 (2C), 120.8, 123.6, 123.7, 127.9, 128.2, 131.4, 134.3, 136.5, 137.9, 137.9, 141.7, 155.7, 168.9, and 172.5. MS *m*/*z*: 492 (M^+^). HRMS (EI): calcd for C_27_H_28_N_2_O_7_ 492.1897; found 492.1891.

**31b**: Yellow oil. IR (ATR) ν = 1736, 1774 cm^−1^. ^1^H-NMR (400 MHz, CDCl_3_) δ 1.99 (s, 3H), 3.28 (s, 3H), 3.36 (s, 3H), 3.63 (s, 3H), 3.64 (d, *J* = 16.5 Hz, 1H), 3.73 (d, *J* = 16.5 Hz, 1H), 4.61 (d, *J* = 6.9 Hz, 1H), 4.64 (d, *J* = 6.9 Hz, 1H), 5.02 (d, *J* = 13.3 Hz, 1H), 5.21 (d, *J* = 13.3 Hz, 1H), 5.26 (d, *J* = 11.0 Hz, 1H), 5.57 (d, *J* = 11.0 Hz, 1H), 7.21 (t, *J* = 7.8 Hz, 1H), 7.31 (t, *J* = 7.8 Hz, 1H), 7.61 (d, *J* = 7.8 Hz, 1H), 7.65 (d, *J* = 7.8 Hz, 1H), 7.69 (t, *J* = 7.8 Hz, 1H), 7.88 (d, *J* = 7.8 Hz, 1H), 8.05 (d, *J* = 7.8 Hz, 1H), and 9.28 (s, 1H). ^13^C-NMR (100 MHz, CDCl_3_) δ 21.0, 30.8, 51.8, 55.4, 56.1, 67.7, 75.9, 94.7, 109.3, 111.0, 119.6 (2C), 120.4, 123.0, 128.0, 128.3, 129.5, 130.7, 132.0, 133.1, 133.2, 137.7, 138.8, 142.3, 150.8, 168.8, and 172.0. MS *m*/*z*: 492 (M^+^). HRMS (EI): calcd for C_27_H_28_N_2_O_7_ 492.1897; found 492.1889.

#### 3.1.24. 3-[3-(2-Hydroxyethyl)-N-(methoxymethyl)indol-2-yl]isoquinolin-1-one (**32a**)

A solution of the isoquinolone **29a** (25 mg, 0.066 mmol) in THF (2 mL) was added dropwise to a suspension of LiAlH_4_ (4 mg, 0.10 mmol) in THF (2 mL) under ice cooling and then stirred at 70 °C for 30 min. After quenching with H_2_O, the reaction mixture was filtered through Celite pad and washed with H_2_O and EtOAc, and the filtrate was extracted with EtOAc. The organic layer was washed with brine, dried with Na_2_SO_4_, and evaporated in vacuo. The residue was purified by column chromatography (EtOAc/hexane 1:1 *v*/*v*) to give the alcohol **32a** (22 mg, 95%) as a white solid. mp was 211–212 °C (EtOAc-hexane). IR (ATR) ν = 1732, 3351, 3629 cm^−1^. ^1^H-NMR (400 MHz, CDCl_3_) δ 3.12 (t, *J* = 5.5 Hz, 2H), 3.39 (s, 3H), 3.57 (br s, 1H), 4.12 (t, *J* = 5.5 Hz, 2H), 5.47 (s, 2H), 6.89 (s, 1H), 7.24 (t, *J* = 7.8 Hz, 1H), 7.36 (t, *J* = 7.8 Hz, 1H), 7.48–7.54 (m, 2H), 7.61–7.63 (m, 2H), 7.69 (t, *J* = 7.8 Hz, 1H), 8.39 (d, *J* = 7.8 Hz, 1H), and 11.36 (br s, 1H). ^13^C-NMR (100 MHz, CDCl_3_) δ 27.1, 56.2, 61.7, 74.9, 108.4, 110.4, 114.4, 119.2, 120.9, 124.0, 125.5, 126.7, 127.0, 127.4, 127.5, 130.4, 132.5, 132.7, 137.7, 138.3, and 163.3. MS *m*/*z*: 348 (M^+^). HRMS (EI): calcd for C_21_H_20_N_2_O_3_ 348.1474; found 348.1468.

#### 3.1.25. 3-[3-(2-Hydroxyethyl)-N-(methoxymethyl)indol-2-yl]-5-[(methoxymethoxy)methyl]isoquinolin-1-one (**32b**)

A solution of N-oxide 28b (25 mg, 0.056 mmol) in Ac_2_O (1 mL) was heated at 80 °C under MW irradiation for 4.5 h. After removal of the solvent, the residue was purified by column chromatography (EtOAc/hexane 1:3 *v*/*v*) to give a mixture of isoquinolone 29b and the 1-acetoxyisoquinoline 30b. A solution of the mixture in THF (1 mL) was added dropwise to a suspension of LiAlH_4_ (4 mg, 0.11 mmol) in THF (1 mL) under ice cooling and then stirred at 0 °C for 1 h. After quenching with H_2_O, the reaction mixture was filtered through Celite pad and washed with H_2_O and EtOAc, and the filtrate was extracted with EtOAc. The organic layer was washed with brine, dried with Na_2_SO_4_, and evaporated in vacuo. The residue was purified by column chromatography (EtOAc/hexane 1:1 *v*/*v*) to give the alcohol 32b (16 mg, 69%) as a white solid. mp was 175–176 °C (EtOAc-hexane). IR (ATR) ν = 1639, 3309, and 3529 cm^−1^. ^1^H-NMR (400 MHz, CDCl_3_) δ 3.11 (t, J = 5.5 Hz, 2H), 3.39 (s, 3H), 3.42 (s, 3H), 4.12 (t, J = 5.5 Hz, 2H), 4.73 (s, 2H), 4.87 (s, 2H), 5.46 (s, 2H), 7.16 (s, 1H), 7.23 (t, J = 7.8 Hz, 1H), 7.35 (t, J = 7.8 Hz, 1H), 7.44 (t, J = 7.8 Hz, 1H), 7.52 (d, J = 7.8 Hz, 1H), 7.62 (d, J = 7.8 Hz, 1H), 7.71 (d, J = 7.8 Hz, 1H), 8.35 (d, J = 7.8 Hz, 1H), and 11.63 (br s, 1H). ^13^C-NMR (100 MHz, CDCl_3_) δ 27.1, 55.5, 56.2, 61.7, 67.0, 75.0, 95.8, 104.9, 110.3, 114.8, 119.3, 120.9, 124.0, 126.0, 126.4, 127.5, 127.7, 130.7, 132.6, 132.9, 133.3, 136.5, and 138.4, 163.4. MS m/z: 422 (M^+^). HRMS (EI): calcd for C_24_H_26_N_2_O_5_ 422.1842; found 422.1844.

#### 3.1.26. N-Methoxymethyl-3,14,15,16,17,18,19,20-octadehydroyohimban-21-one (**33a**)

To a solution of alcohol **32a** (21 mg, 0.060 mmol) and PPh_3_ (47 mg, 0.18 mmol) in THF (3 mL), DIAD (1.9 M in toluene, 0.94 mL, 0.18 mmol) was added dropwise under ice cooling and was stirred at 60 °C for 3 h. After cooling at ambient temperature, the resulting mixture was quenched with water and extracted with EtOAc. The organic layer was washed with water and brine, dried with Na_2_SO_4_, and evaporated in vacuo. The residue was purified by column chromatography (EtOAc/hexane 1:3 *v*/*v*) to give the indoloquinolizine **33a** (19 mg, 95%) as a white solid. mp was 163–166 °C (EtOAc-hexane). IR (ATR) ν = 1732 cm^−1^. ^1^H-NMR (400 MHz, CDCl_3_) δ 3.10 (t, *J* = 6.2 Hz, 2H), 3.59 (s, 3H), 4.50 (t, *J* = 6.2 Hz, 2H), 5.62 (s, 2H), 7.24 (t, *J* = 7.8 Hz, 1H), 7.31 (s, 1H), 7.37 (t, *J* = 7.8 Hz, 1H), 7.46 (t, *J* = 7.8 Hz, 1H), 7.55 (d, *J* = 7.8 Hz, 1H), 7.59–7.66 (m, 3H), and 8.45 (d, *J* = 7.8 Hz, 1H). ^13^C-NMR (100 MHz, CDCl_3_) δ 20.1, 40.4, 56.1, 75.1, 103.4, 109.9, 116.5, 119.4, 121.1, 124.5, 125.2, 125.4, 126.6, 128.0 (2C), 130.7, 131.1, 132.3, 136.4, 140.6, and 162.5. MS *m*/*z*: 330 (M^+^). HRMS (EI): calcd for C_21_H_18_N_2_O_2_ 330.1368; found 330.1354.

#### 3.1.27. N-Methoxymethyl-16-[(methoxymethoxy)methyl]-3,14,15,16,17,18,19,20-octadehydroyohimban-21-one (**33b**)

The same procedure as above was carried out with alcohol **32b** (35 mg, 0.083 mmol) to give the indoloquinolizine **33b** (30 mg, 90%) as a yellow solid. mp was 128–129 °C (EtOAc-hexane). IR (ATR) ν = 1736 cm^−1^. ^1^H-NMR (400 MHz, CDCl_3_) δ 3.10 (t, *J* = 6.4 Hz, 2H), 3.43 (s, 3H), 3.60 (s, 3H), 4.49 (t, *J* = 6.4 Hz, 2H), 4.79 (s, 2H), 4.90 (s, 2H), 5.63 (s, 2H), 7.24 (t, *J* = 7.8 Hz, 1H), 7.37 (t, *J* = 7.8 Hz, 1H), 7.44 (t, *J* = 7.8 Hz, 1H), 7.55 (d, *J* = 7.8 Hz, 1H), 7.58 (s, 1H), 7.63 (d, *J* = 7.8 Hz, 1H), 7.68 (d, *J* = 7.8 Hz, 1H), and 8.46 (d, *J* = 7.8 Hz, 1H). ^13^C-NMR (100 MHz, CDCl_3_) δ 20.1, 40.4, 55.4, 56.1, 66.9, 75.3, 95.9, 99.9, 110.0, 116.6, 119.4, 121.2, 124.6, 125.4, 125.7, 126.1, 128.3, 130.9, 131.3, 132.8, 132.9, 135.2, 140.7, and 162.5. MS *m*/*z*: 404 (M^+^). HRMS (EI): calcd for C_24_H_24_N_2_O_4_ 404.1736; found 404.1744.

#### 3.1.28. Norketoyobyrine (**5**)

To a suspension of indoloquinolizine **33a** (23 mg, 0.070 mmol) and ethylene glycol (0.78 mg, 0.014 mmol) in THF (5 mL), 6 M HCl (3 mL) was added dropwise under ice cooling and then stirred at 60 °C for 15 h. After cooling to ambient temperature, the solvent was evaporated in vacuo. The residue was diluted with water and then was alkalified with 1 M aqueous NaOH. The resulting mixture was extracted with EtOAc. The organic layer was washed with water and brine, dried with Na_2_SO_4_, and evaporated in vacuo. The residue was purified by column chromatography (EtOAc/hexane 1:1 *v*/*v*) to give norketoyobyrine (**5**; 18 mg, 90%) as a yellow solid. mp was 298–299 °C (EtOAc-hexane lit. [16] mp of 299–300 °C). IR (ATR) ν = 1732 cm^−1^. ^1^H-NMR (400 MHz, DMSO-*d*_6_) δ 3.08 (t, *J* = 6.6 Hz, 2H), 4.39 (t, *J* = 6.6 Hz, 2H), 7.06 (t, *J* = 7.8 Hz, 1H), 7.06 (s, 1H), 7.21 (t, *J* = 7.8 Hz, 1H), 7.42 (d, *J* = 7.8 Hz, 1H), 7.45 (t, *J* = 7.8 Hz, 1H), 7.55–7.63 (m, 2H), 7.70 (t, *J* = 7.8 Hz, 1H), 8.23 (d, *J* = 7.8 Hz, 1H), and 11.69 (br s, 1H). ^13^C-NMR (100 MHz, DMSO-*d*_6_) δ 19.3, 40.4, 99.1, 111.7, 112.5, 119.1, 119.5, 123.5, 124.5, 125.6, 126.0, 126.2, 127.5, 128.2, 132.4, 132.7, 136.2, 138.0, and 161.3. MS *m*/*z*: 286 (M^+^). HRMS (EI): calcd for C_19_H_14_N_2_O 286.1106; found 286.1114.

#### 3.1.29. 16-Hydroxymethyl-3,14,15,16,17,18,19,20-octadehydroyohimban-21-one (**34**)

The same procedure as above was carried out with alcohol **33b** (40 mg, 0.099 mmol) to give alcohol **34** (28 mg, 90%) as a white solid. Mp was 283–284 °C (EtOAc-hexane lit. [30] mp of 280–283 °C). IR (ATR) ν = 1747, 3317 cm^−1^. ^1^H-NMR (400 MHz, DMSO-*d*_6_) δ 3.08 (t, *J* = 6.6 Hz, 2H), 4.39 (t, *J* = 6.6 Hz, 2H), 4.87 (d, *J* = 5.5 Hz, 2H), 5.40 (t, *J* = 5.5 Hz, 1H), 7.06 (t, *J* = 7.8 Hz, 1H), 7.19–7.23 (m, 2H), 7.40–7.45 (m, 2H), 7.58 (d, *J* = 7.8 Hz, 1H), 7.74 (d, *J* = 7.8 Hz, 1H), 8.15 (d, *J* = 7.8 Hz, 1H), and 11.72 (br s, 1H). ^13^C-NMR (100 MHz, DMSO-*d*_6_) δ 19.3, 40.4, 60.4, 95.6, 111.6, 112.5, 119.2, 119.5, 123.6, 124.7, 125.6 (2C), 126.2, 128.4, 130.2, 132.0, 133.6, 137.3, 138.0, and 161.4. MS *m*/*z*: 316 (M^+^). HRMS (EI): calcd for C_20_H_16_N_2_O_2_ 316.1212; found 316.1208.

#### 3.1.30. Naucleficine (**7**)

A suspension of alcohol **34** (30 mg, 0.095 mmol) and active MnO_2_ (165 mg, 1.9 mmol) in CH_2_Cl_2_ (2 mL) was stirred at reflux for 1 h. The reaction mixture was filtered through a Celite pad. The filtrate was evaporated in vacuo. The residue was purified by column chromatography (EtOAc/hexane 1:1, *v*/*v*) to give naucleficine (**7**; 21 mg, 70%) as an orange solid. mp was 276–277 °C (CHCl_3_ lit. [30] mp of 284–290 °C). IR (ATR) ν = 1592, 1643, and 3221 cm^−1^. ^1^H-NMR (400 MHz, DMSO-*d*_6_) δ 3.11 (t, *J* = 6.6 Hz, 2H), 4.41 (t, *J* = 6.6 Hz, 2H), 7.08 (t, *J* = 7.8 Hz, 1H), 7.24 (t, *J* = 7.8 Hz, 1H), 7.47 (d, *J* = 7.8 Hz, 1H), 7.59–7.64 (m, 2H), 8.12 (s, 1H), 8.25 (d, *J* = 7.8 Hz, 1H), 8.53 (d, *J* = 7.8 Hz, 1H), 10.42 (s, 1H), 11.82 (br s, 1H). ^13^C-NMR (100 MHz, DMSO-*d*_6_) δ 19.2, 40.5, 94.9, 112.0, 114.1, 119.4, 119.7, 124.1, 125.4, 125.5 (2C), 127.9, 129.6, 133.8, 135.1, 135.6, 138.5, 138.6, 160.8, and 192.8. MS *m*/*z*: 314 (M^+^). HRMS (EI): calcd for C_20_H_14_N_2_O_2_ 314.1055; found 314.1066.

### 3.2. Biochemistry

#### 3.2.1. Cell Lines and Cell Cultures

For testing the antiproliferative cell activities, two types of cancer cell lines were used in this study: HCT-116 cells (human colon cancer) and HepG2 cells (human liver cancer), which were purchased from the American Type Culture Collection (Manassas, VA, USA) and the Japanese Collection of Research Bioresources (Osaka, Japan). The HCT-116 and HepG2 cells were maintained in a McCOY’s 5A medium (Sigma-Aldrich, MO, USA) with L-glutamine and 10% heat inactivated (55 °C for 30 min) fetal bovine serum (FBS) and in a Dulbecco’s Modified Eagle’s medium (FUJIFILM Wako Pure Chemical Corporation, Osaka, Japan) with L-glutamine and 10% heat-inactivated FBS, respectively, at 37 °C in an atmosphere of 5% CO_2_.

#### 3.2.2. Cell Viability Assays

The HCT-116 cells’ viability assay was conducted using the MTT method based on the procedure described by Mosmann [36]. Briefly, cells were placed in 96-well flat-bottomed tissue culture plates with 2.0 × 10^3^ cells per well in a 100 μL culture medium. This was followed by incubation at 37 °C in an atmosphere of 5% CO_2_ for 24 h to allow the cells to attach to the wells. The cells were treated with the indicated concentrations of test agents in a culture medium without FBS. Following a further 72 h incubation, 10 μL of MTT (5 mg/mL in phosphate-buffered saline) was added per well, and the plate was incubated for 4 h to allow the MTT to metabolize by cellular mitochondrial dehydrogenases. The excess MTT was aspirated, and the produced formazan crystals were dissolved by adding 100 μL of dimethyl sulfoxide. The absorbance of the purple formazan was read at 570 nm using a microplate reader. The results following the test agents’ exposure were calculated as a percentage relative to untreated controls. The HepG2 cells’ viability assay was conducted using the WST-8 method based on the procedure described by Tominaga [37]. The cells were seeded in 96-well flat-bottomed tissue culture plates with 1.0 × 10^4^ cells per well in 100 µL of the FBS-containing culture medium with the indicated concentrations of test agents. Following a further 72 h incubation, 10 µL of a mixture of WST-8 solution was added per well, and the plate was incubated for 2 h to allow the WST-8/1-methoxy PMS to metabolize by cellular mitochondrial dehydrogenases. The absorbance of the orange formazan was read at 450 nm using a microplate reader. The results following the test agents’ exposure were calculated as a percentage relative to untreated controls.

#### 3.2.3. Statistical Calculation

The concentration cells’ viability curves were fitted to a four-parametric logistic equation using a nonlinear curve-fitting program that derived the IC_50_ values (Kaleida-graph ver. 3.6; Synergy Software, PA, USA). Wherever appropriate, the results were expressed as means ± sem, with n ¼ 3 or higher in at least one out of three similar experiments.

## 4. Conclusions

We developed a versatile synthetic methodology for introducing various substituents into the E-ring of the pentacyclic aromathecin scaffold, which allowed us to achieve the total synthesis of 22-hydroxyacuminatine (**4**) as a model compound of the aromathecin family.

The pentacyclic scaffold was obtained by constructing the indolizine moiety from isoquinolone **17** by C–N bond formation via the Mitsunobu reaction. Isoquinolone **17** was synthesized via the thermal cyclization of 2-alkynylbenzaldehyde oxime to obtain *N*-oxide **16**, which was then subjected to the Reissert–Henze-type reaction. Consequently, **4** was obtained in 10.5% overall yield via an eight-step sequence. Using this methodology, we achieved the total synthesis of the indoloquinolizidine-type alkaloids norketoyobyrine (**5**: 10 steps, total yield 17.2%) and naucleficine (**7**: 11 steps, total yield 13.3%). Further, we assessed the antiproliferative activity of the synthesized **5**, **7**, and intermediate **34** against HCT-116 human colon cancer cells and HepG2 human liver cancer cells, concluding that **7** and **34** exhibited moderate antiproliferative activity against HCT-116 cells with IC_50_ values of 55.58 and 41.40 μM, respectively. Future research will focus on the synthesis of diverse pentacyclic compounds with promising therapeutic profiles by adopting this strategy.

## Figures and Tables

**Table 1:** Investigation of optimal isoquinoline *N*-oxides for synthesis of isoquinolones by Reissert–Henze-type reaction.

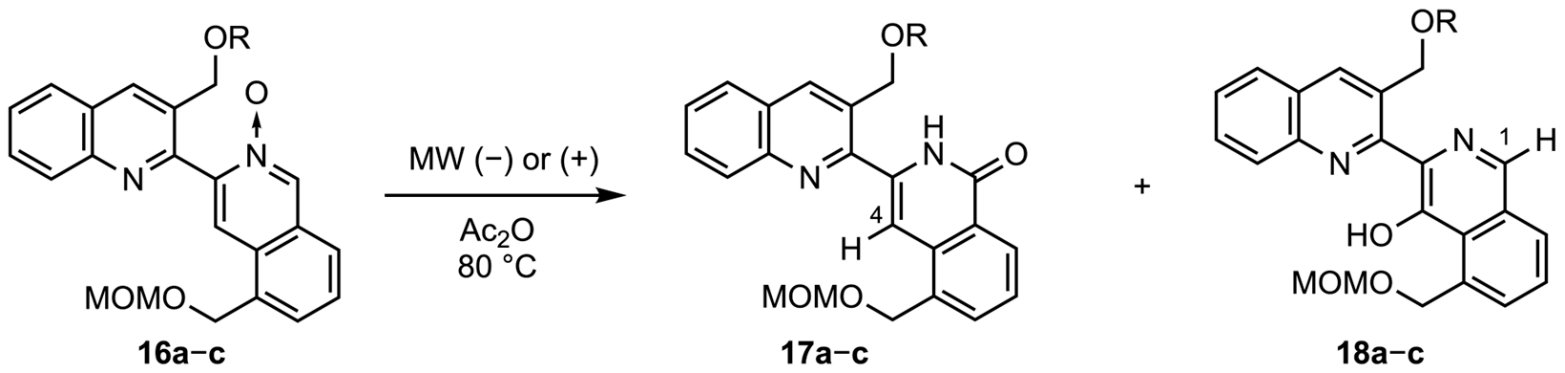						
Entry	R	Compd. No.	Time (h) ^(1)^	MW	Yield (%)	
					Isoquinolone 17 ^(2)^	4-OH-isoquinoline 18 ^(3)^
1	H	**a**	12	−	trace ^(4)^	trace ^(4)^
2	H	**a**	5	+	trace ^(4)^	trace ^(4)^
3	TBS	**b**	24	−	20	−
4	TBS	**b**	2	+	36	−
5	Ac	**c**	6	−	20	27
6	Ac	**c**	5	+	50	37

(1) Reissert–Henze-type reaction is carried out while monitoring the disappearance of *N*-oxides **16** by TLC. (2) Isoquinolones **17** are identified by observing the chemical shift in their 4-H at approximately 7.43 ppm as a singlet peak in ^1^H-NMR measurement. (3) 4-OH-isoquinolines **18** are identified by observing the chemical shift in their 1-H at approximately 9.36 ppm as a singlet peak in ^1^H-NMR measurement. (4) **17a** and **18a** are not obtained, and only small amounts of **17c** and **18c**, which are acetylated hydroxymethyl groups of **17a** and **18a**, are isolated.

**Table 2:** A summary on total synthesis of 22-hydroxyacuminatine (**4**), norketoyobyrine (**5**), and naucleficine (**7**).

Natural Product	Year	Authors	Steps	Total Yield (%)	Reference
22-hydroxyacuminatine		Our group	8	10.5	
	2004	Lee	7	14.9	[6]
	2006, 2008	Kanazawa	7	21.9	[7,10]
	2006	Cushman	9	7.4	[8]
	2007	Yao	9	21.1	[9]
	2016	Gao	10	9.3	[11]
norketoyobyrine		Our group	10	17.2	
	1949	Swan	5	12.6	[13]
	1973, 1976	Ninomiya	2	31.0	[14,15]
	1986, 1990	Grigg	2	19.2	[16,17]
	1992	Yamaguchi	3	54.1	[18]
	2000	Sánchez-Sancho	5	38.1	[19]
	2000	Knölker	8	25.5	[20]
	2014	Bannister	4	45.1	[21]
	2016	Gao	12	29.9	[11]
	2020	Cai and Luo	4	46.5	[22]
	2021	Christman	2	5.7	[23]
	2021	Zhu and Song	2	55.2	[24]
naucleficine		Our group	11	13.3	
	1986, 1991	Naito	5	23.0	[29,30]
	2016	Gao	10	20.2	[11]

**Table 3:** IC_50_ values of synthetic products on antiproliferative activities against HCT-116 and HepG2 cells.

Compd. No.	IC_50_ Value (μM)	
	HCT-116 Cells	HepG2 Cells
**5**	>100	>100
**7**	55.58 ± 3.79	>100
**34**	41.40 ± 1.28	>100

## Data Availability

Data are contained within the article and Supplementary Materials.

## Supplementary Materials

The following supporting information can be downloaded at https://www.mdpi.com/article/10.3390/molecules30122650/s1, copies of the ^1^H NMR and ^13^C NMR spectra for compounds **14**–**34**, 22-hydroxyacuminatine (**4**), norketoyobyrine (**5**), and naucleficine (**7**).

## Abbreviations

The following abbreviations are used in this manuscript:

TBS	*tert*-butyldimethylsilyl
Ac	acetyl
1,2-DCB	1,2-dichlorobenzene
MW	microwave
TLC	thin-layer chromatography
DIAD	diisopropyl azodicarboxylate
MOM	methoxymethyl
TBAF	tetrabutylammonium fluoride
