# Supplementary material for: Total Synthesis and Biological Evaluation of 22-Hydroxyacuminatine and the Related Natural Products Norketoyobyrine and Naucleficine
Source: Molecules. 2025 Jun 19;30(12):2650. doi: 10.3390/molecules30122650 (PMC12195643; doi:10.3390/molecules30122650)
Supplement: Supplementary file 1 [file molecules-30-02650-s001.zip › molecules-3692873-supplementary.pdf]

Total Synthesis and Biological Evaluation of 22-Hydroxyacuminatine and the Related Natural Products Norketoyobyrine and Naucleficine

Shohta Mizuno [1,†], Takashi Nishiyama [1], Hana Bessho [1], Tetsuya Nakamura [1,†], Tomoki Oe [1], Nanako Hayashi [1], Yuhzo Hieda [1], Toshio Motoyashiki [1], Toshiyuki Hata [1], Noriyuki Hatae [2,*] and Tominari Choshi [1,*]

1 Faculty of Pharmacy and Pharmaceutical Sciences, Fukuyama University, 1 Sanzo, Gakuen-cho, Fukuyama 729-0292, Hiroshima, Japan; s.mizuno@fukuyama-u.ac.jp (S.M.); t_nishiyama@fukuyama-u.ac.jp (T.N.); p7120085@fukuyama-u.ac.jp (H.B.); t-nakamura@fukuyama-u.ac.jp (T.N.); p7121016@fukuyama-u.ac.jp (T.O.); p7121072@fukuyama-u.ac.jp (N.H.); hieda@fukuyama-u.ac.jp (Y.H.); motoyashiki@fukuyama-u.ac.jp (T.M.); hata@fukuyama-u.ac.jp (T.H.)

2 Faculty of Pharmaceutical Sciences, Yokohama University of Pharmacy, 601 Matano, Totsuka-ku, Yokohama 245-0066, Kanagawa, Japan

* Correspondence: noriyuki.hatae@hamayaku.ac.jp (N.H.); choshi@fukuyama-u.ac.jp (T.C.); Tel.: +81-45-859-1300 (N.H.); +84-936-2111 (T.C.); Fax: +81-45-859-1301 (N.H.); +81-84-936-2024 (T.C.)

† These authors contributed equally to this work.

Supporting Information

Copies of ^{1}H NMR and ^{13}C NMR Spectra of Synthesized Compounds

3.1.1. *2-(3-Hydroxymethylquinolin-2-yl)ethynyl-3-[(methoxymethoxy)methyl]benzaldehyde* (**14a**)

3.1.2. *2-{[3-(tert-Butyldimethylsilyloxy)methyl]quinolin-2-yl}ethynyl-3-[(methoxymethoxy)methyl]benzaldehyde* (**14b**)

3.1.3. *2-[3-(Acetoxymethyl)quinolin-2-yl]ethynyl-3-[(methoxymethoxy)methyl]benzaldehyde* (**14c**)

3.1.4. *2-(3-Hydroxymethylquinolin-2-yl)ethynyl-3-[(methoxymethoxy)methyl]benzaldehyde oxime* (**15a**)

3.1.5. *2-[3-(Acetoxymethyl)quinolin-2-yl]ethynyl-3-[(methoxymethoxy)methyl]benzaldehyde oxime* (**15c**)

3.1.6. *3-(3-Hydroxymethylquinolin-2-yl)-5-[(methoxymethoxy)methyl]isoquinoline N-oxide* (**16a**)

3.1.7. *2-{3-[(tert-Butyldimethylsilyloxy)methyl]quinolin-2-yl}-5-[(methoxymethoxy)methyl]isoquinoline N-oxide* (**16b**)

3.1.8. *2-(3-Acetoxymethylquinolin-2-yl)-5-[(methoxymethoxy)methyl]isoquinoline N-oxide* (**16c**)

3.1.9. *3-{3-[(tert-Butyldimethylsilyloxy)methyl]quinolin-2-yl}-5-[(methoxymethoxy)methyl]isoquinolin-1-one* (**17b**)

3.1.10. *3-(3-Acetoxymethylquinolin-2-yl)-5-[(methoxymethoxy)methyl]isoquinolin-1-one* (**17c**)
3-[3-(Acetoxymethyl)quinolin-2-yl]-4-hydroxy-3-[(methoxymethoxy)methyl]isoquinoline (**18c**)

3.1.11. *3-(3-Hydroxymethylquinolin-2-yl)-5-[(methoxymethoxy)methyl]isoquinolin-1-one* (**19**)

3.1.12. *22-Hydroxyacuminatine* (**4**)

3.1.13. *Methyl [2-iodo-N-(methoxymethyl)indol-3-yl]acetate* (**22**)

3.1.14. *Methyl [2-trimethylsilylethynyl-N-(methoxymethyl)lindol-3-yl]acetate* (**23**)

3.1.15. *Methyl [2-ethynyl-N-(methoxymethyl)indol-3-yl]acetate* (**24**)

3.1.16. *Methyl {2-[2-(2-formylphenyl)ethynyl]-N-(methoxymethyl)indol-3-yl}acetate* (**26a**)

3.1.17. *Methyl {2-[2-(2-formyl-3-[(methoxymethoxy)methyl]phenyl)ethynyl]-N-(methoxymethylindol-3-yl}acetate* (**26b**)

3.1.18. *Methyl {2-[22-hydroxyiminophenyl)ethynyl]-N-(methoxymethyl)indol-3-yl}acetate* (**27a**)

3.1.19. *Methyl {2-[2-(2-hydroxyimino-3-[(methoxymethoxy)methyl]phenyl)ethynyl]-N-methoxymethylindol-3-yl}acetate* (**27b**)

3.1.20. *3-[3-(2-Methoxy-2-oxoethyl)-1-(methoxymethyl)indol-2-yl]isoquinoline N-oxide* (**28a**)

3.1.21. *3-[3-(2-Methoxy-2-oxoethyl)-1-(methoxymethyl)indol-2-yl)-5-[(methoxymethoxy)methyl]isoquinoline N-oxide* (**28b**)

3.1.22. *Methyl 2-[1-(methoxymethyl)-2-(1-oxo-1,2-dihydroisoquinolin-3-yl)indol-3-yl]acetate* (**29a**)

Methyl 2-[2-(4-acetoxyisoquinolin-3-yl)-1-(methoxymethyl)indol-3-yl]acetate (**31a**)

3.1.23. *Methyl 2-{2-[5-[(methoxymethoxy)methyl]-1-oxo-1,2-dihydroisoquinolin-3-yl]-1-(methoxymethyl)indol-3-yl}acetate* (**29b**)

1-Acetoxy-3-[3-methoxycarbonylmethyl-N-(methoxymethyl)indol-2-yl]-5-[(methoxymethoxy)methyl]isoquinoline (**30b**)

4-Acetoxy-3-[3-methoxycarbonylmethyl-N-(methoxymethyl)indol-2-yl]-5-[(methoxymethoxy)methyl]isoquinoline (**31b**)

3.1.24. *3-[3-(2-Hydroxyethyl)-N-(methoxymethyl)indol-2-yl]isoquinolin-1-one* (**32a**)

3.1.25. *3-[3-(2-Hydroxyethyl)-N-(methoxymethyl)indol-2-yl]-5-[(methoxymethoxy)methyl]isoquinolin-1-one* (**32b**)

3.1.26. *N-Methoxymethyl-3,14,15,16,17,18,19,20-octadehydroyohimban-21-one* (**33a**)

3.1.27. *N-Methoxymethyl-16-[(methoxymethoxy)methyl]-3,14,15,16,17,18,19,20-octadehydroyohimban-21-one* (**33b**)

3.1.28. *Norketoyobyrine* (**5**)

3.1.29. *16-Hydroxymethyl-3,14,15,16,17,18,19,20-octadehydroyohimban-21-one* (**34**)

3.1.30. *Naucleficine* (**7**)

3.1.1 *2-(3-Hydroxymethylquinolin-2-yl)ethynyl-3-[(methoxymethoxy)methyl]benzaldehyde* (**14a**)

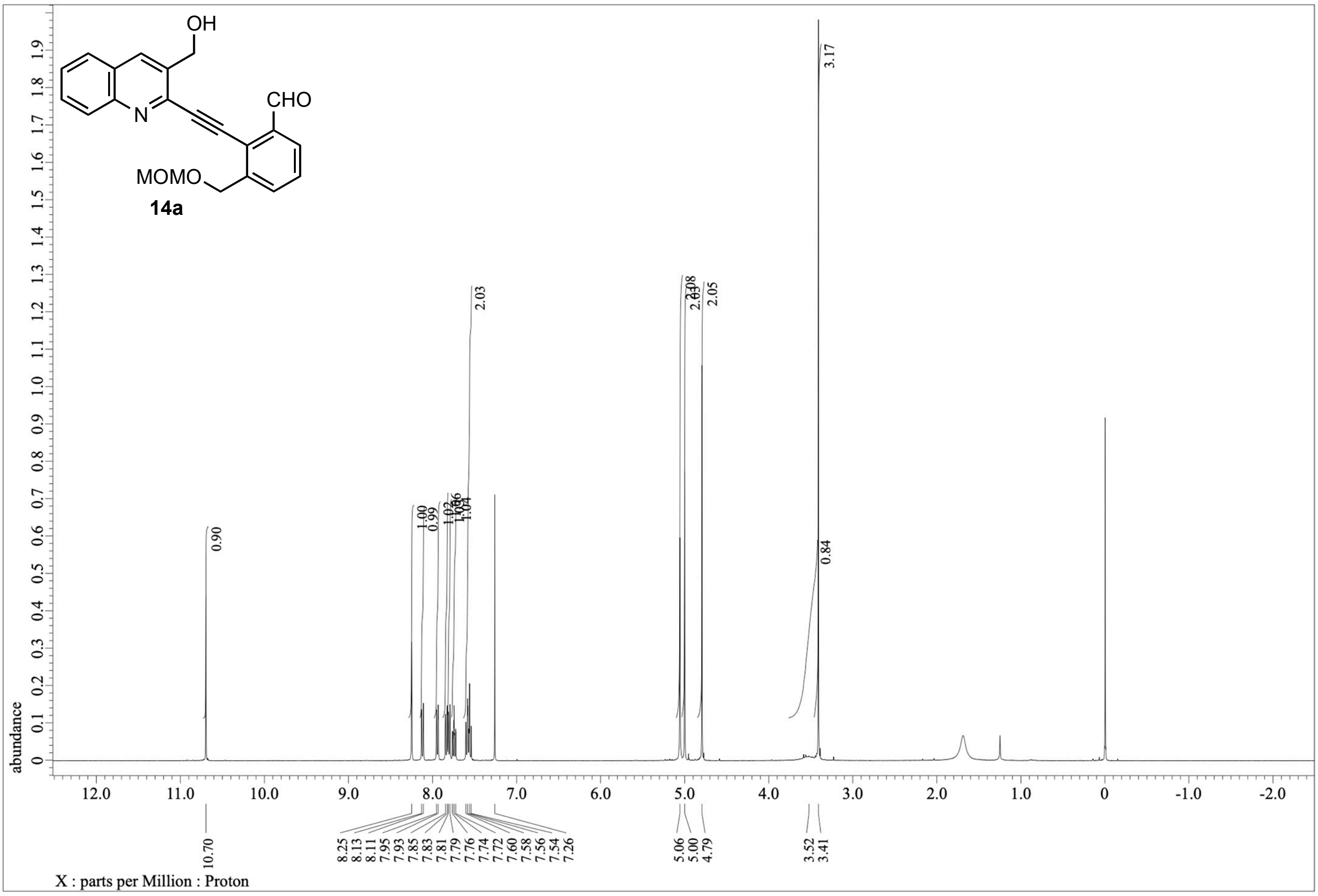

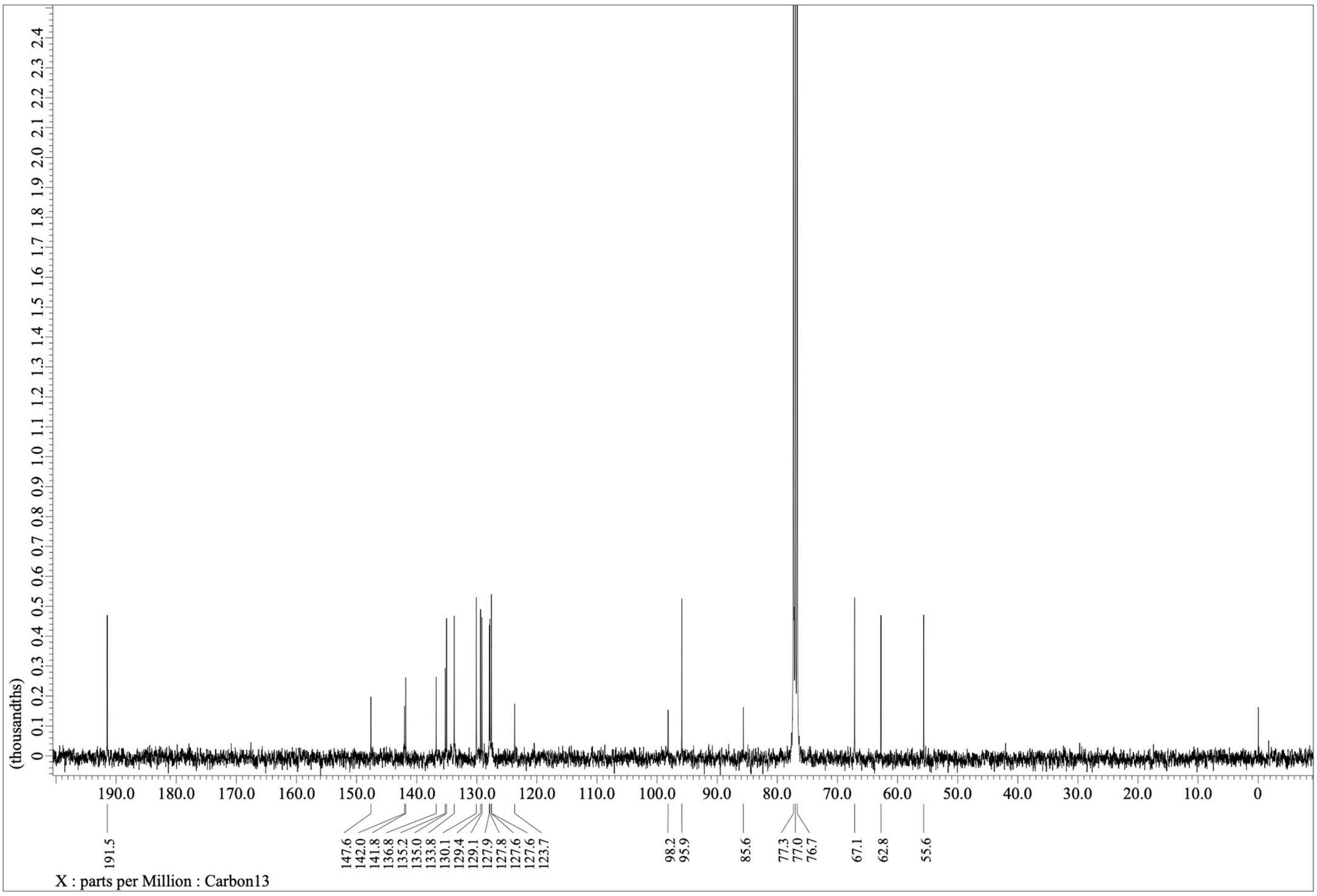

3.1.2. *2-{[3-(tert-Butyldimethylsilyloxy)methyl]quinolin-2-yl}ethynyl-3-[(methoxymethoxy)methyl]benzaldehyde* (**14b**)

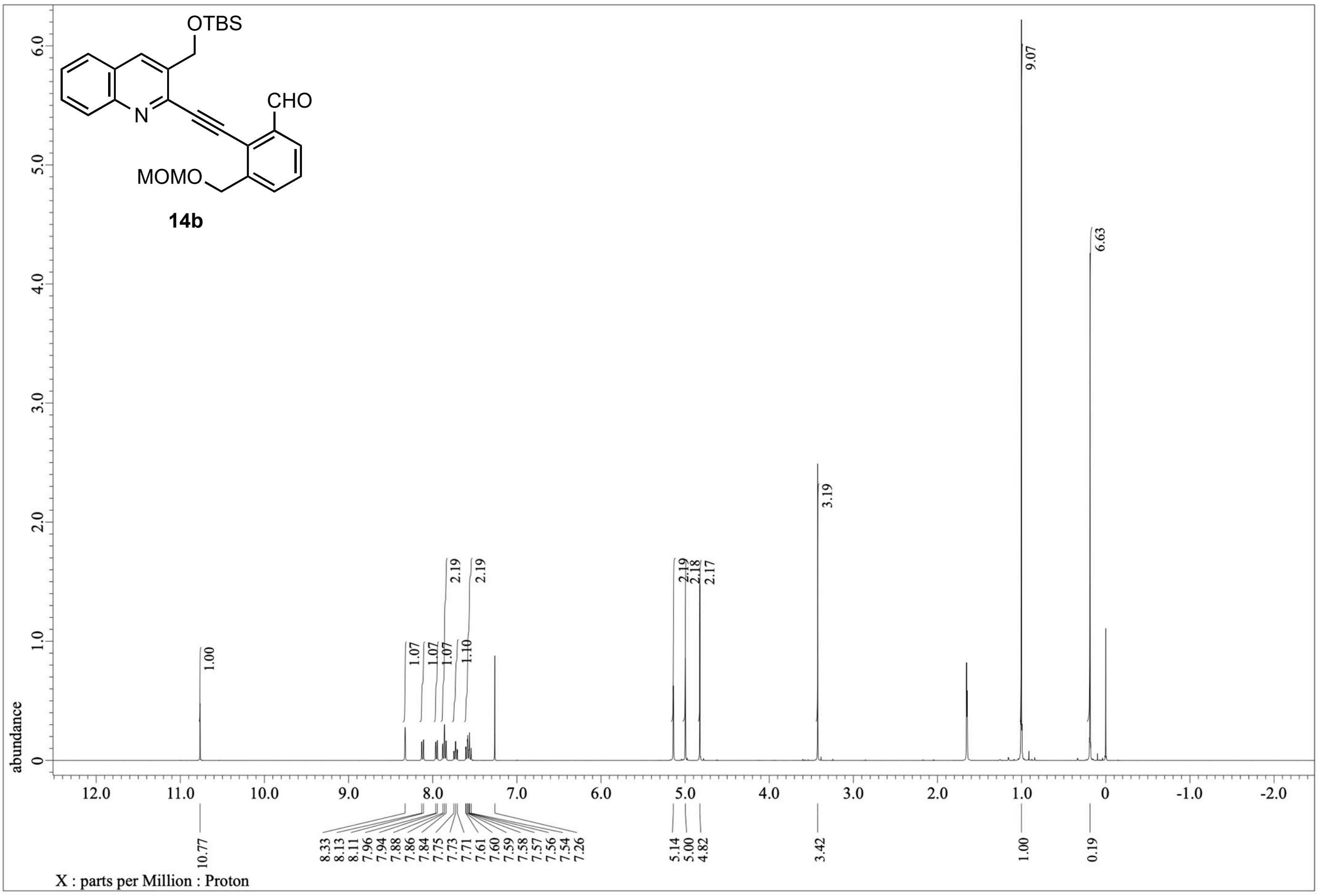

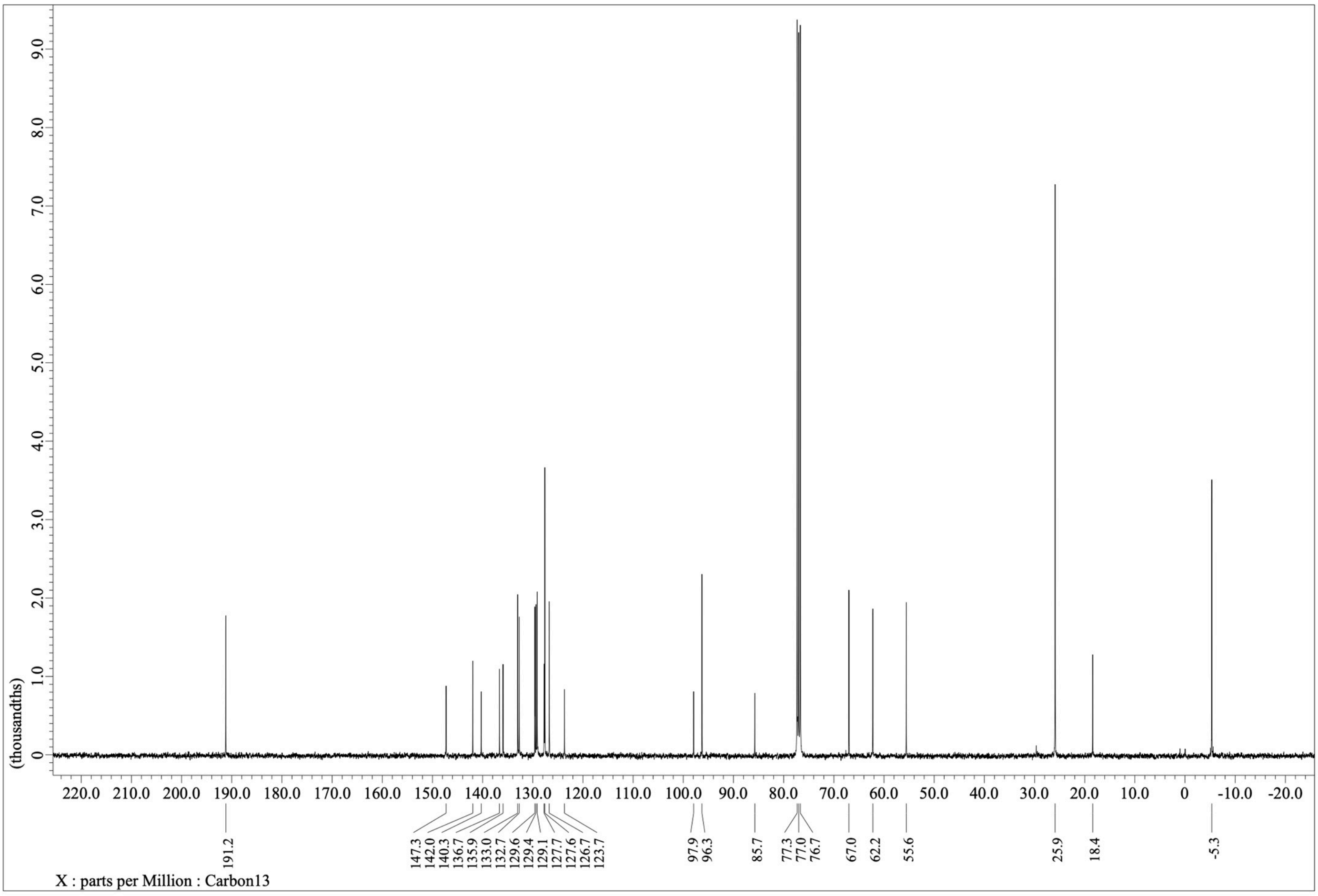

3.1.3. *2-[3-(Acetoxymethyl)quinolin-2-yl]ethynyl-3-[(methoxymethoxy)methyl]benzaldehyde* (**14c**)

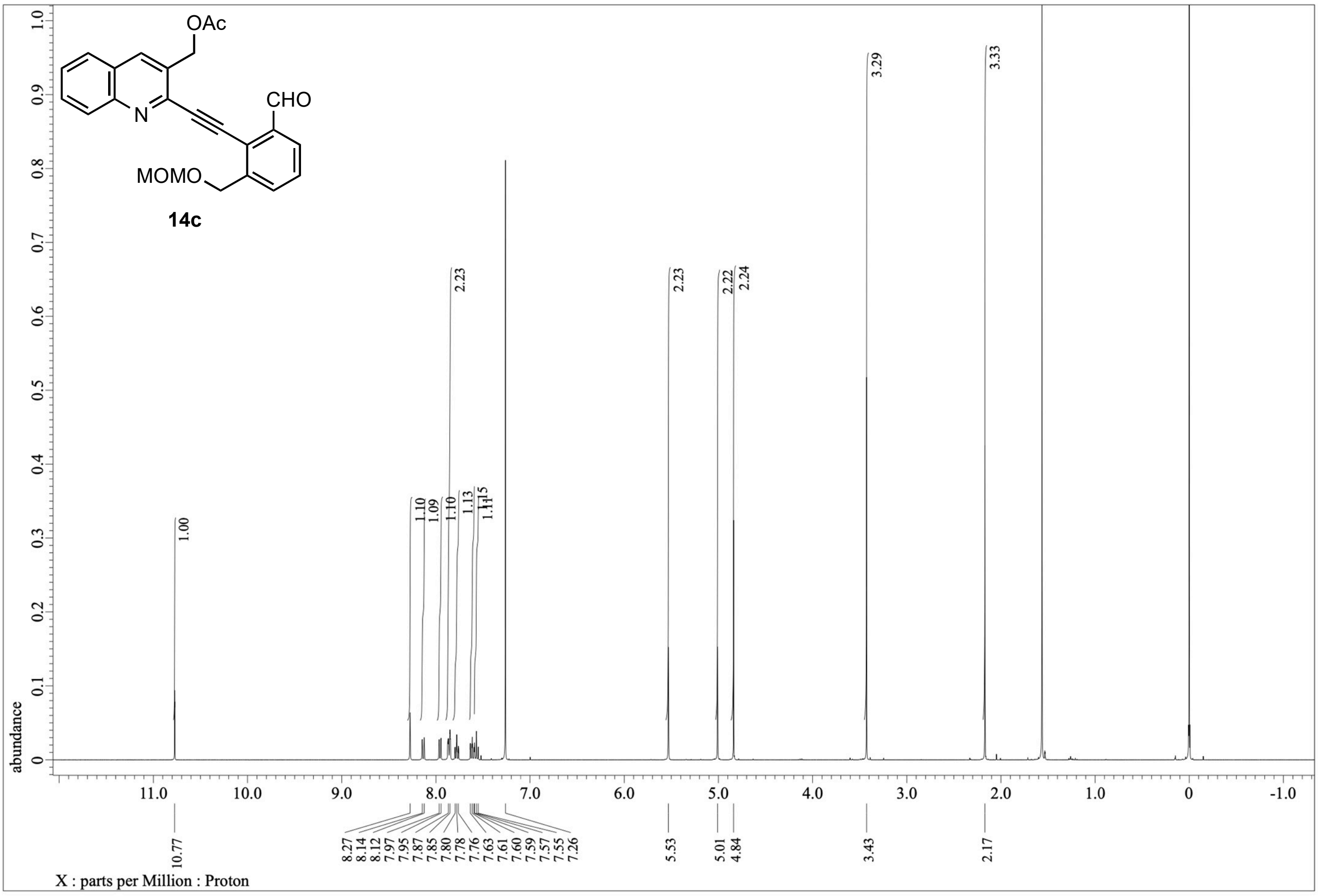

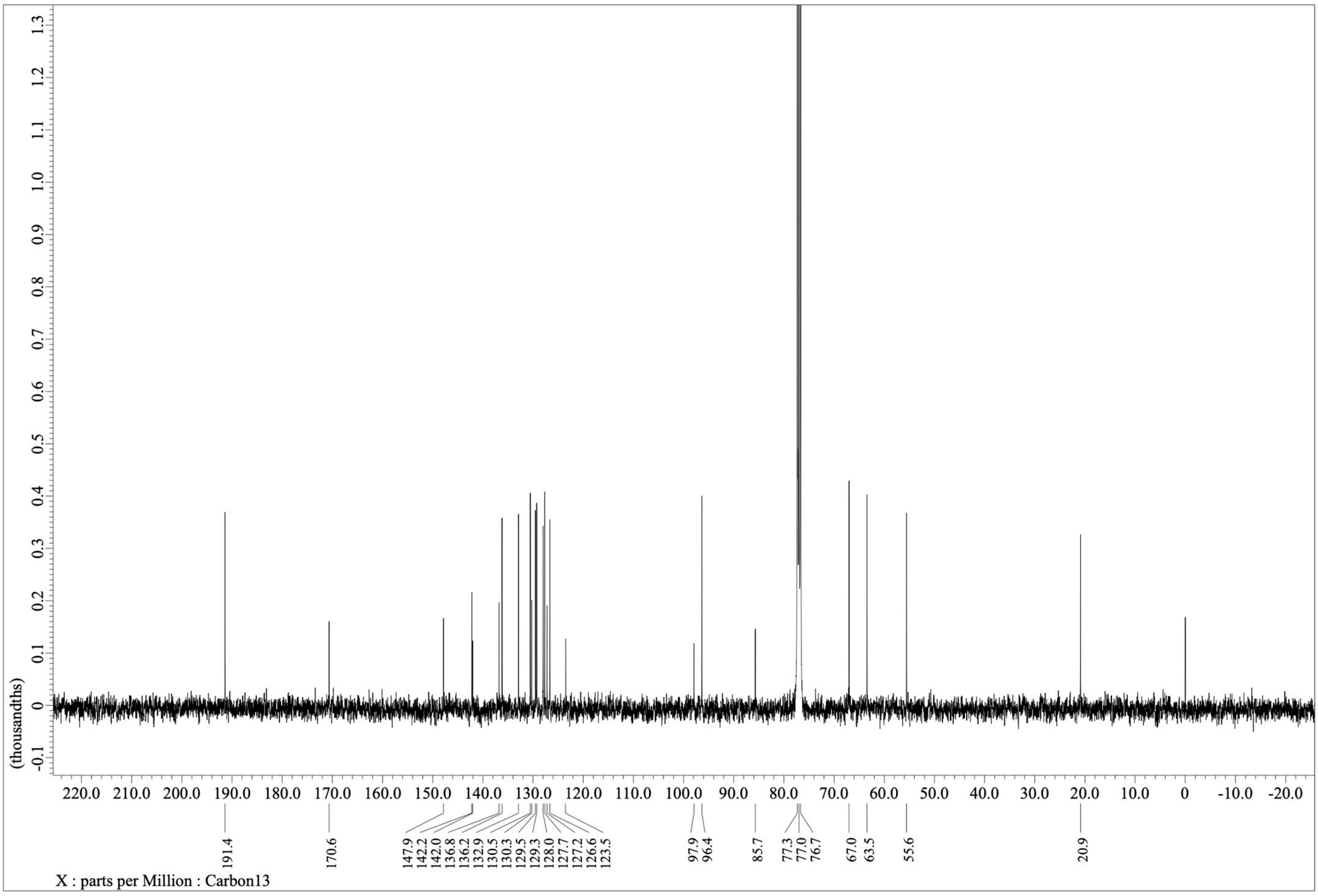

3.1.4. *2-(3-Hydroxymethylquinolin-2-yl)ethynyl-3-[(methoxymethoxy)methyl]benzaldehyde oxime* (**15a**)

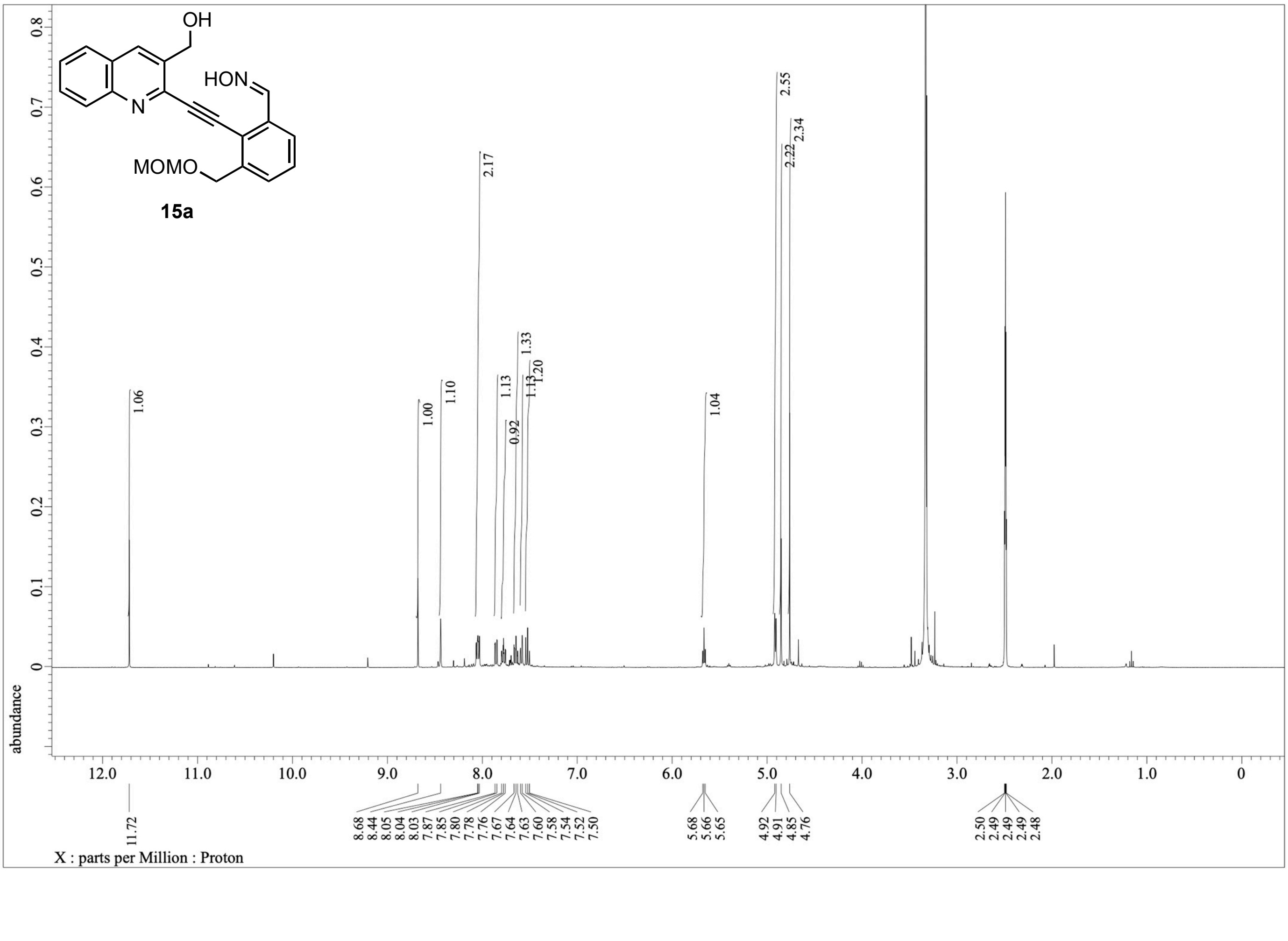

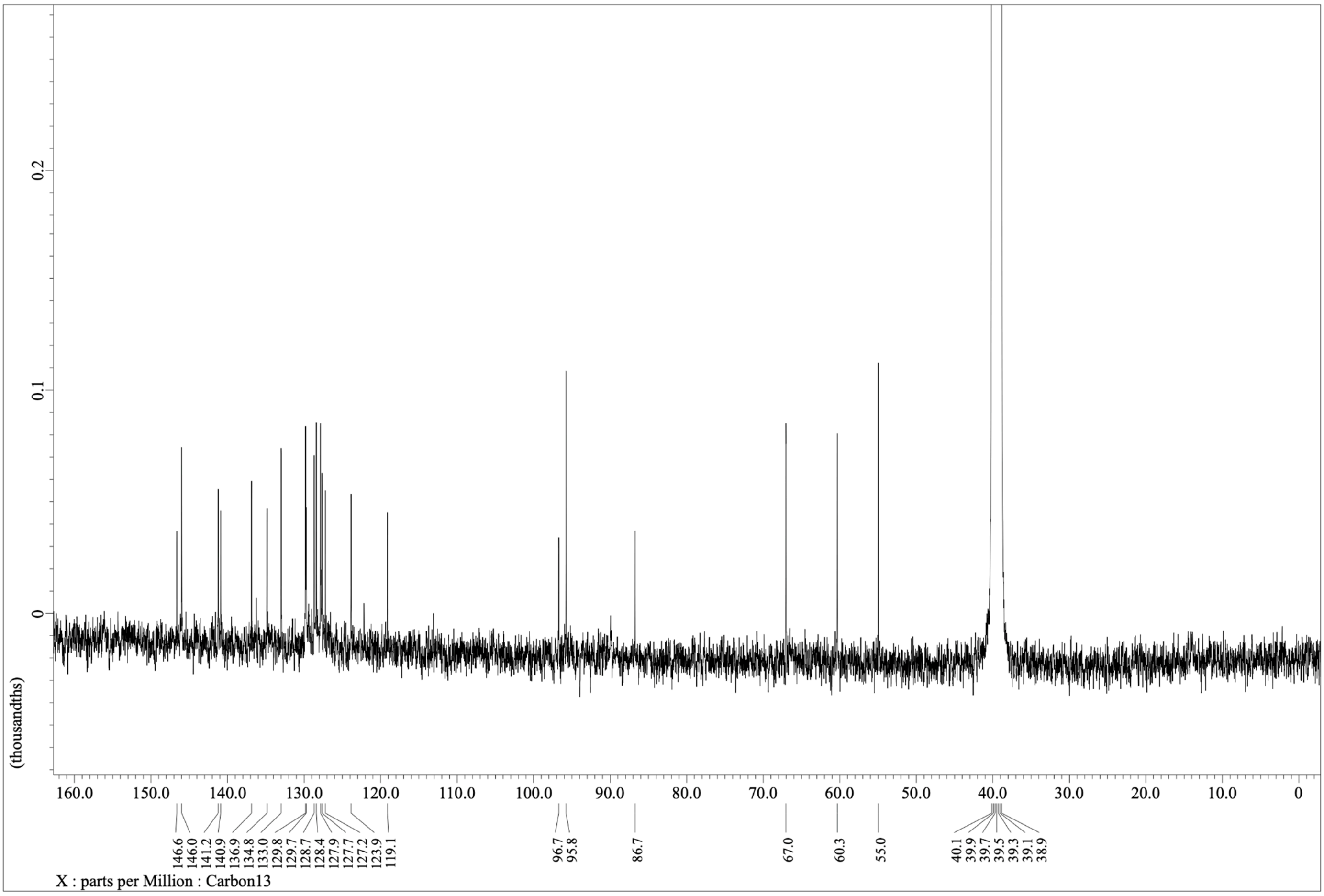

3.1.5. *2-[3-(Acetoxymethyl)quinolin-2-yl]ethynyl-3-[(methoxymethoxy)methyl]benzaldehyde oxime* (**15c**)

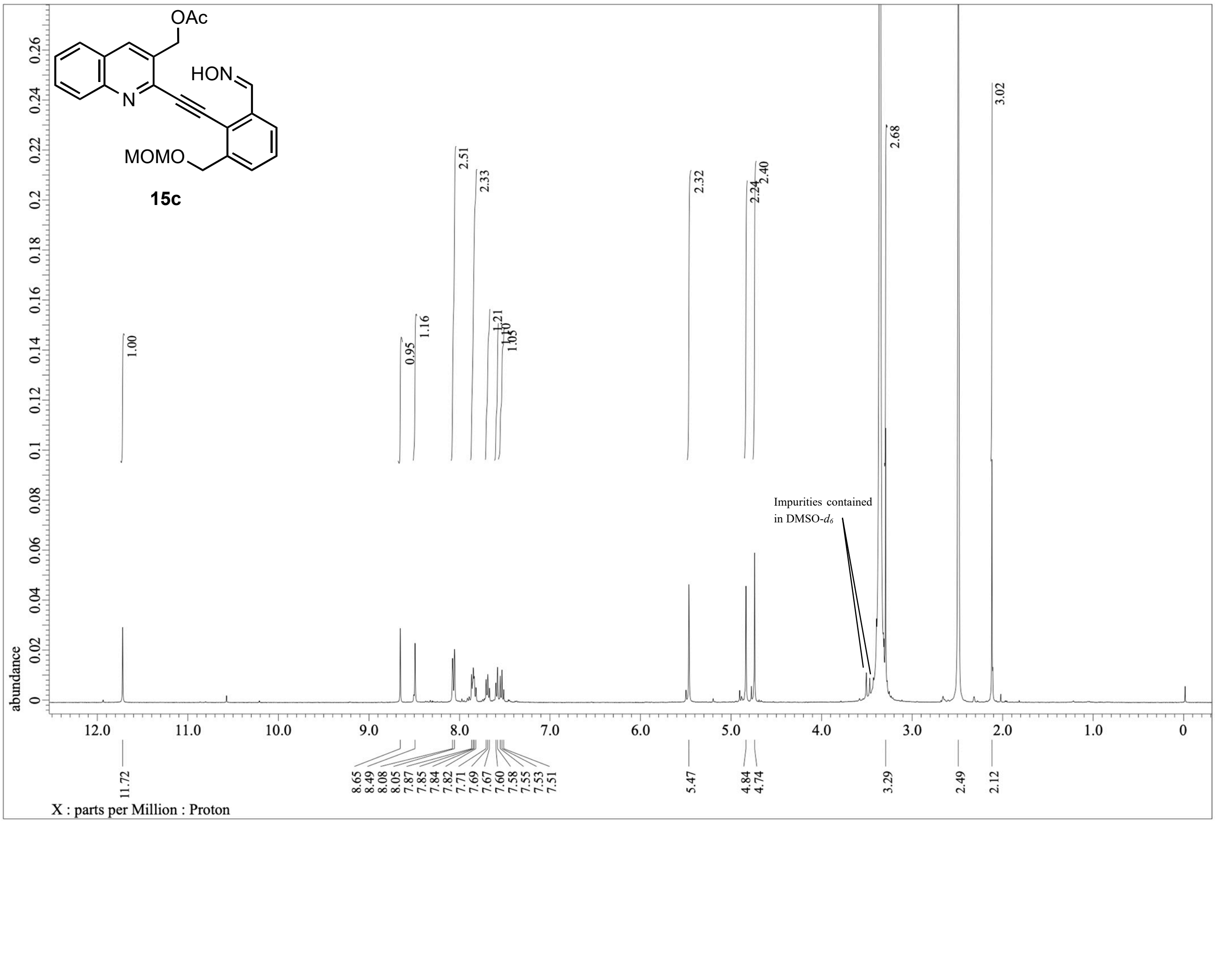

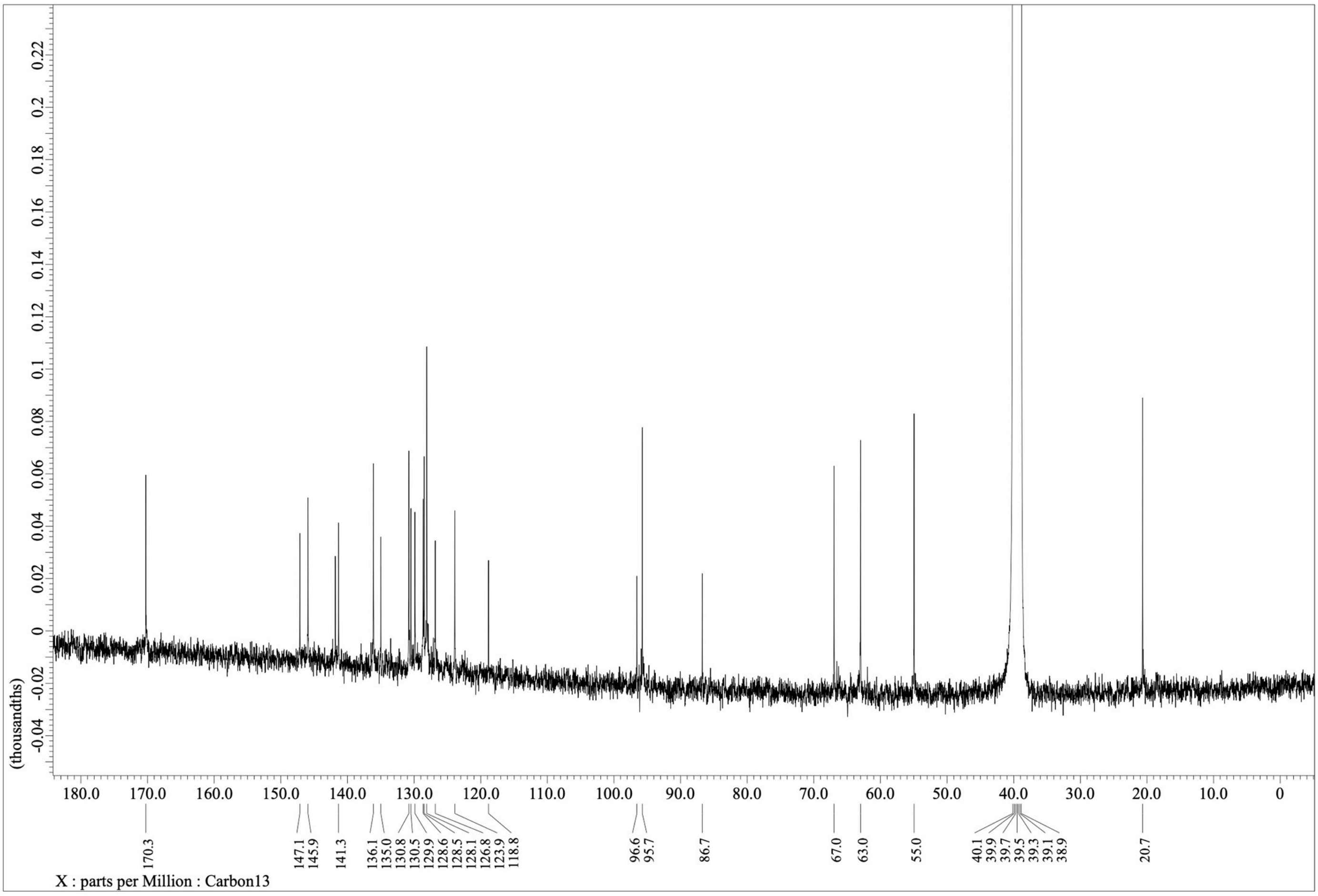

3.1.6. *3-(3-Hydroxymethylquinolin-2-yl)-5-[(methoxymethoxy)methyl]isoquinoline N-oxide* (**16a**)

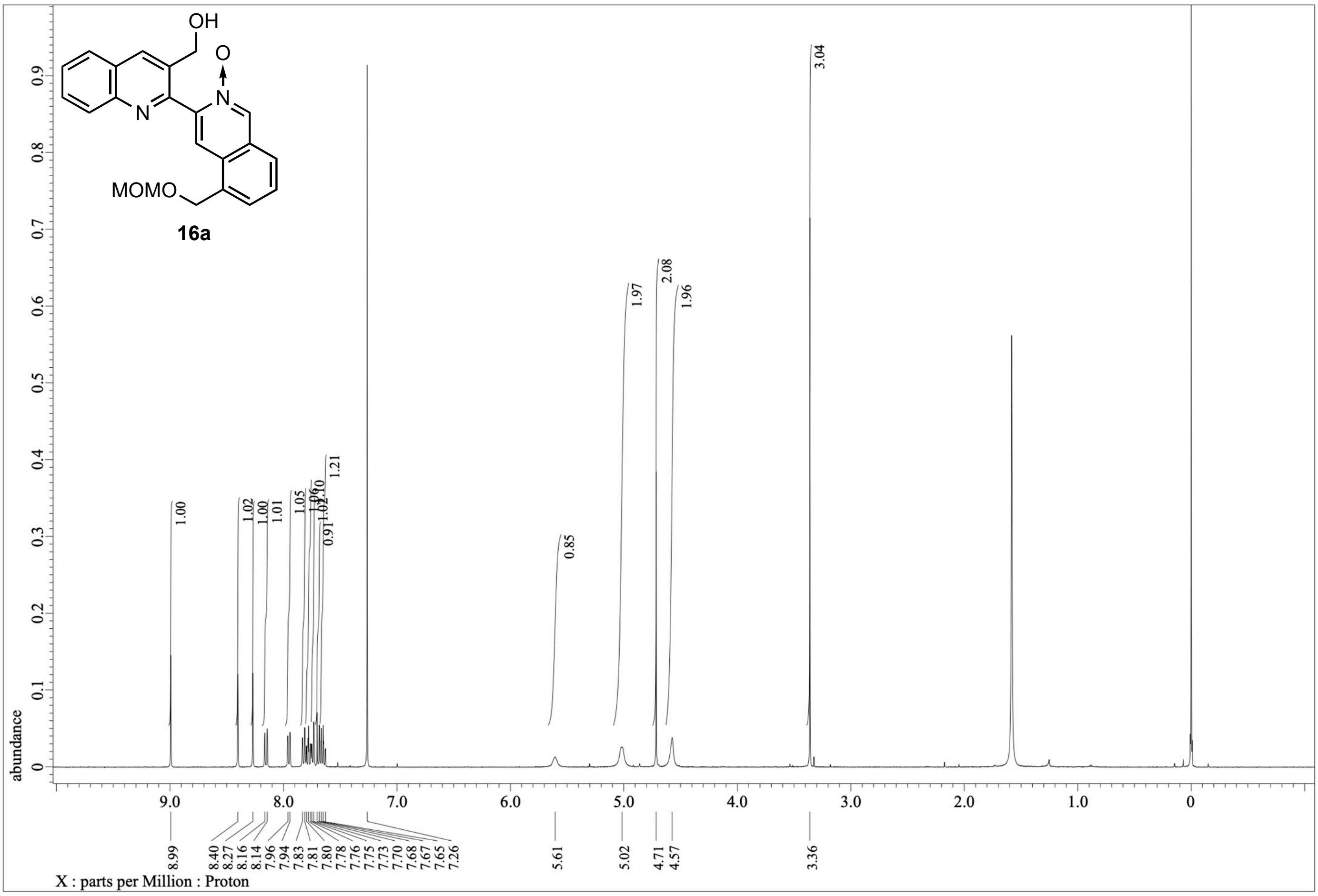

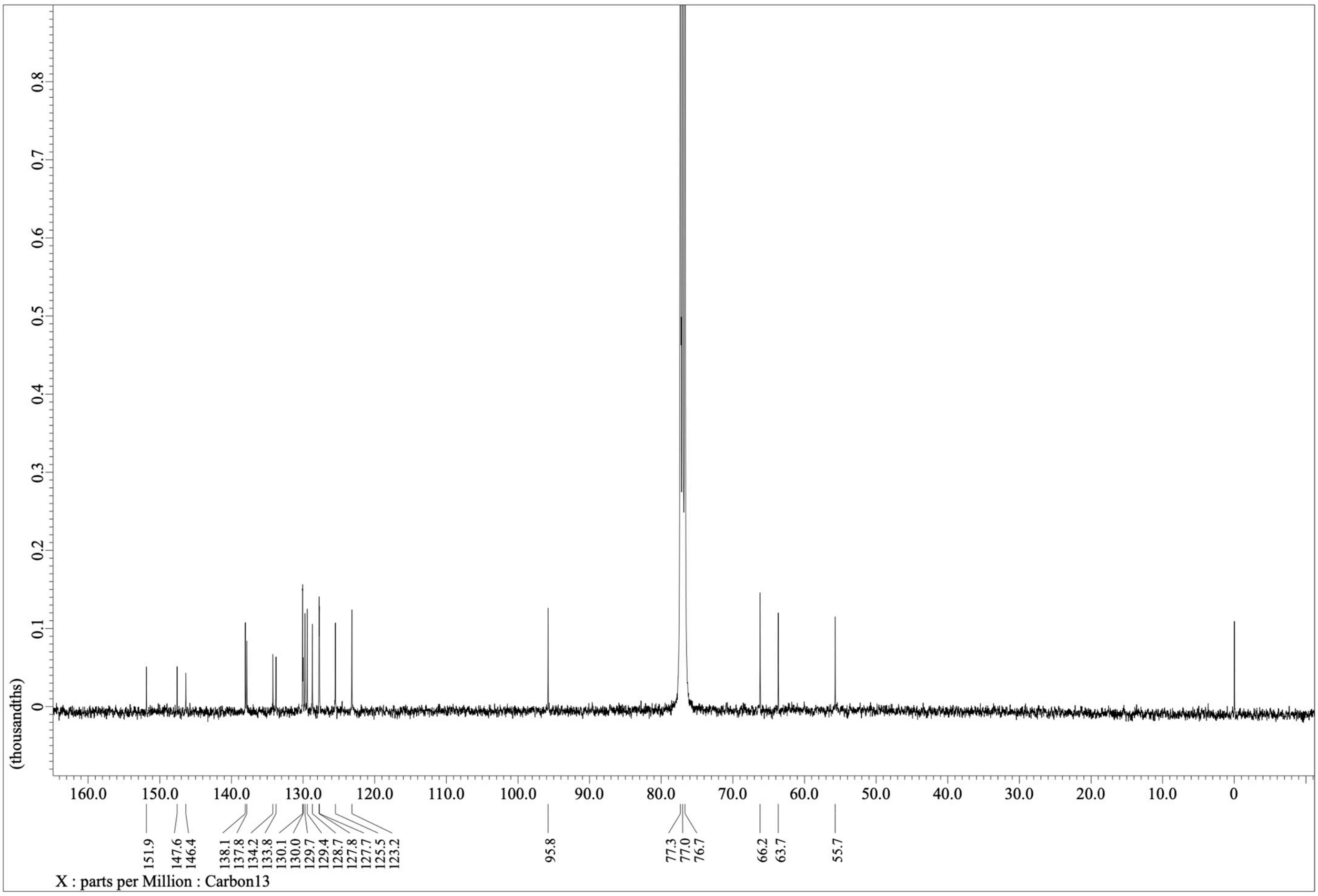

3.1.7. *2-{3-[(tert-Butyldimethylsilyloxy)methyl]quinolin-2-yl}-5-[(methoxymethoxy)methyl]isoquinoline N-oxide* (**16b**)

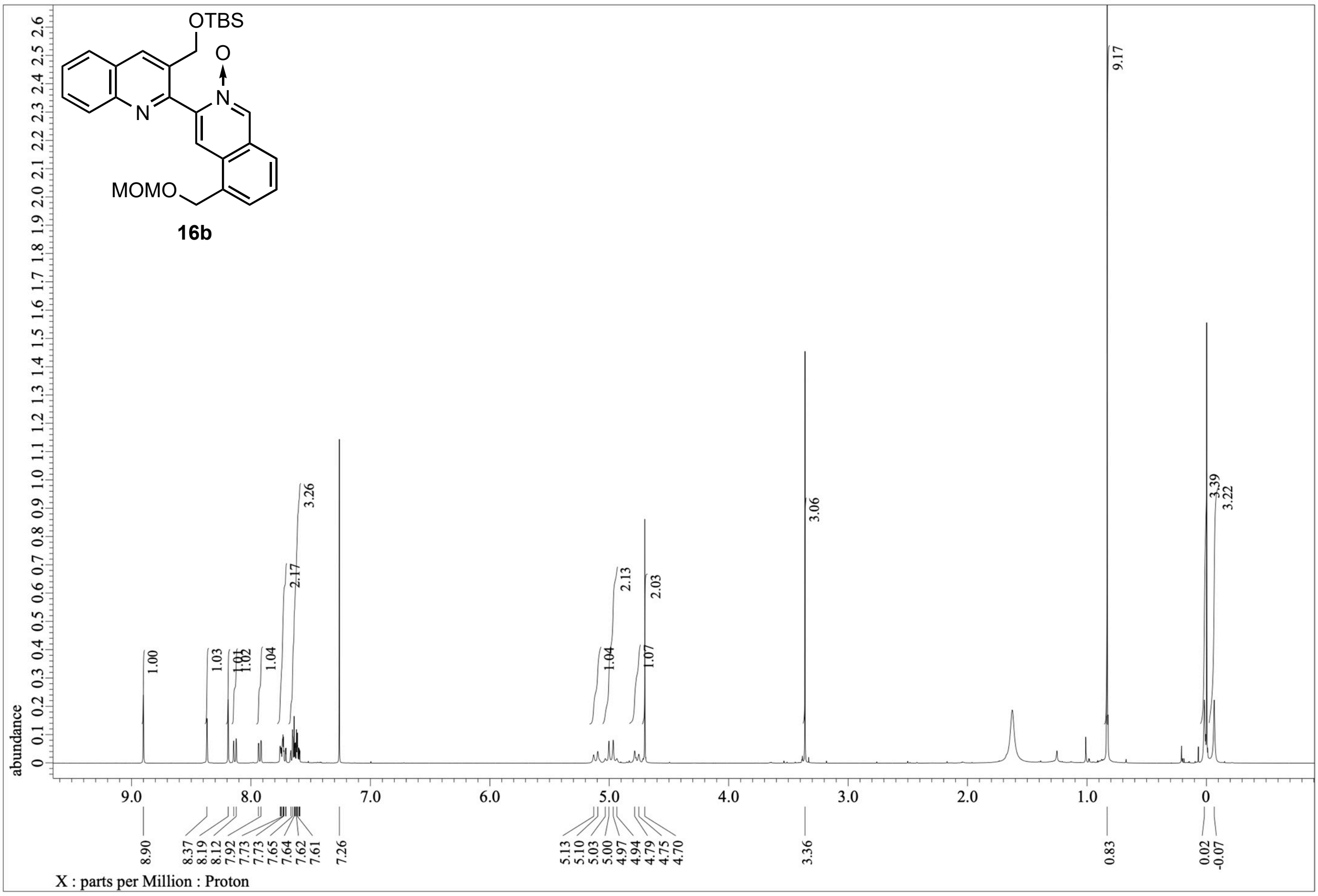

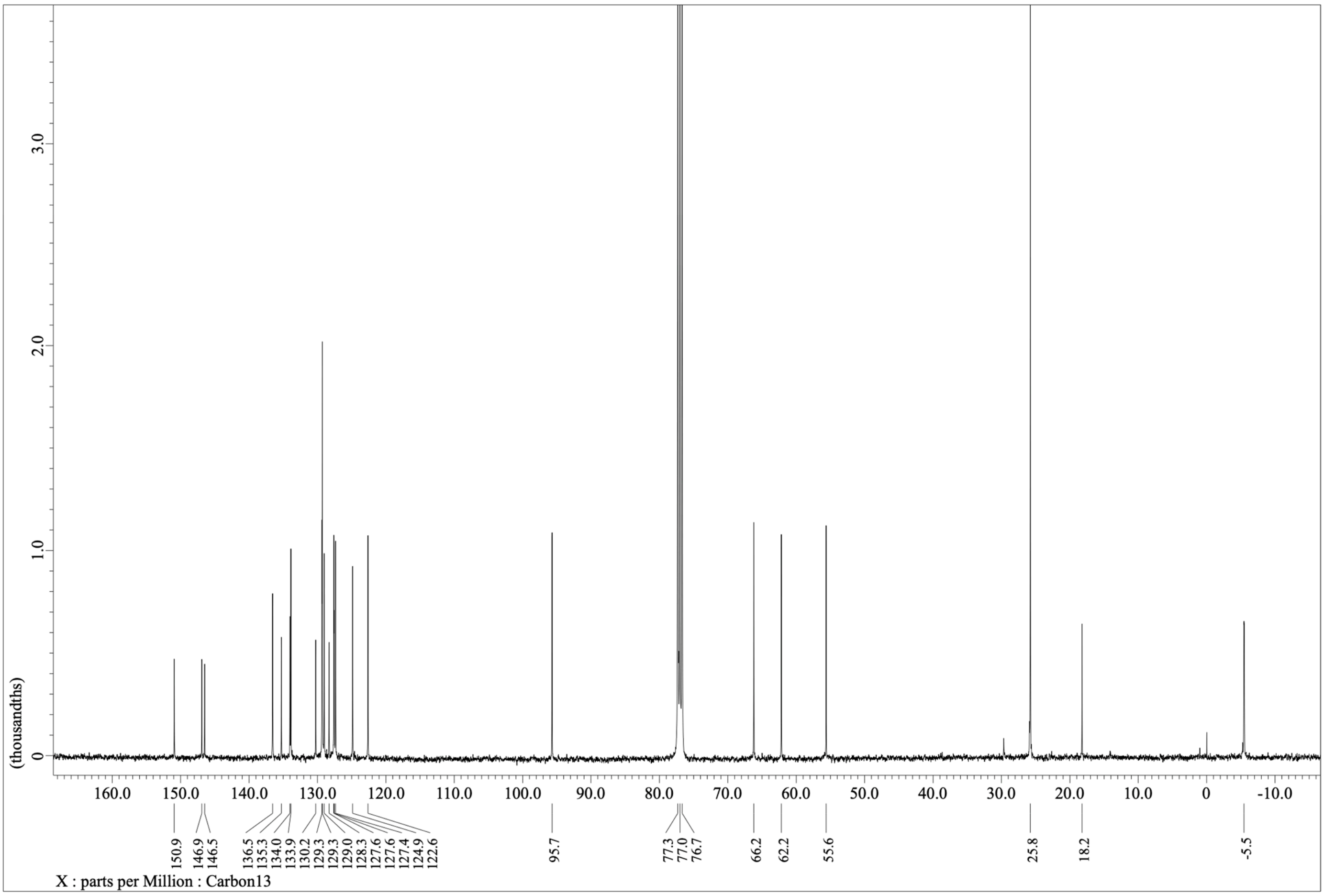

3.1.8. *2-(3-Acetoxymethylquinolin-2-yl)-5-[(methoxymethoxy)methyl]isoquinoline N-oxide* (**16c**)

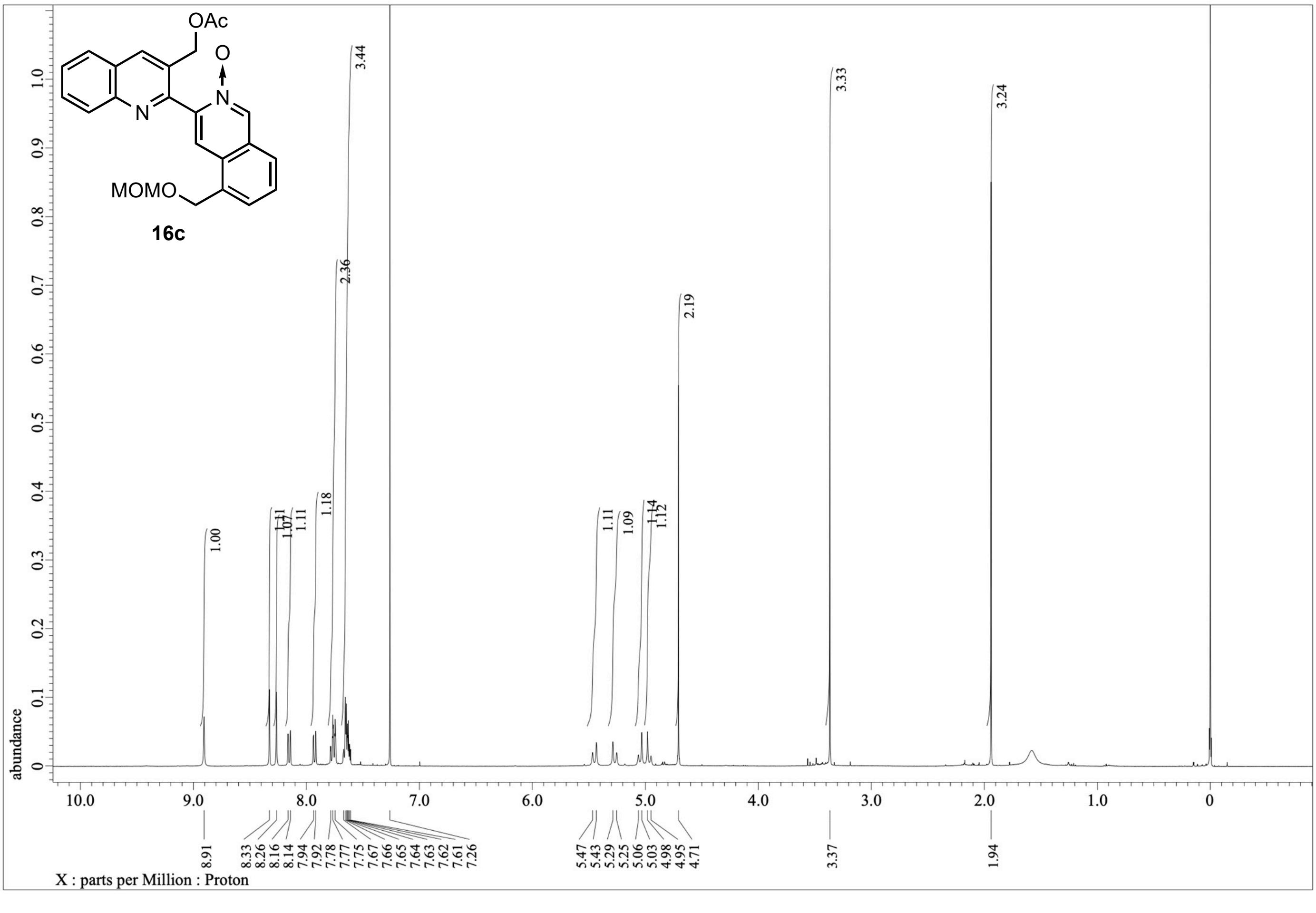

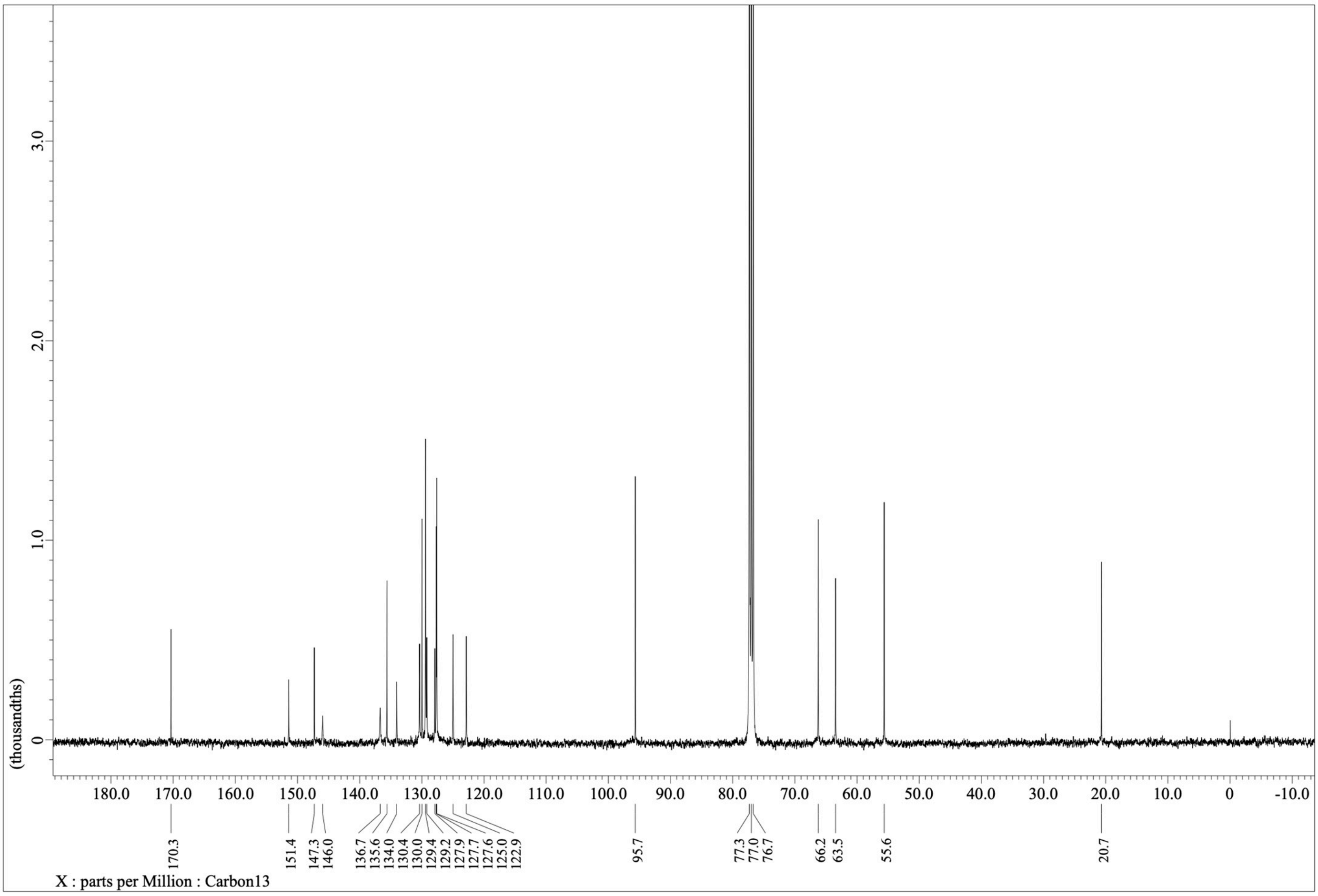

3.1.9. *3-{3-[(tert-Butyldimethylsilyloxy)methyl]quinolin-2-yl}-5-[(methoxymethoxy)methyl]isoquinolin-1-one* (**17b**)

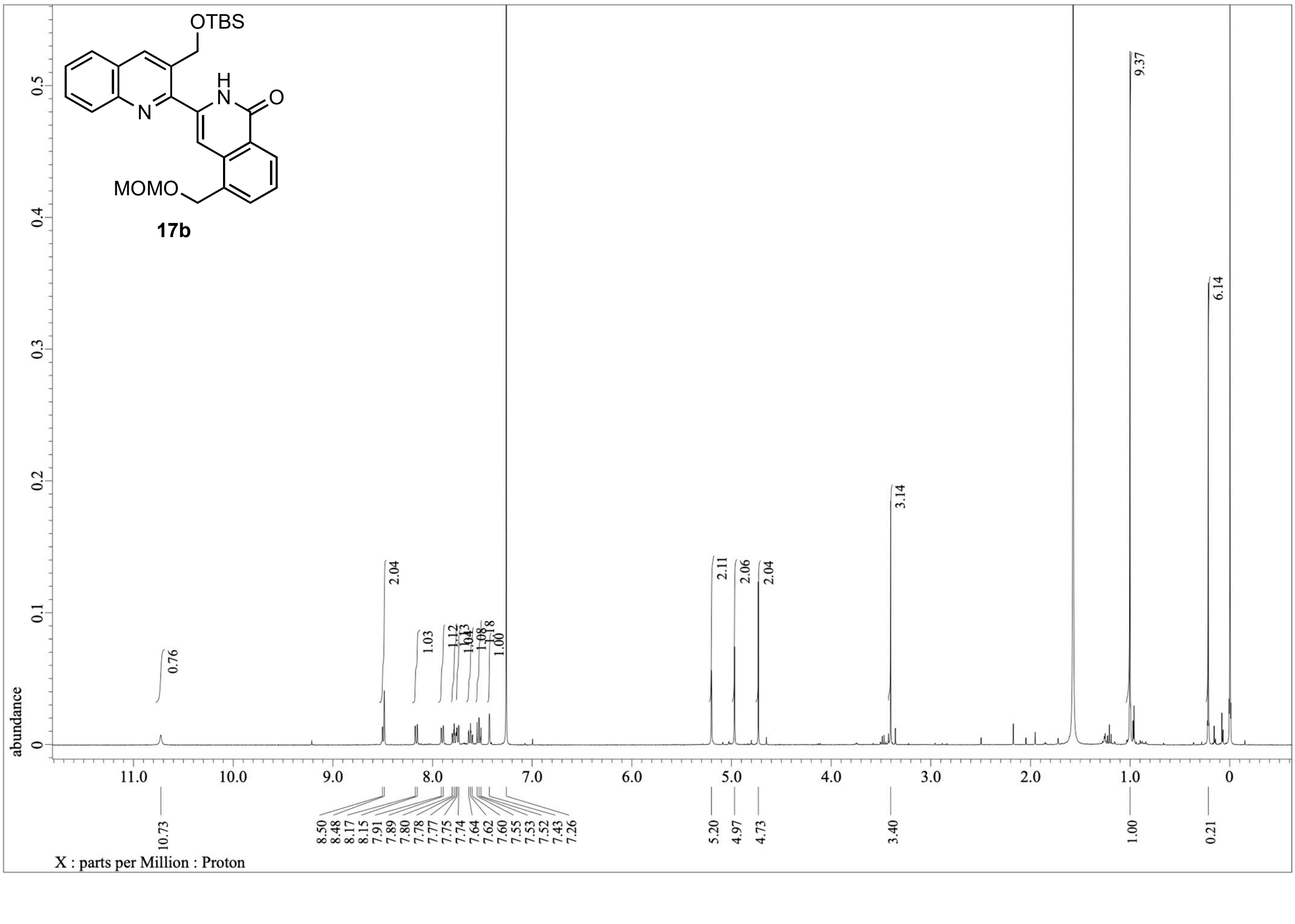

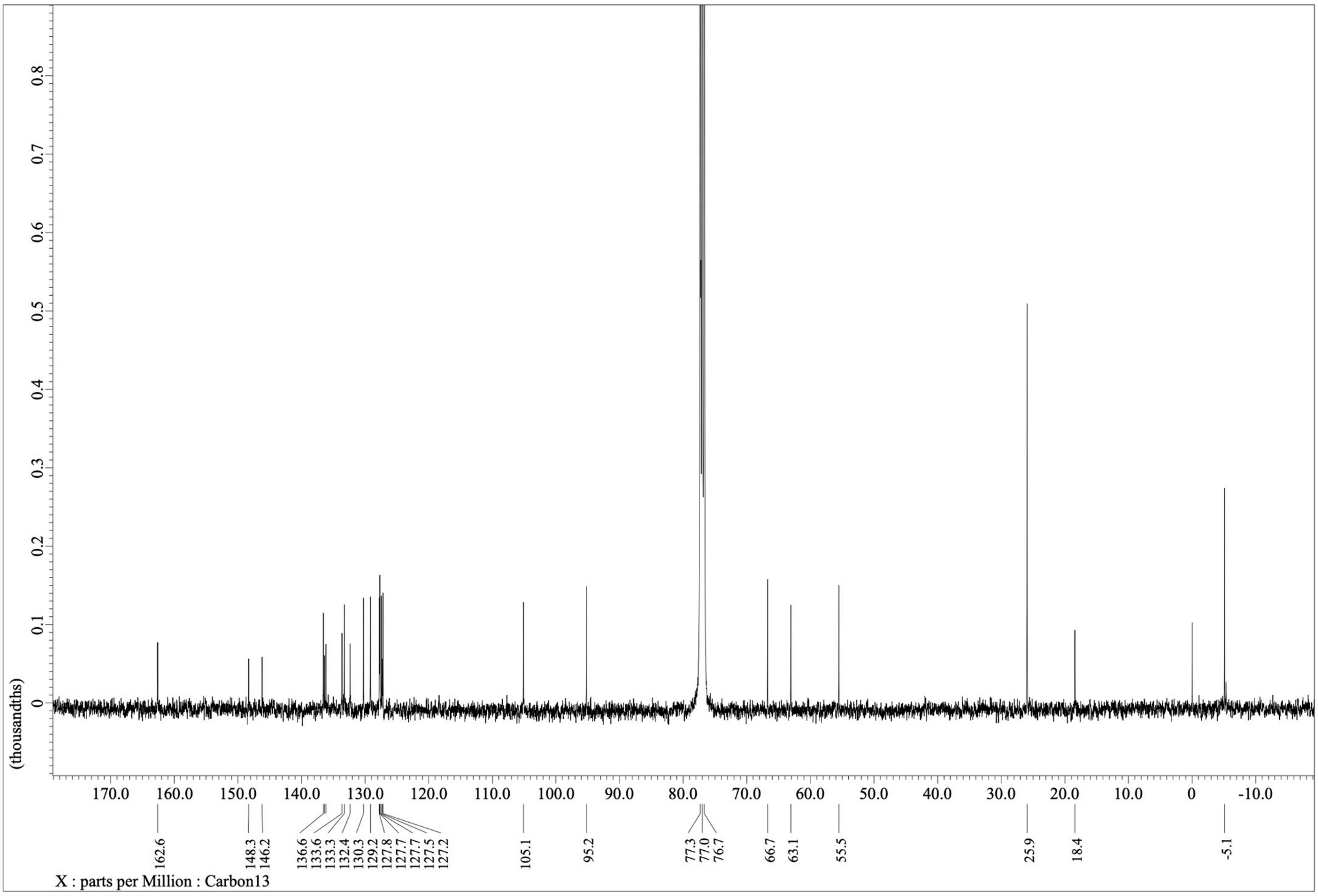

3.1.10. 3-(3-Acetoxymethylquinolin-2-yl)-5-[(methoxymethoxy)methyl]isoquinolin-1-one (17c)

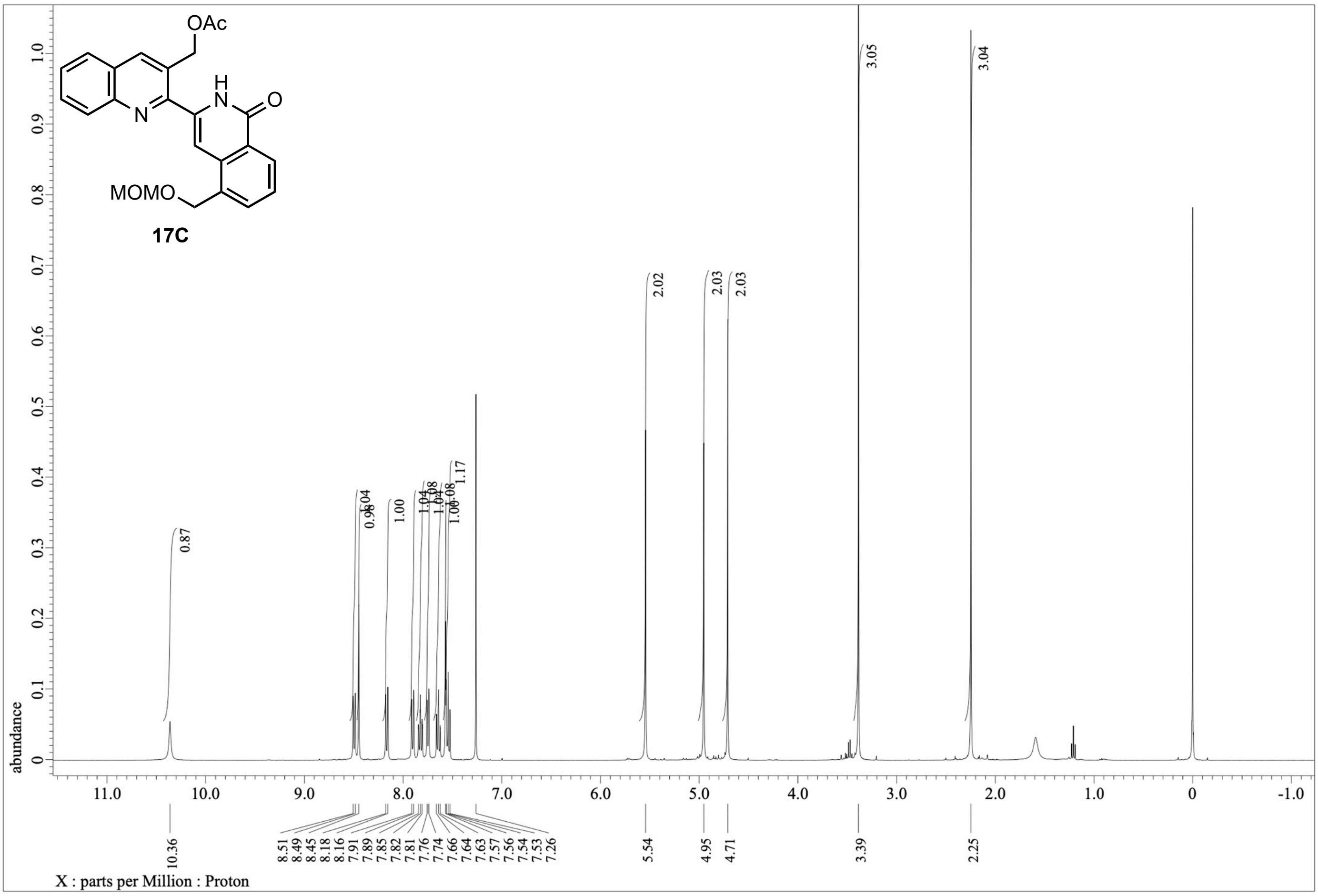

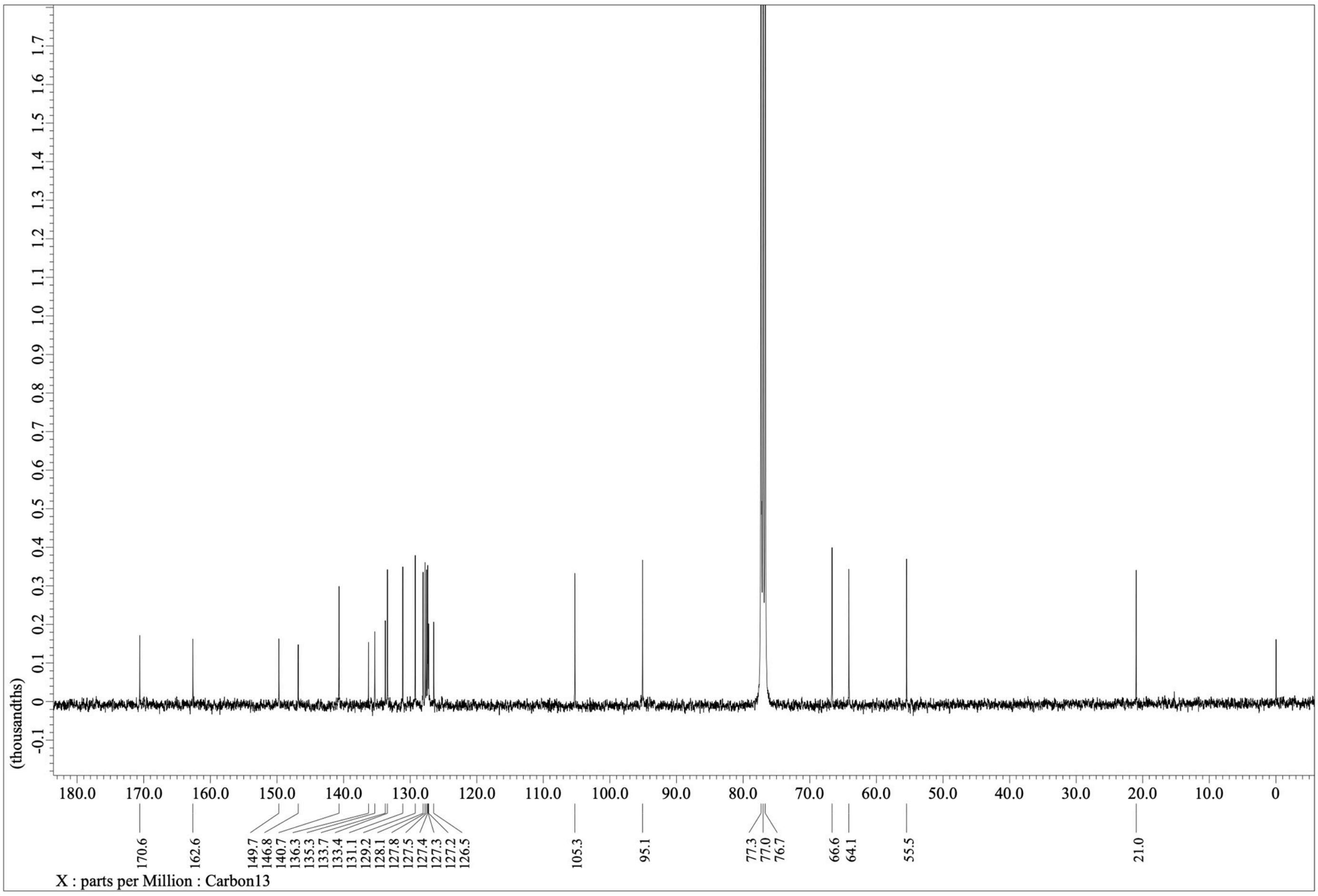

3.1.10. *3-[3-(Acetoxymethyl)quinolin-2-yl]-4-hydroxy-3-[(methoxymethoxy)methyl]isoquinoline* (**18c**)

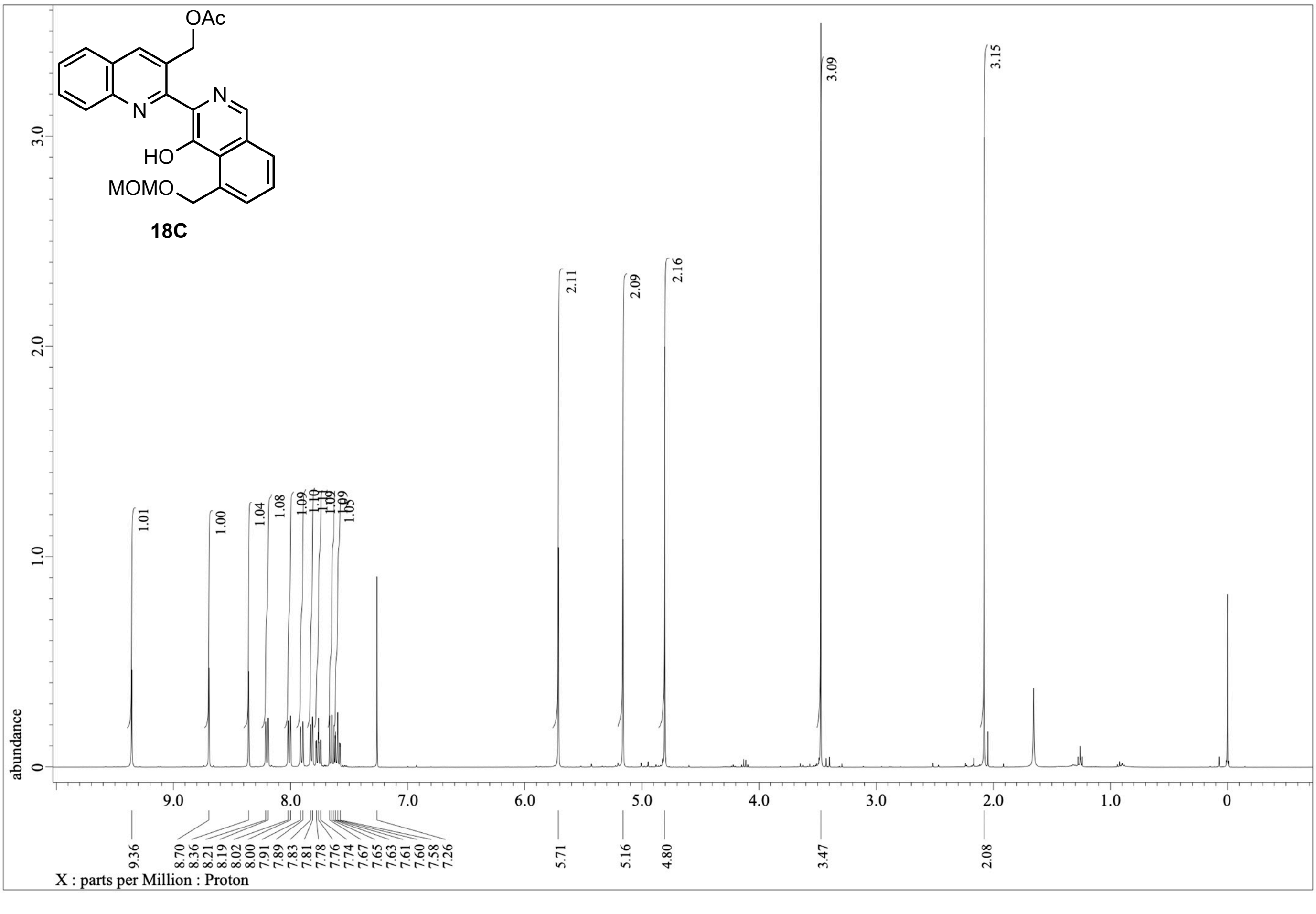

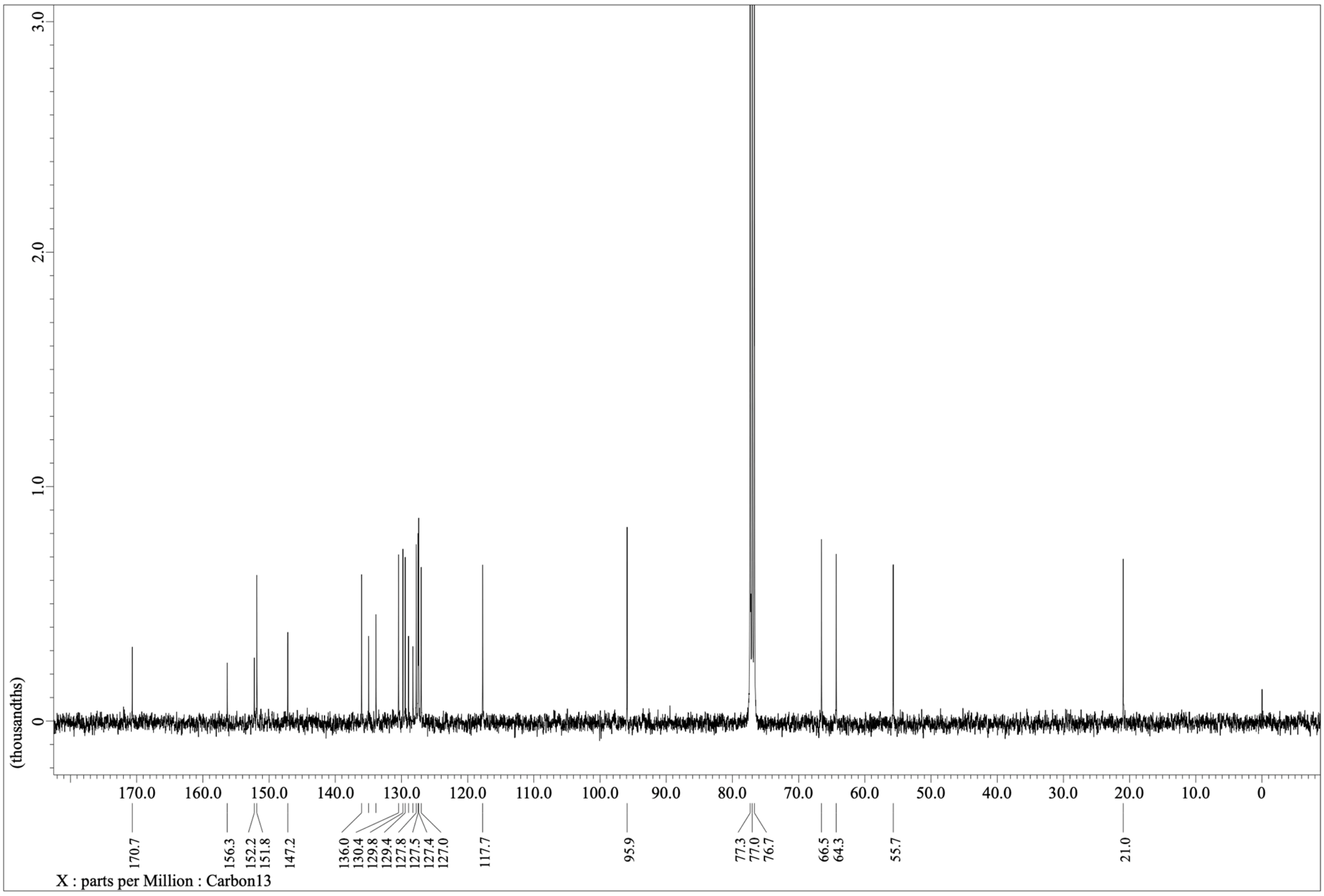

3.1.11. *3-(3-Hydroxymethylquinolin-2-yl)-5-[(methoxymethoxy)methyl]isoquinolin-1-one* (**19**)

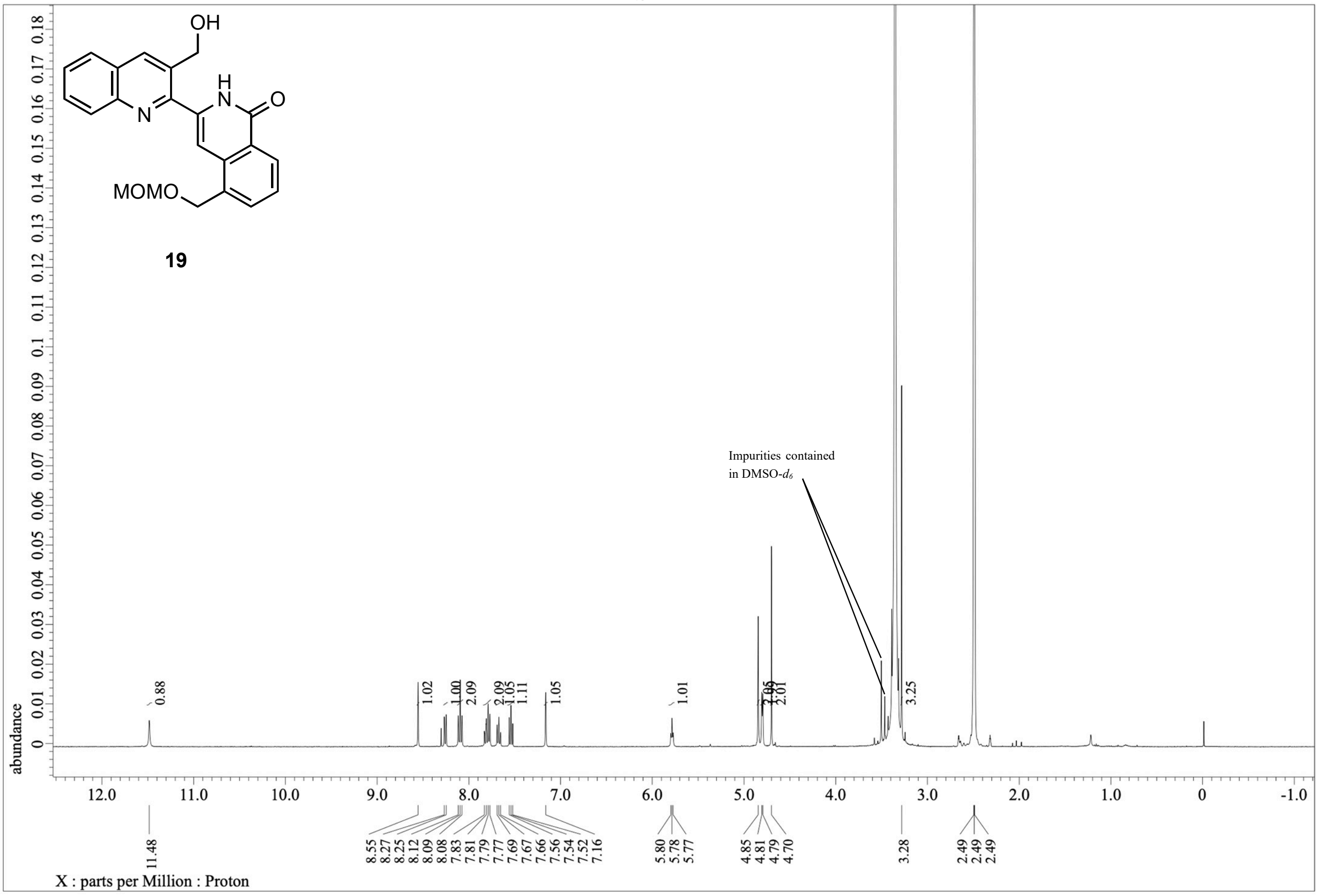

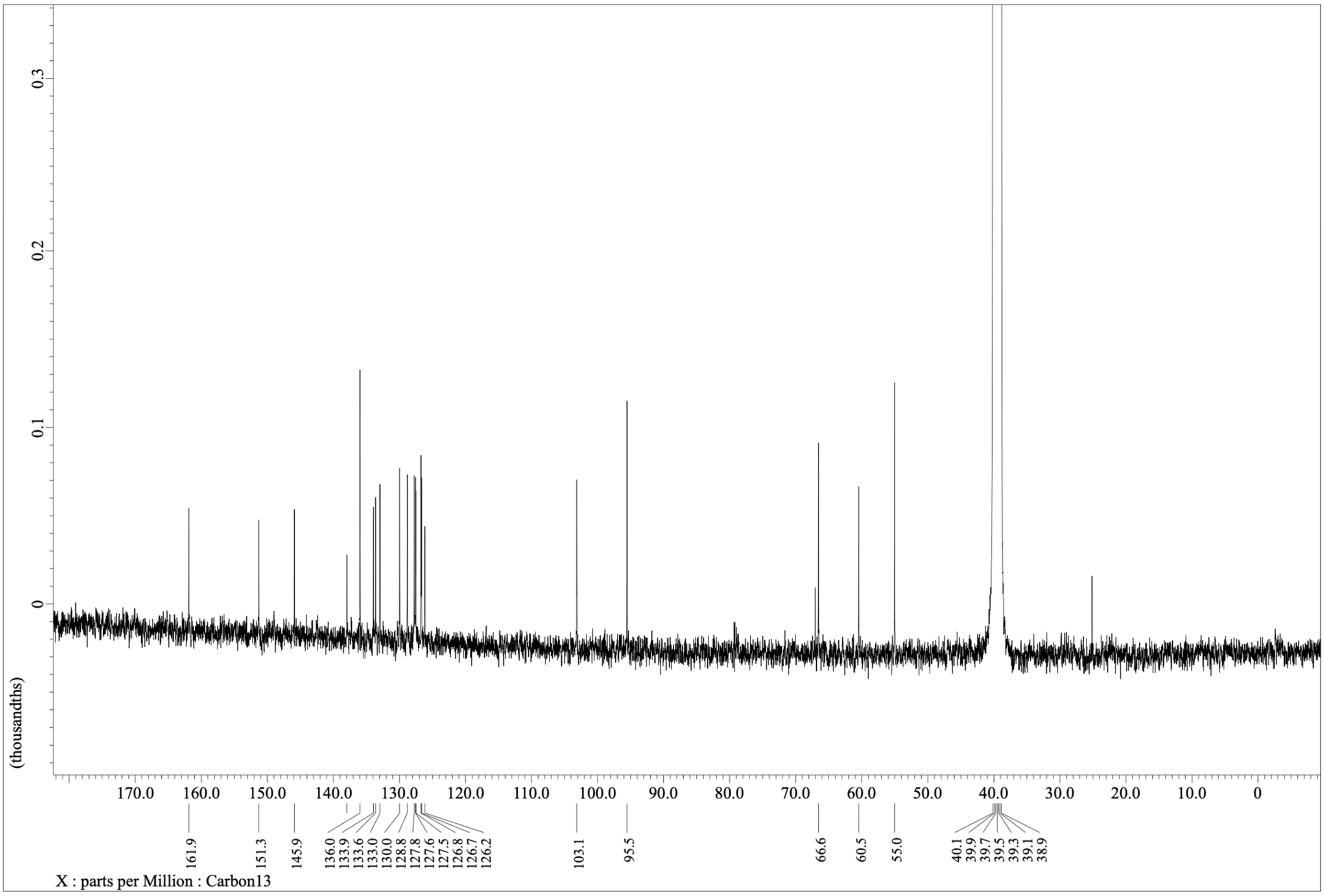

3.1.12. *22-Hydroxyacuminatine* (**4**)

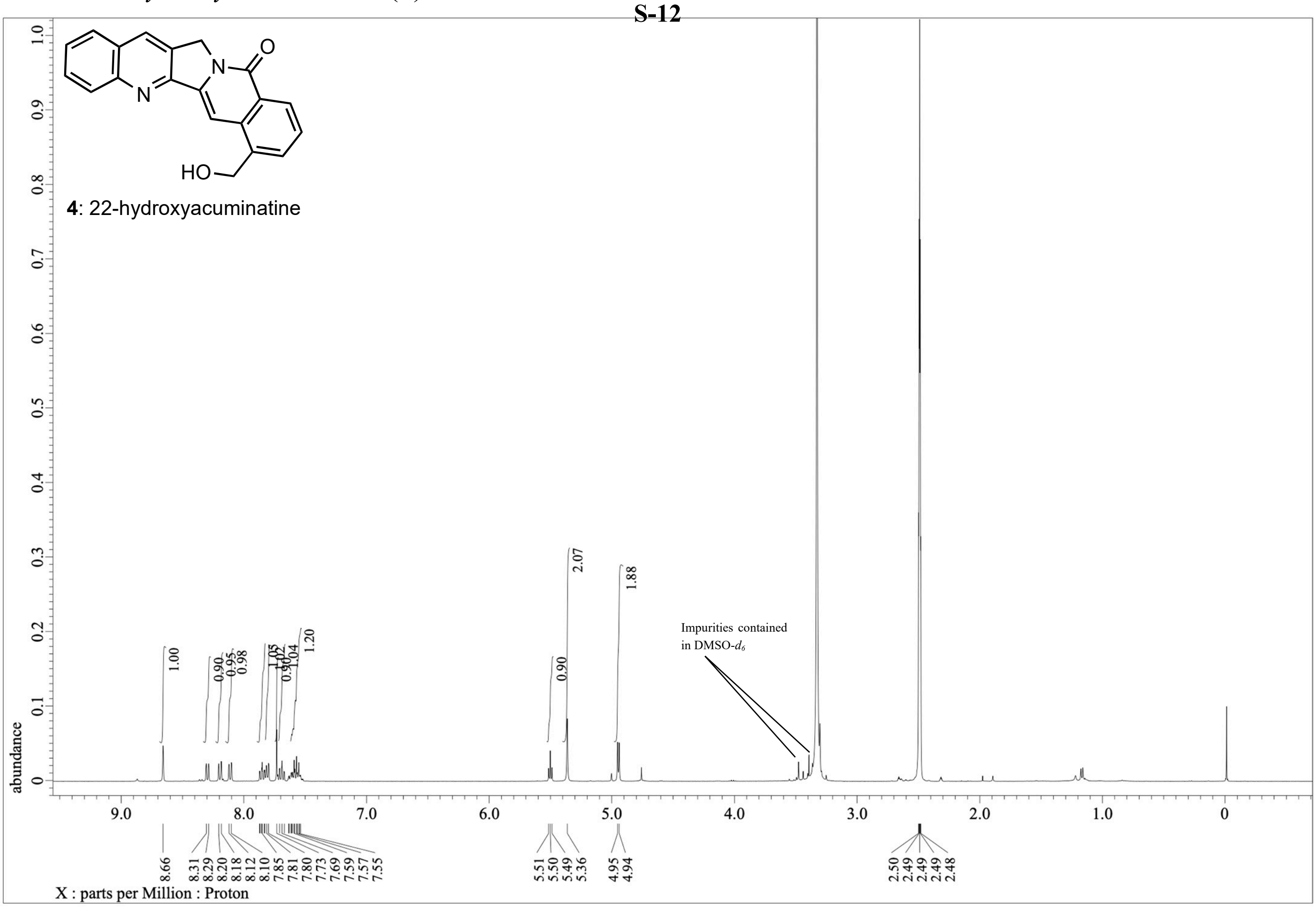

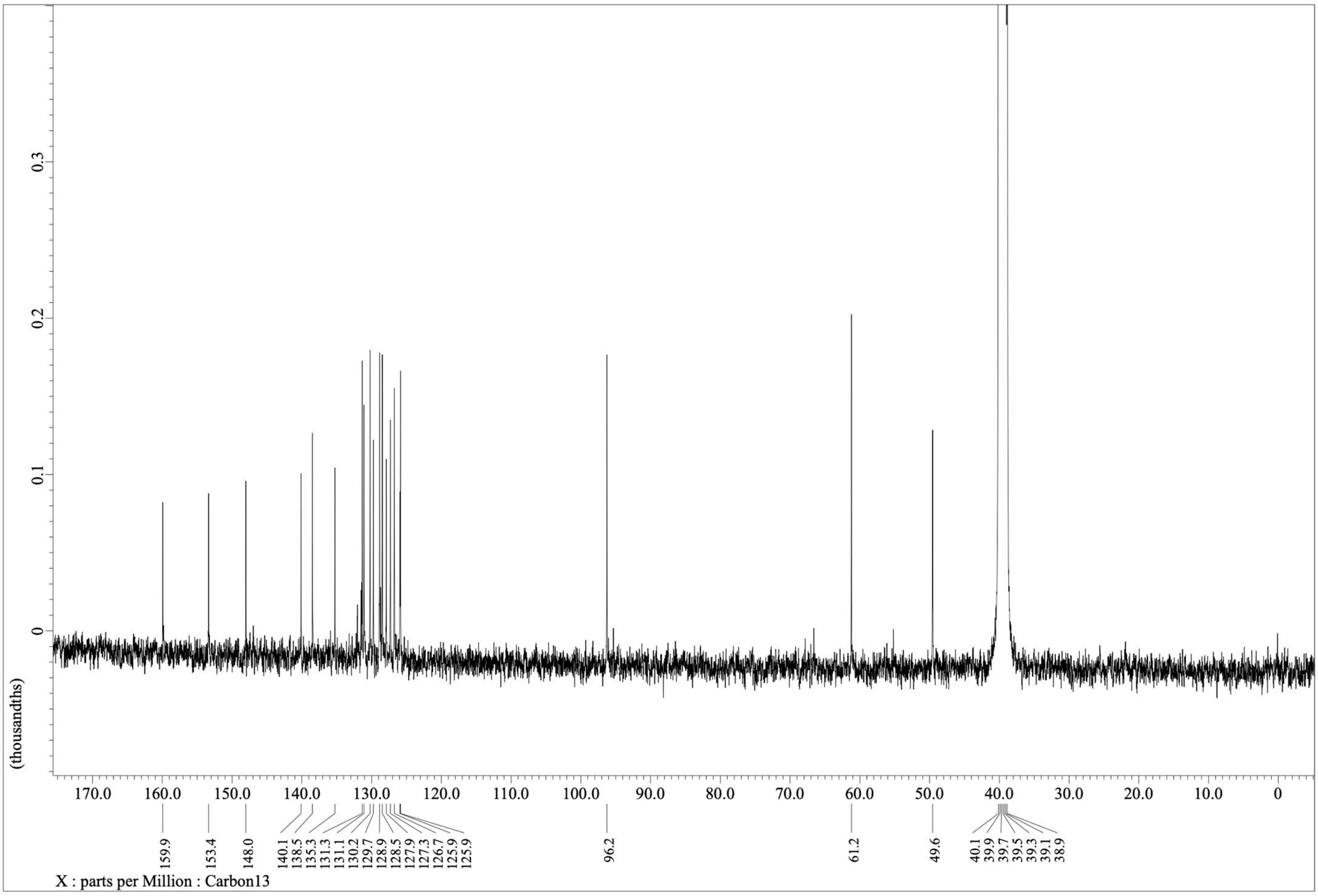

3.1.13. *Methyl [2-iodo-N-(methoxymethyl)indol-3-yl]acetate* (**22**)

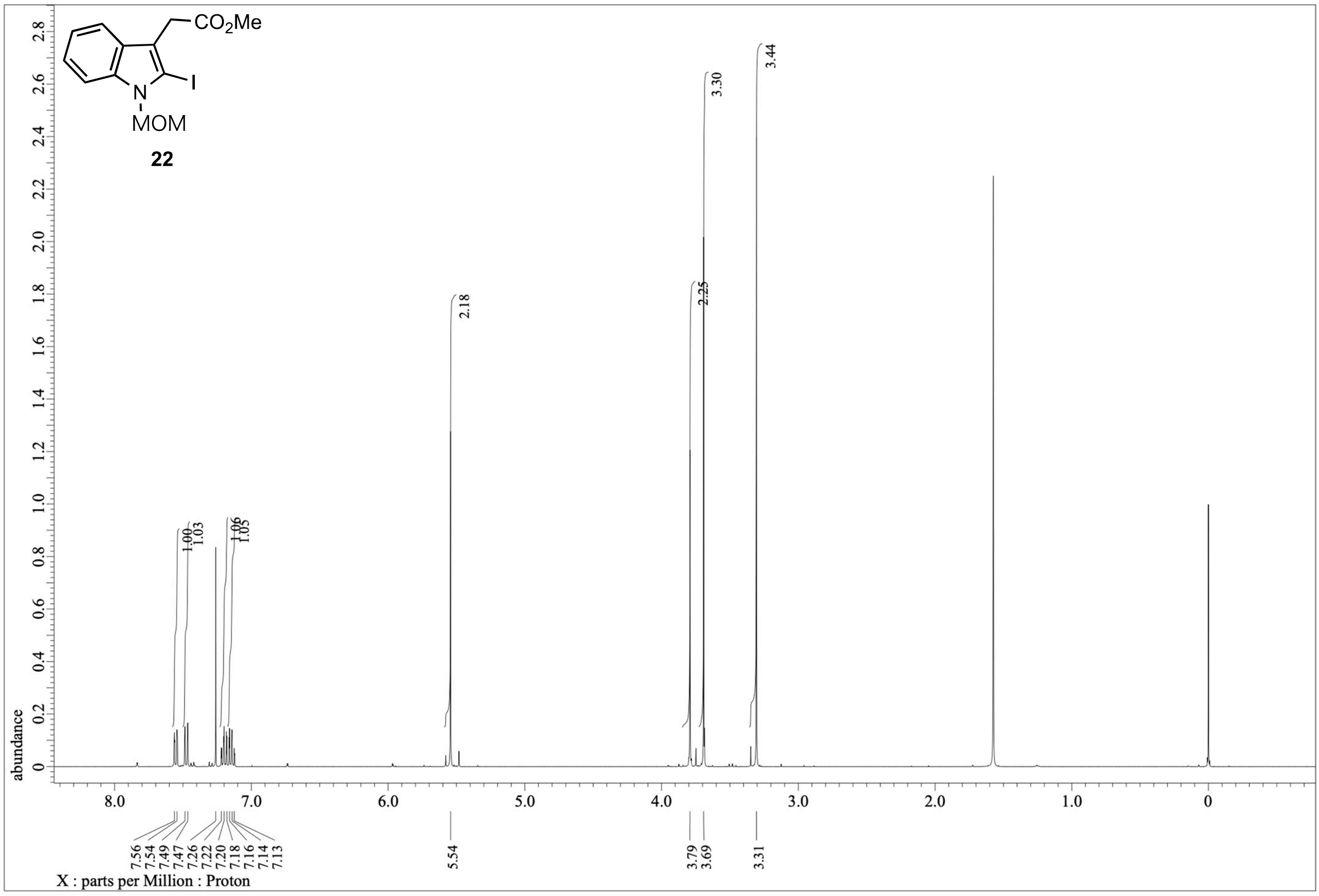

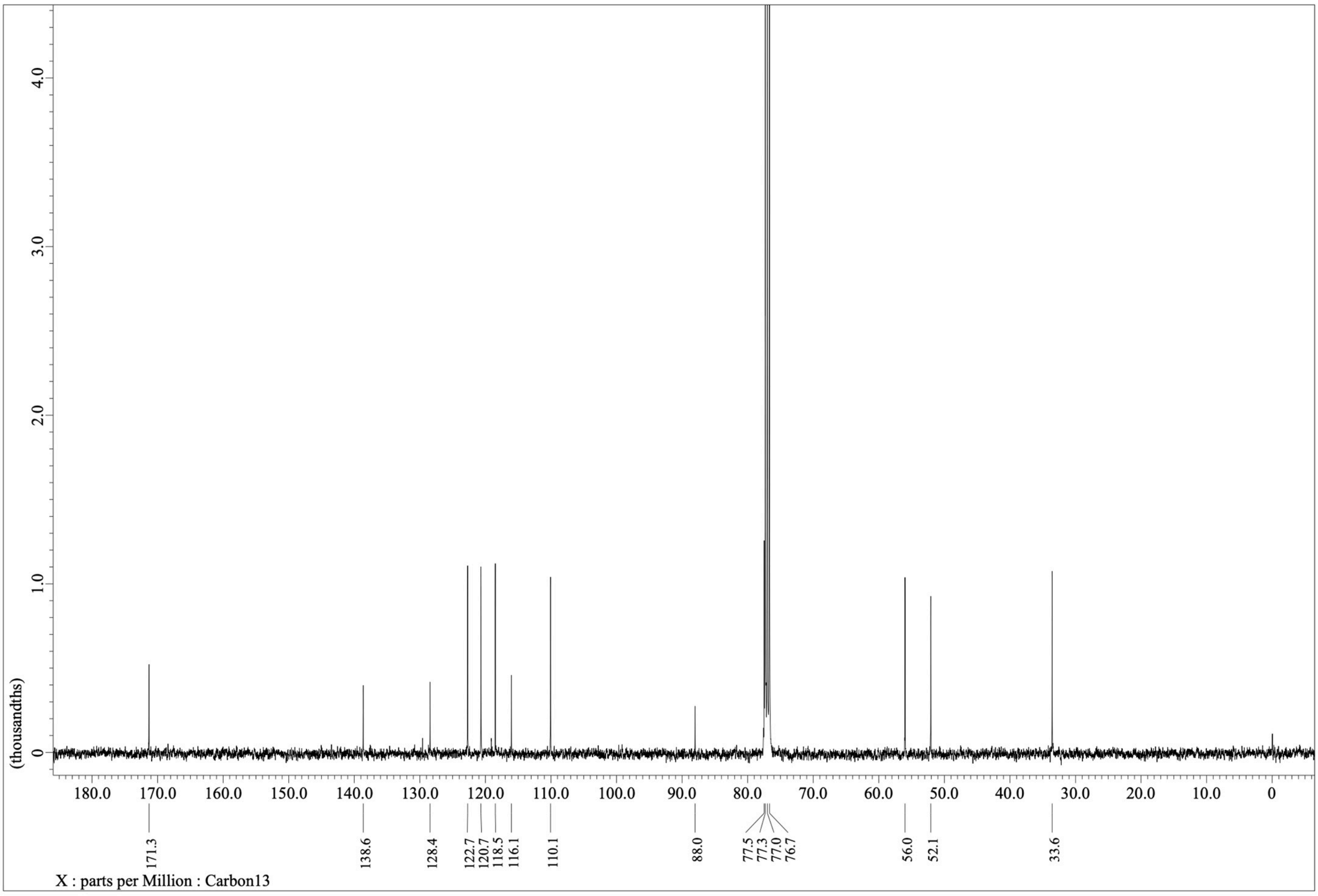

3.1.14. Methyl [2-trimethylsilylethynyl-N-(methoxymethyl)lindol-3-yl]acetate (**24**)

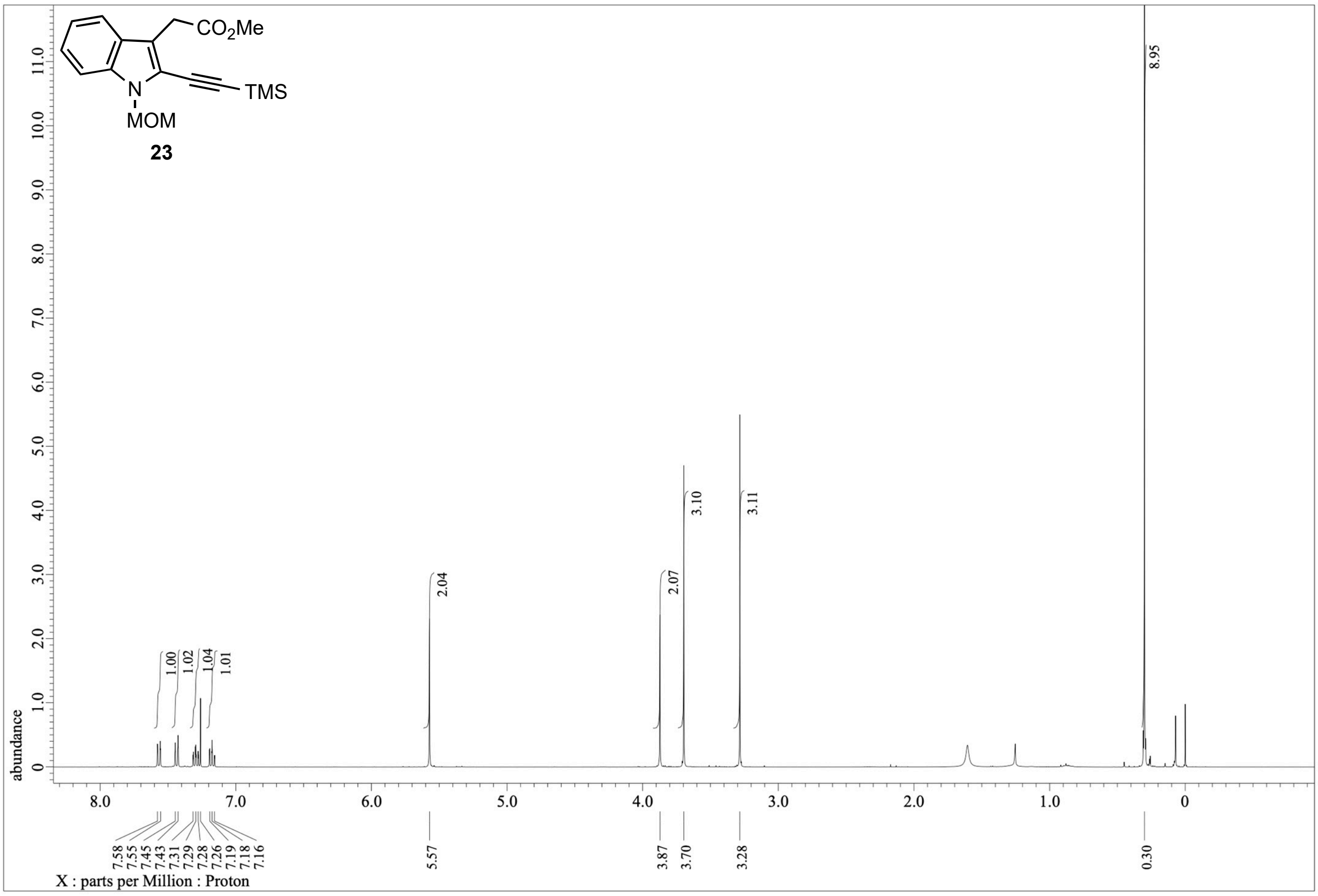

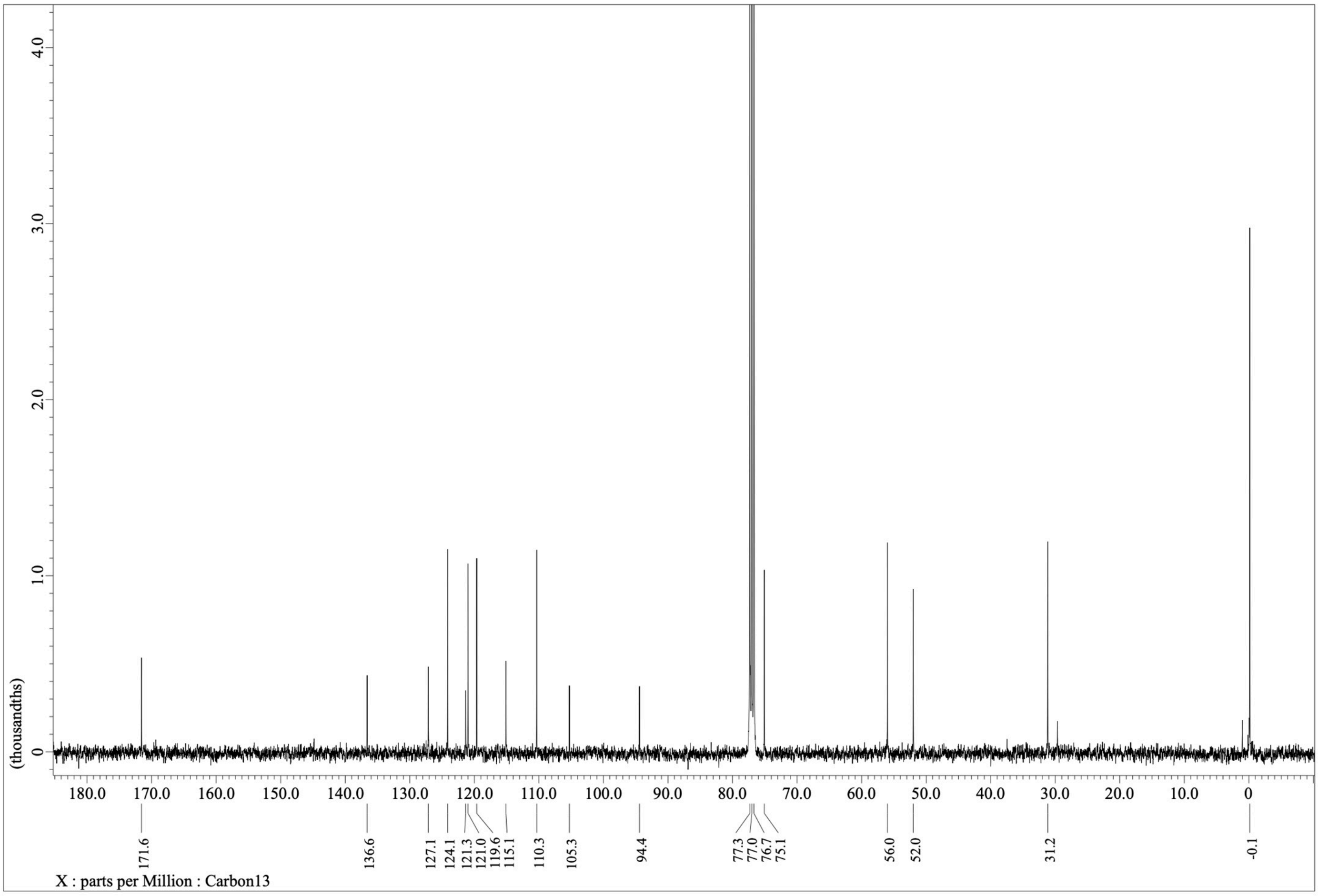

3.1.15. *Methyl [2-ethynyl-N-(methoxymethyl)indol-3-yl]acetate* (**24**)

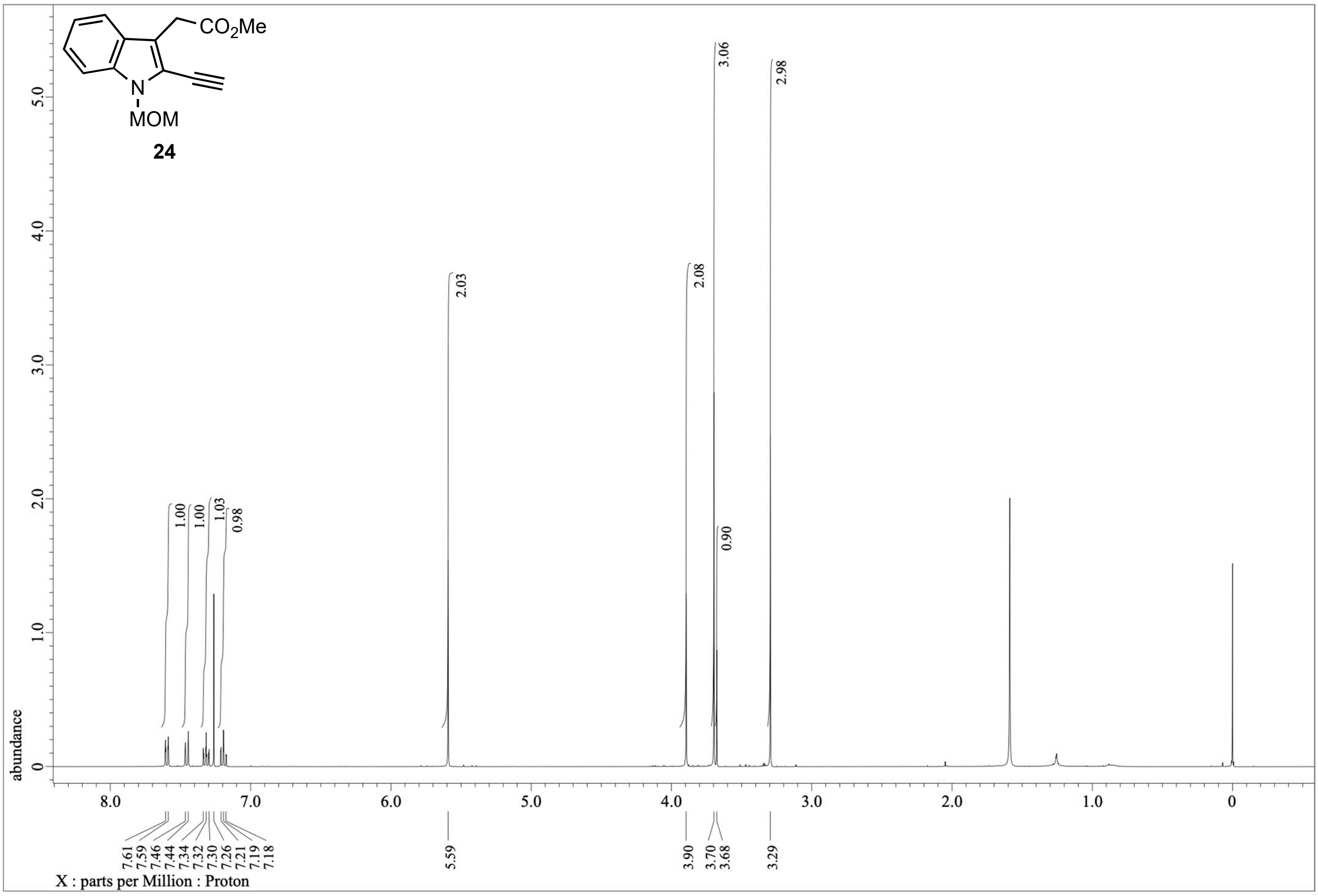

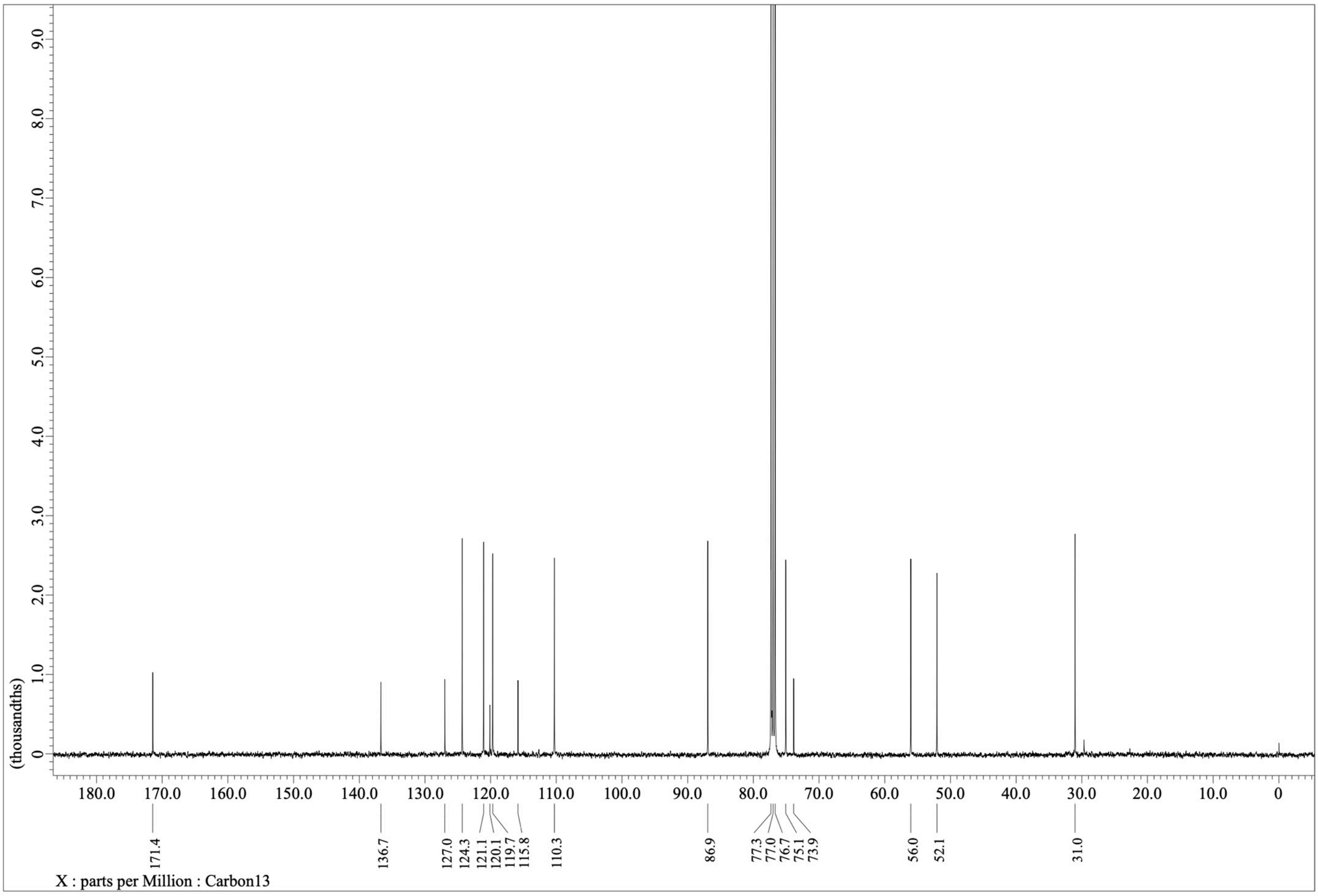

3.1.16. *Methyl {2-[2-(2-formylphenyl)ethynyl]-N-(methoxymethyl)indol-3-yl}acetate* (**26a**)

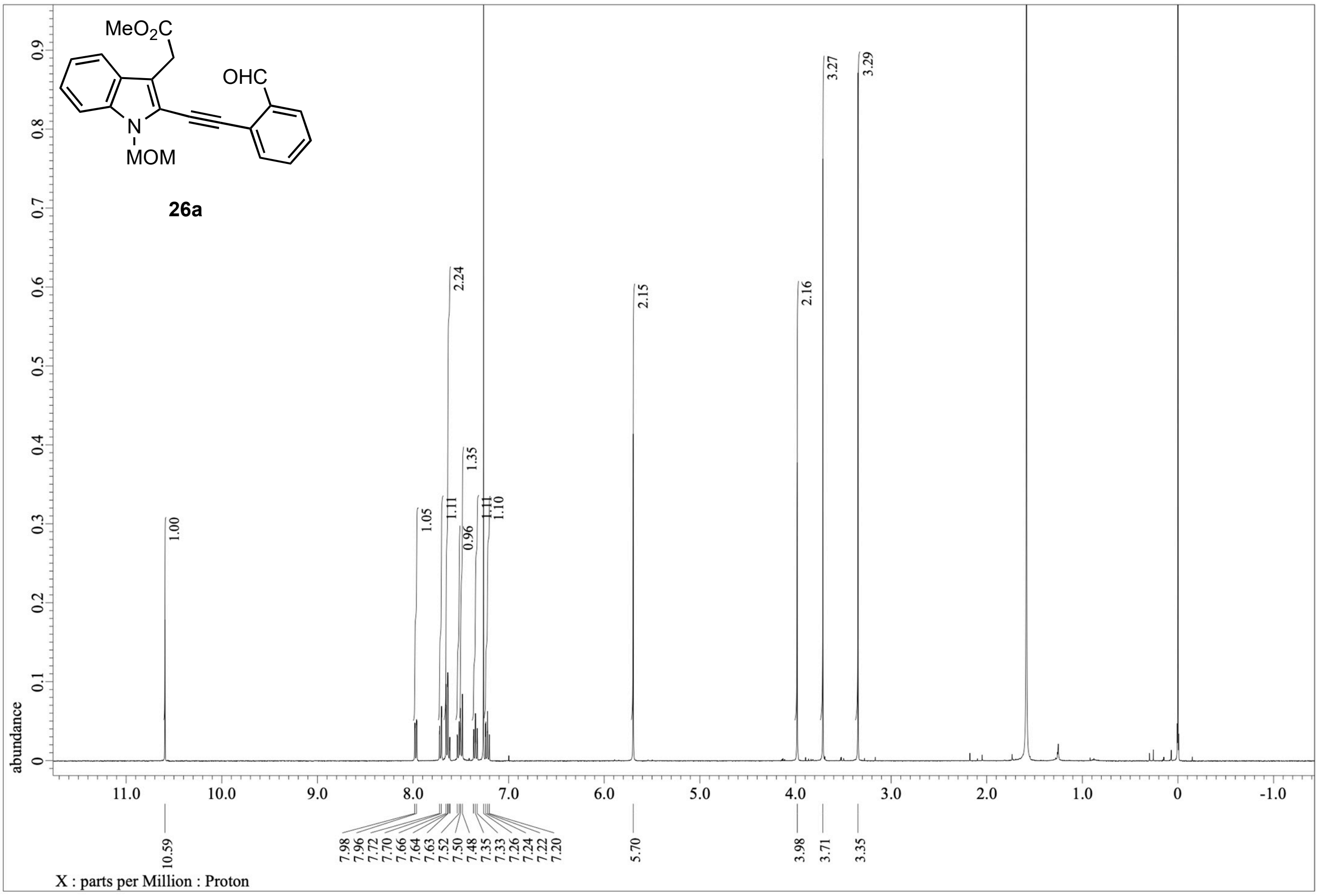

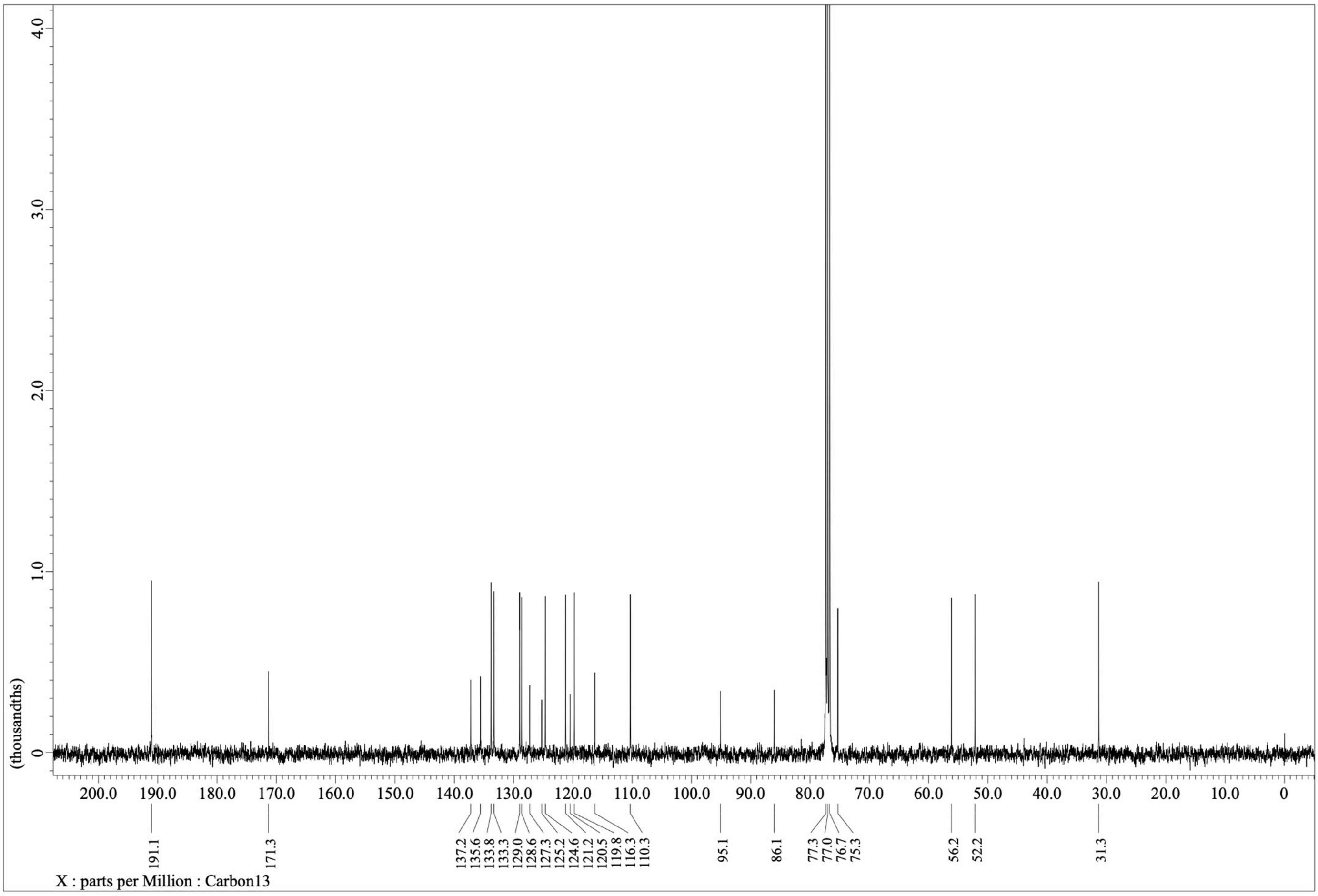

3.1.17. *Methyl {2-[2-(2-formyl-3-[(methoxymethoxy)methyl]phenyl)ethynyl] -N-(methoxymethylindol-3-yl}acetate* (**26b**)

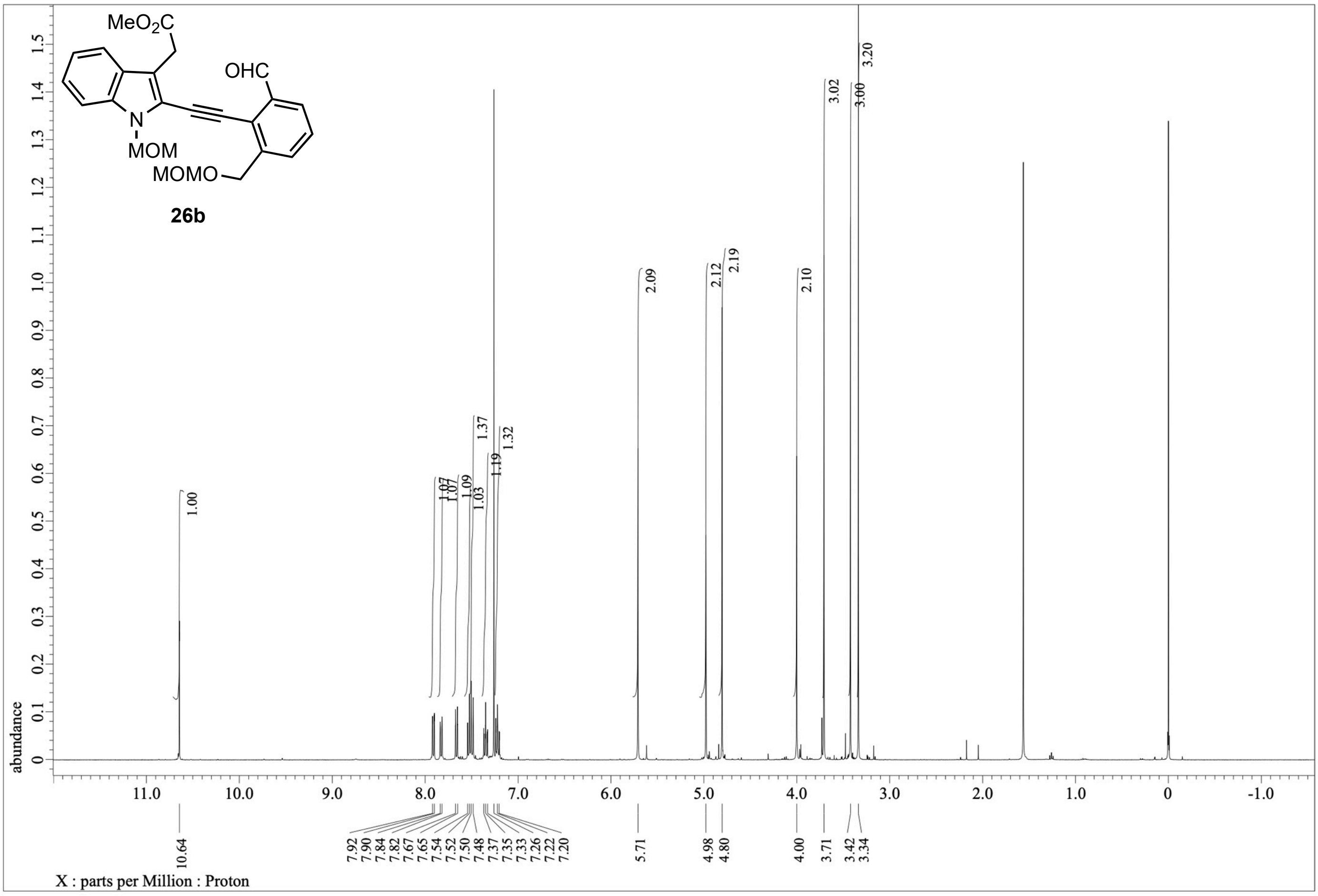

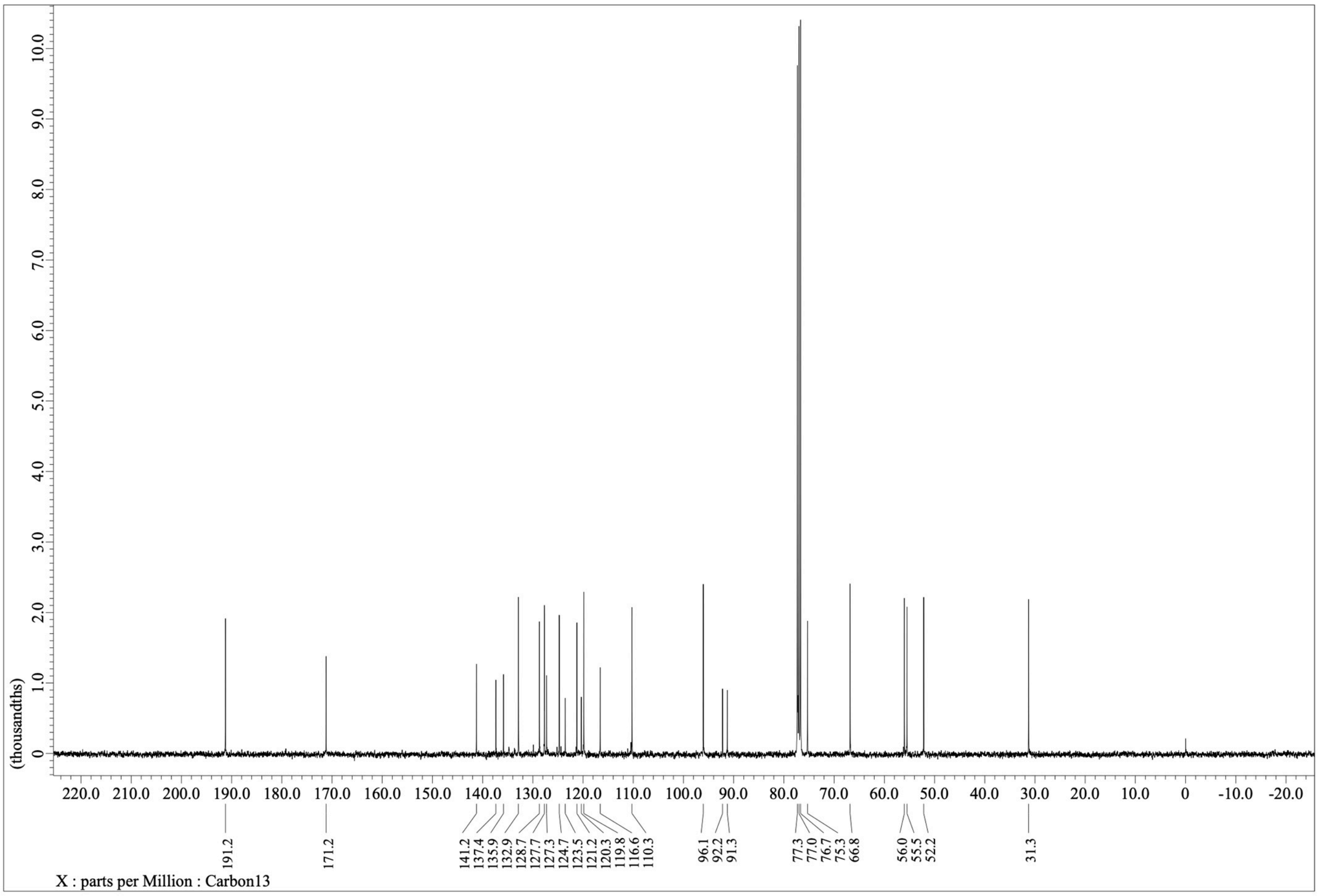

3.1.18. *Methyl {2-[22-hydroxyiminophenyl)ethynyl]-N-(methoxymethyl)indol-3-yl}acetate* (**27a**)

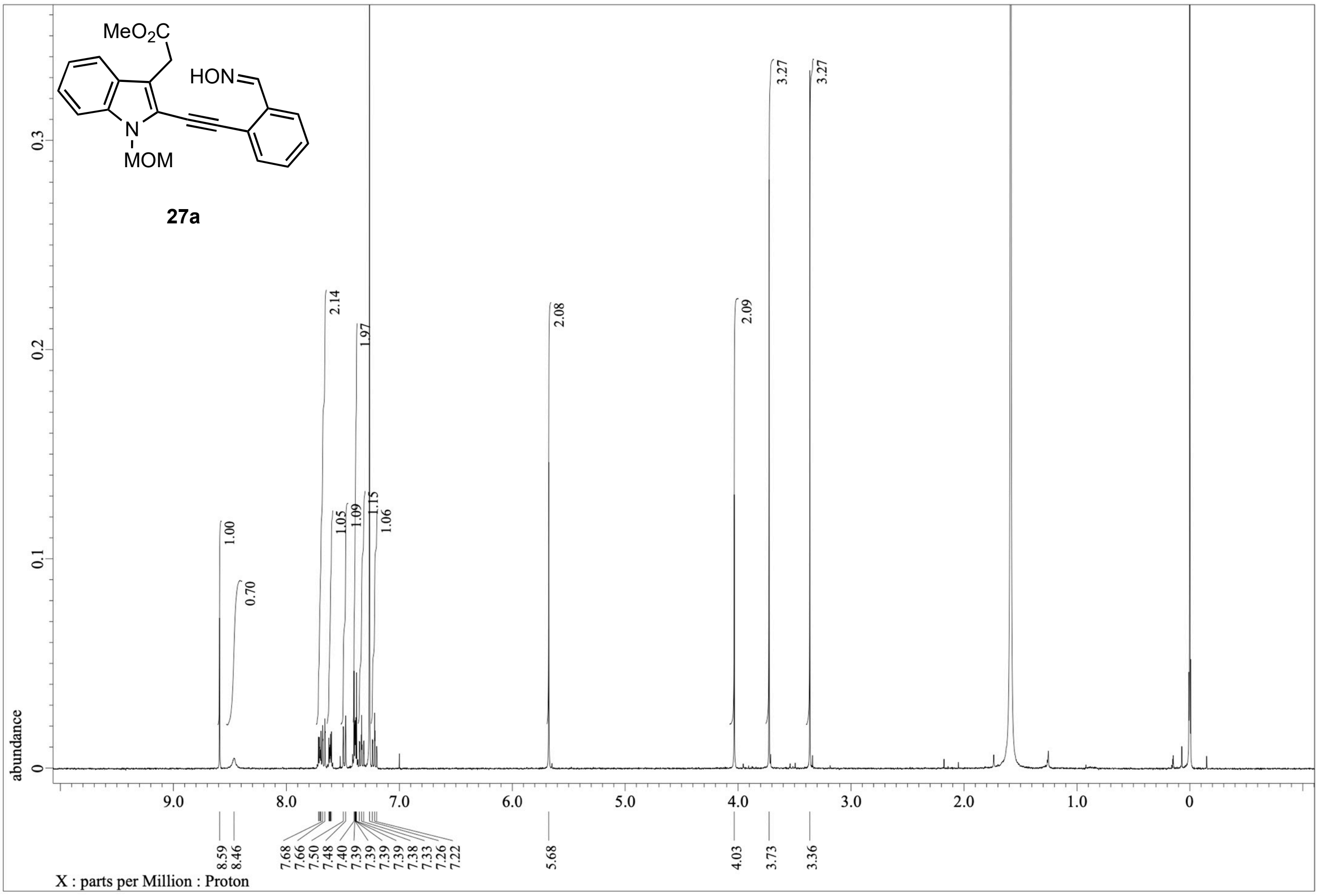

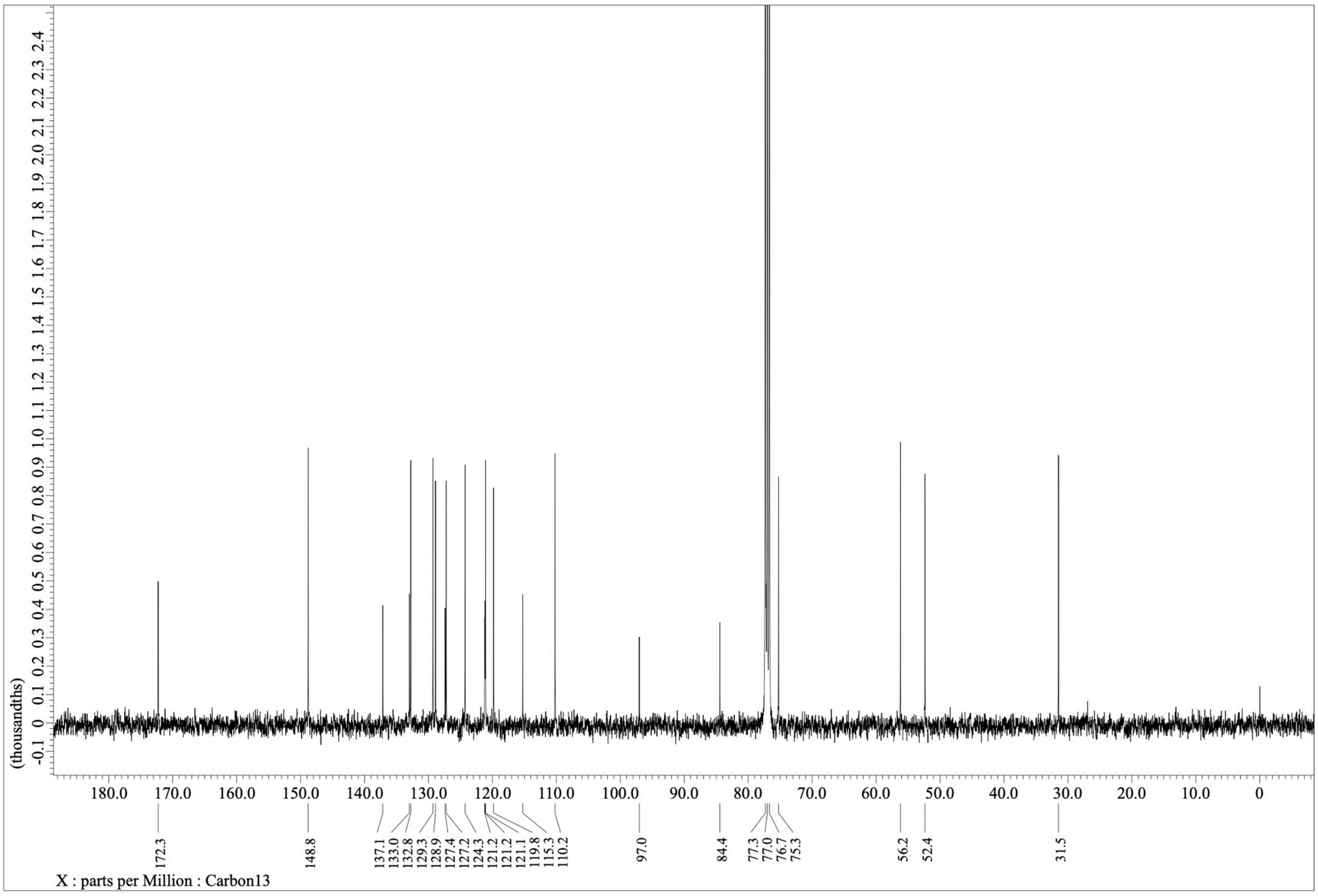

3.1.19. *Methyl {2-[2-(2-hydroxyimino-3-[(methoxymethoxy)methyl]phenyl)ethynyl]*

-N-methoxymethylindol-3-yl}acetate (**27b**)

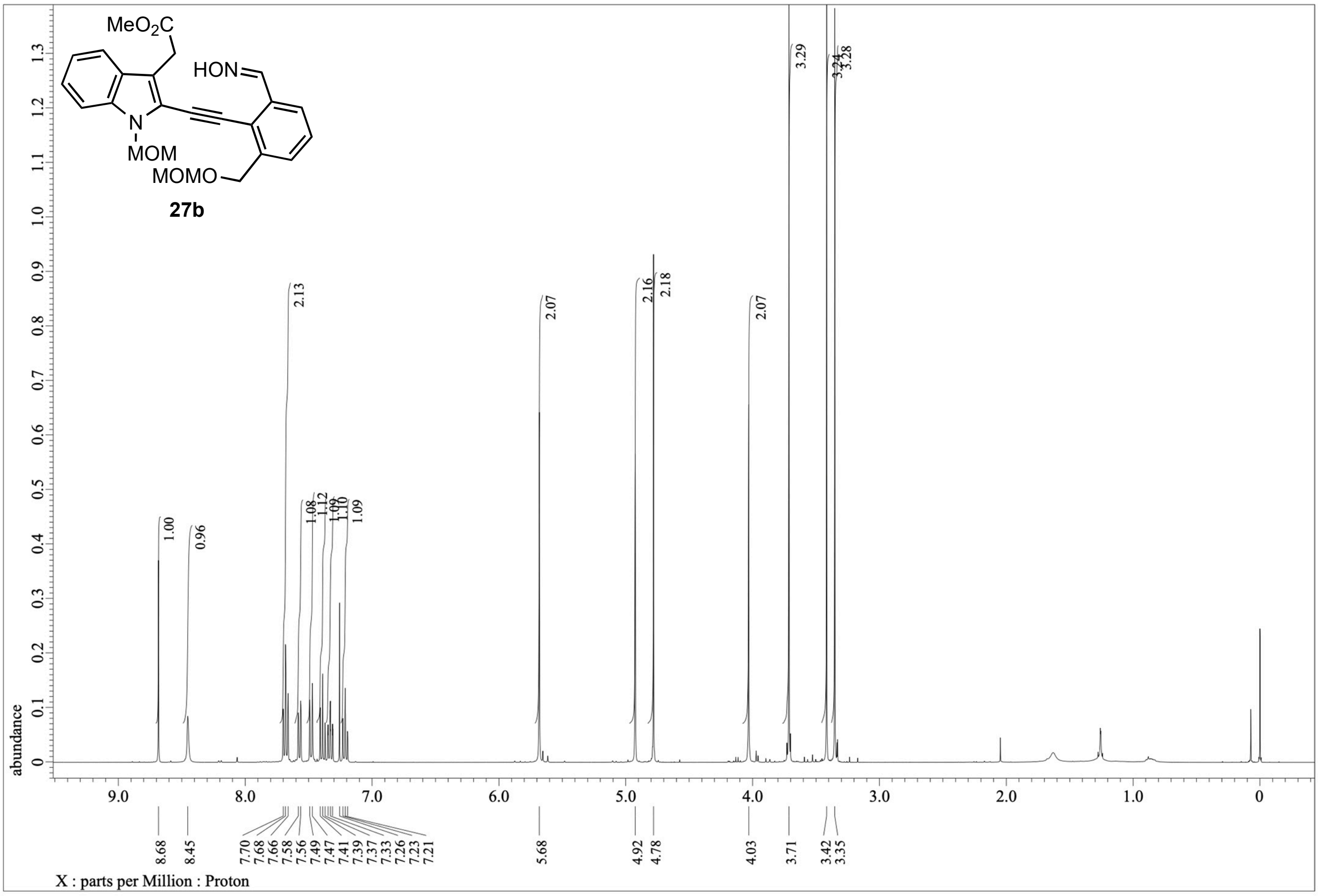

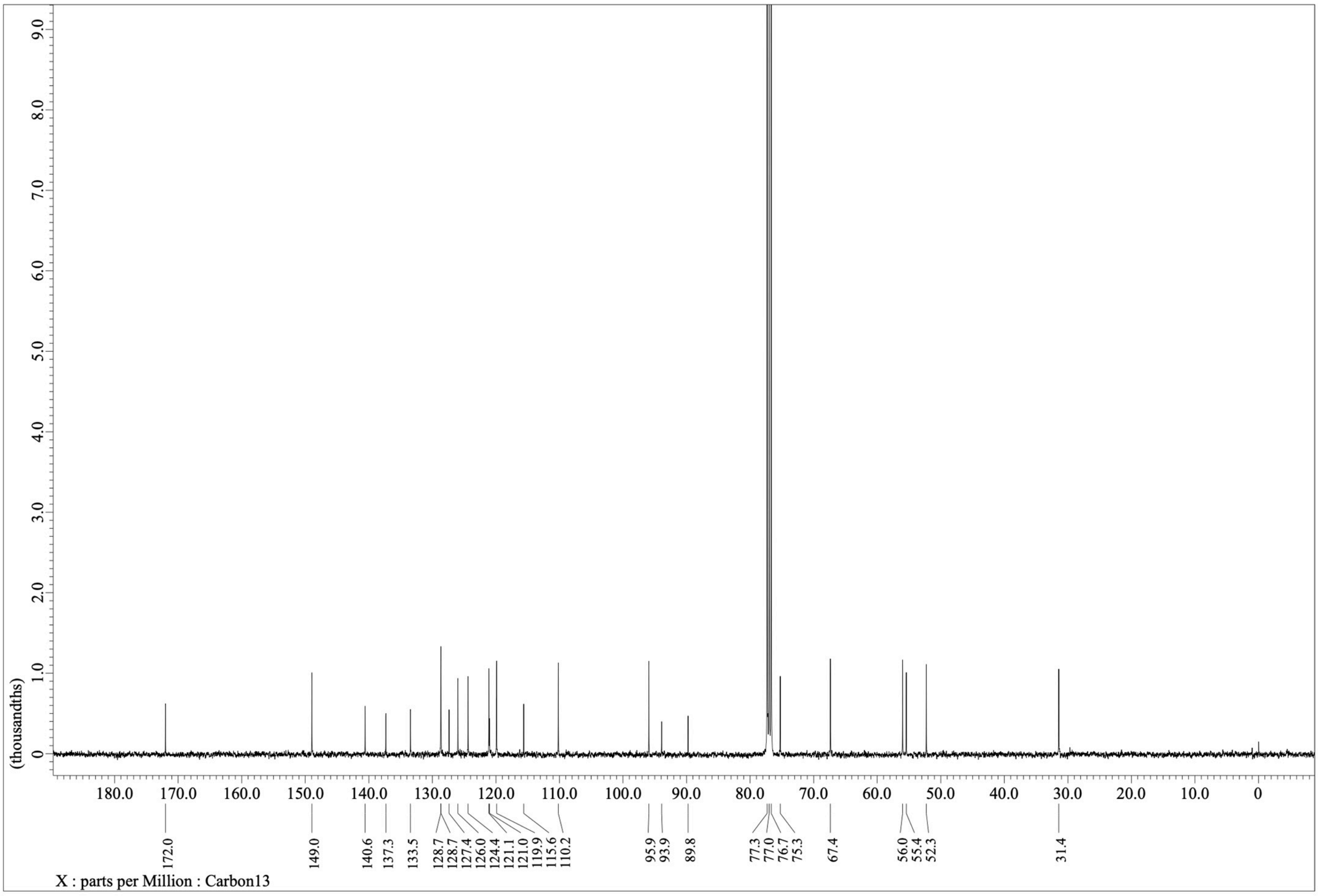

3.1.20. *3-[3-(2-Methoxy-2-oxoethyl)-1-(methoxymethyl)indol-2-yl]isoquinoline N-oxide* (**28a**)

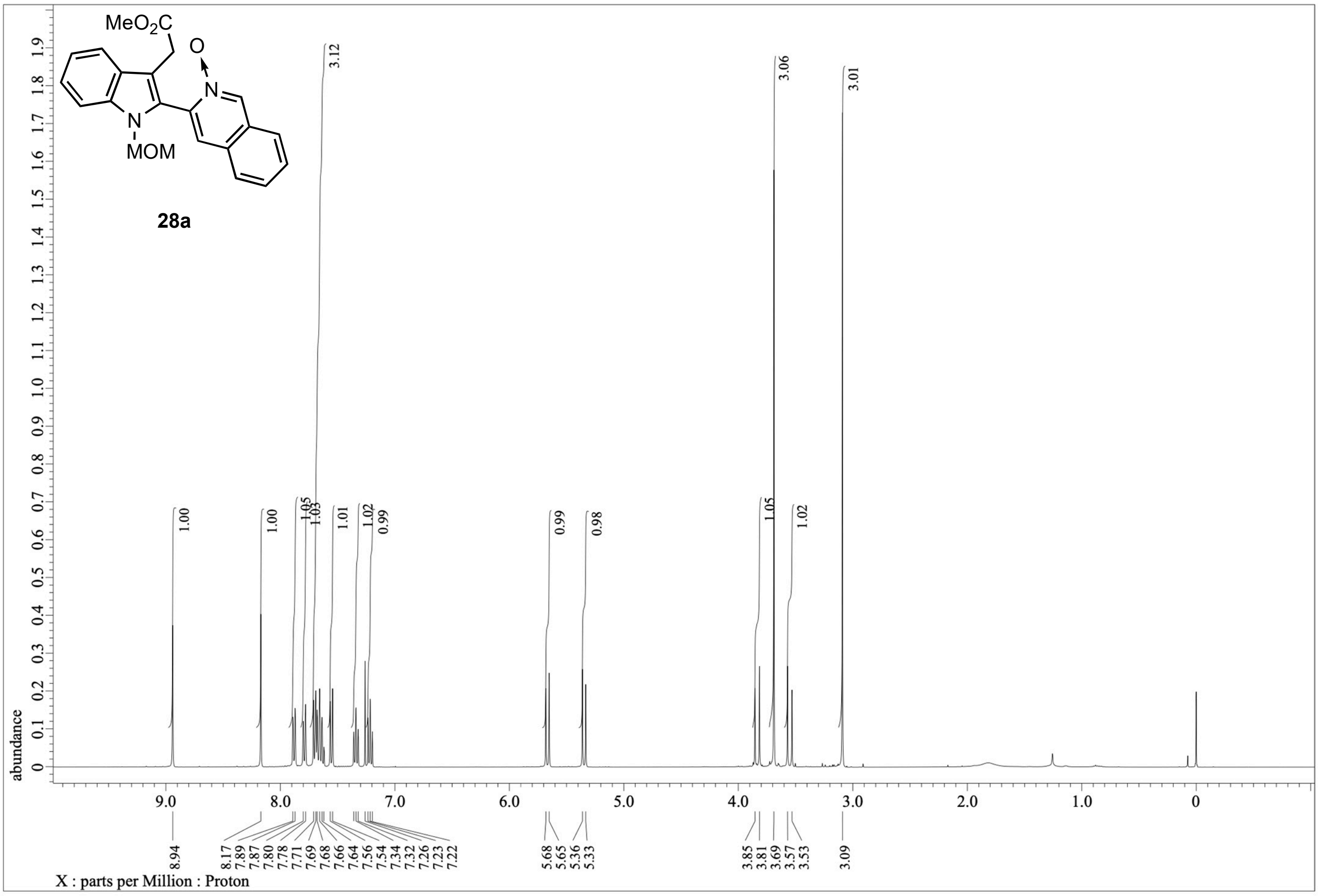

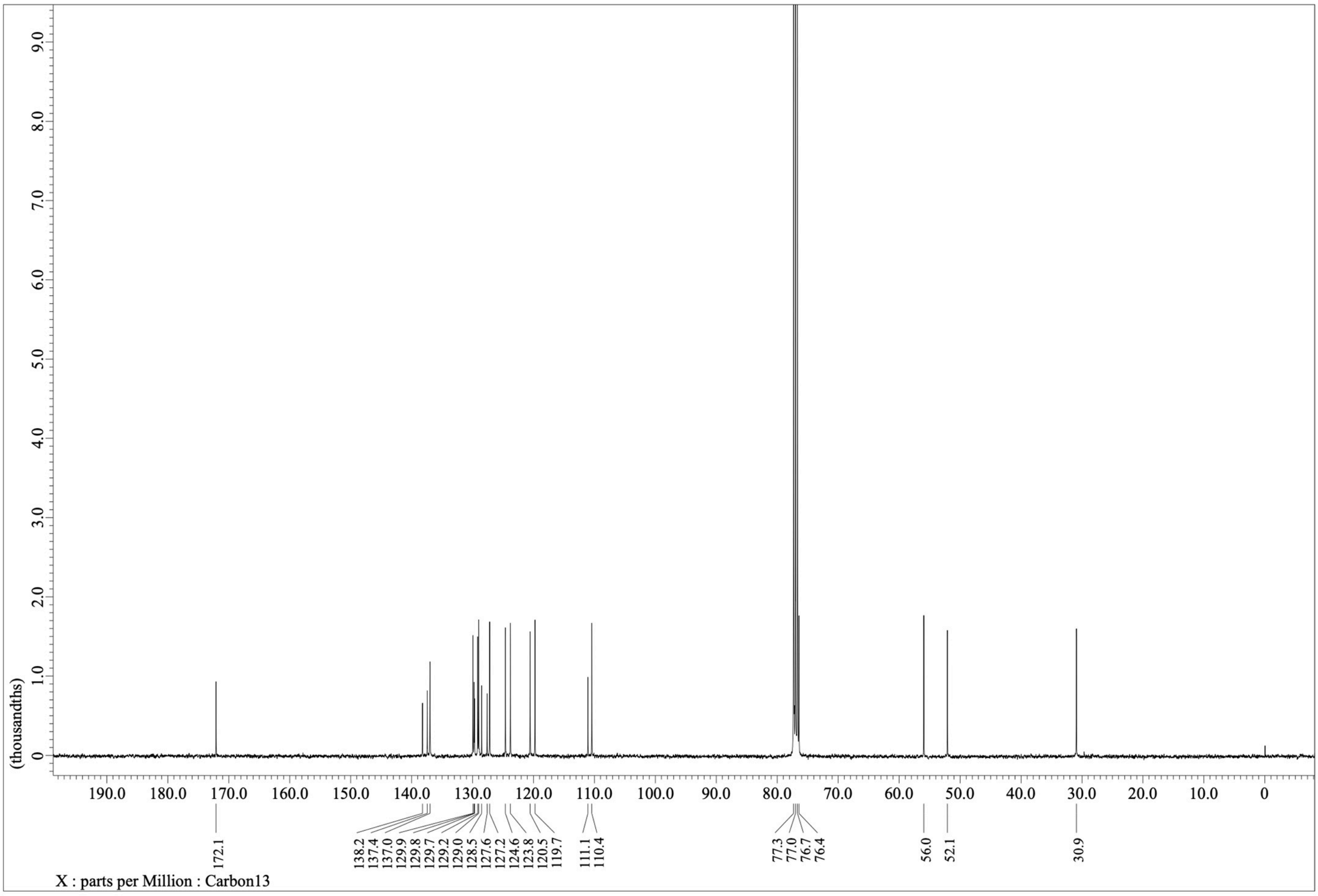

3.1.21. *3-[3-(2-Methoxy-2-oxoethyl)-1-(methoxymethyl)indol-2-yl)*

-5-[(methoxymethoxy)methyl]isoquinoline N-oxide (**28b**)

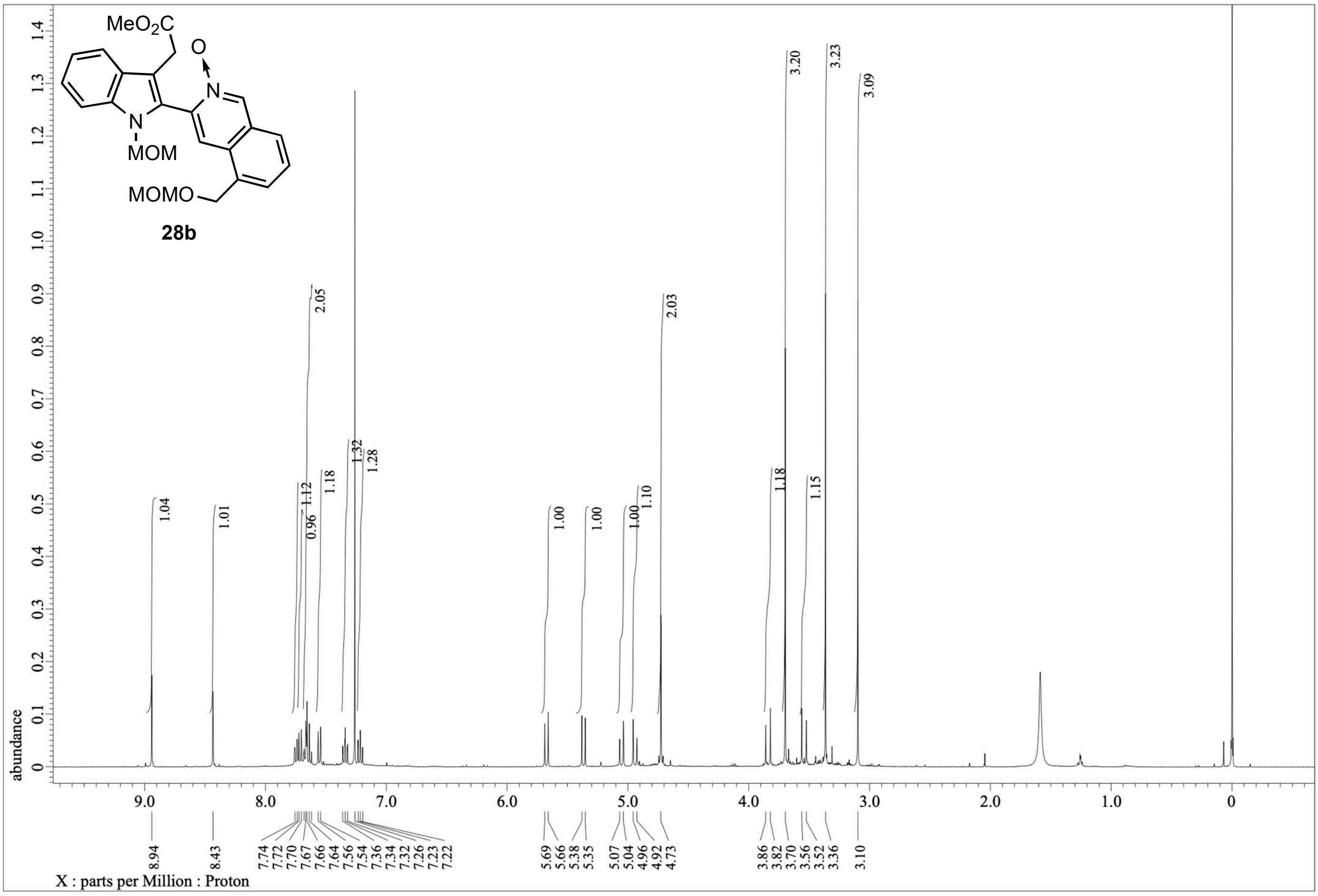

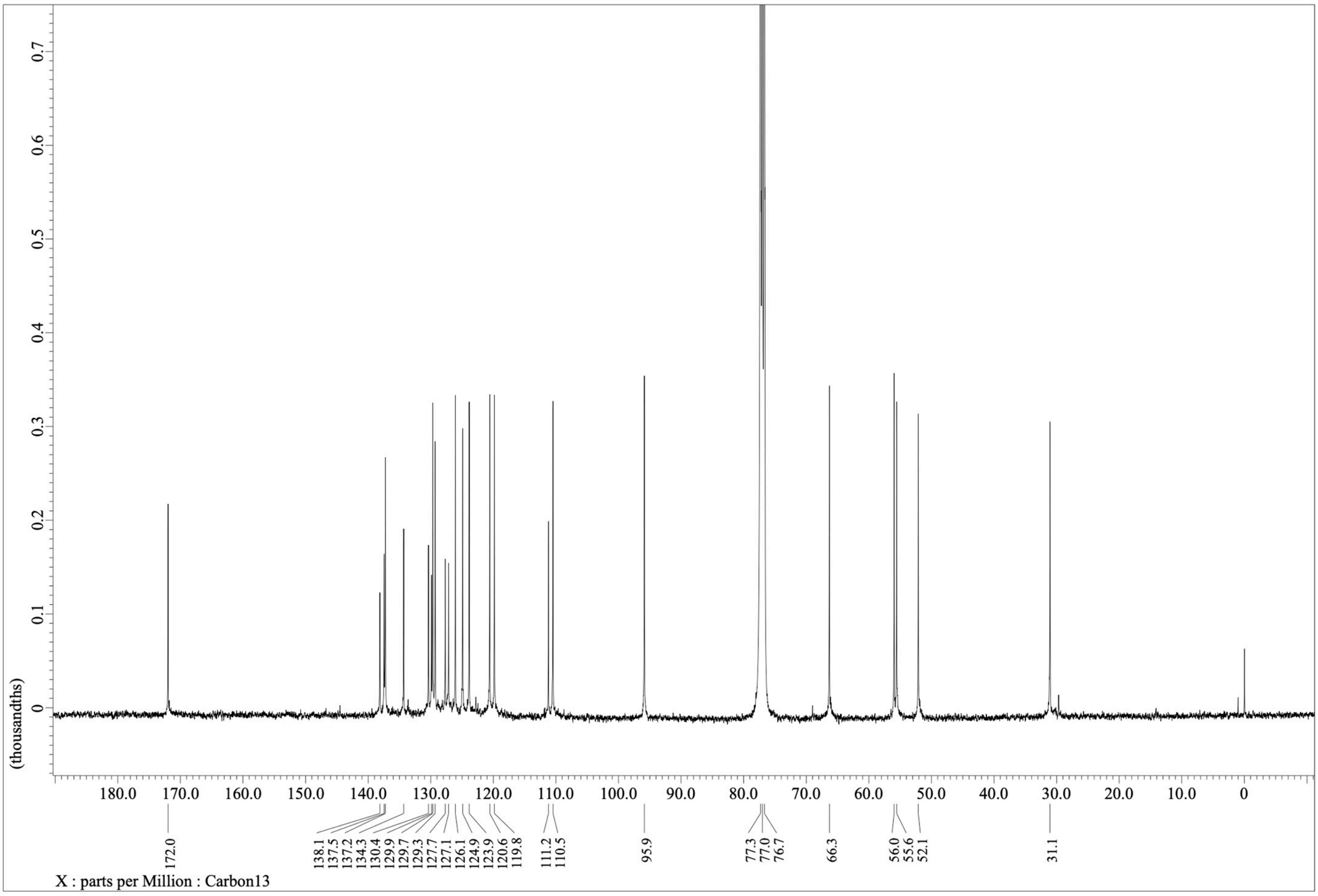

3.1.22. *Methyl 2-[1-(methoxymethyl)-2-(1-oxo-1,2-dihydroisoquinolin-3-yl)indol-3-yl]acetate* (**29a**)

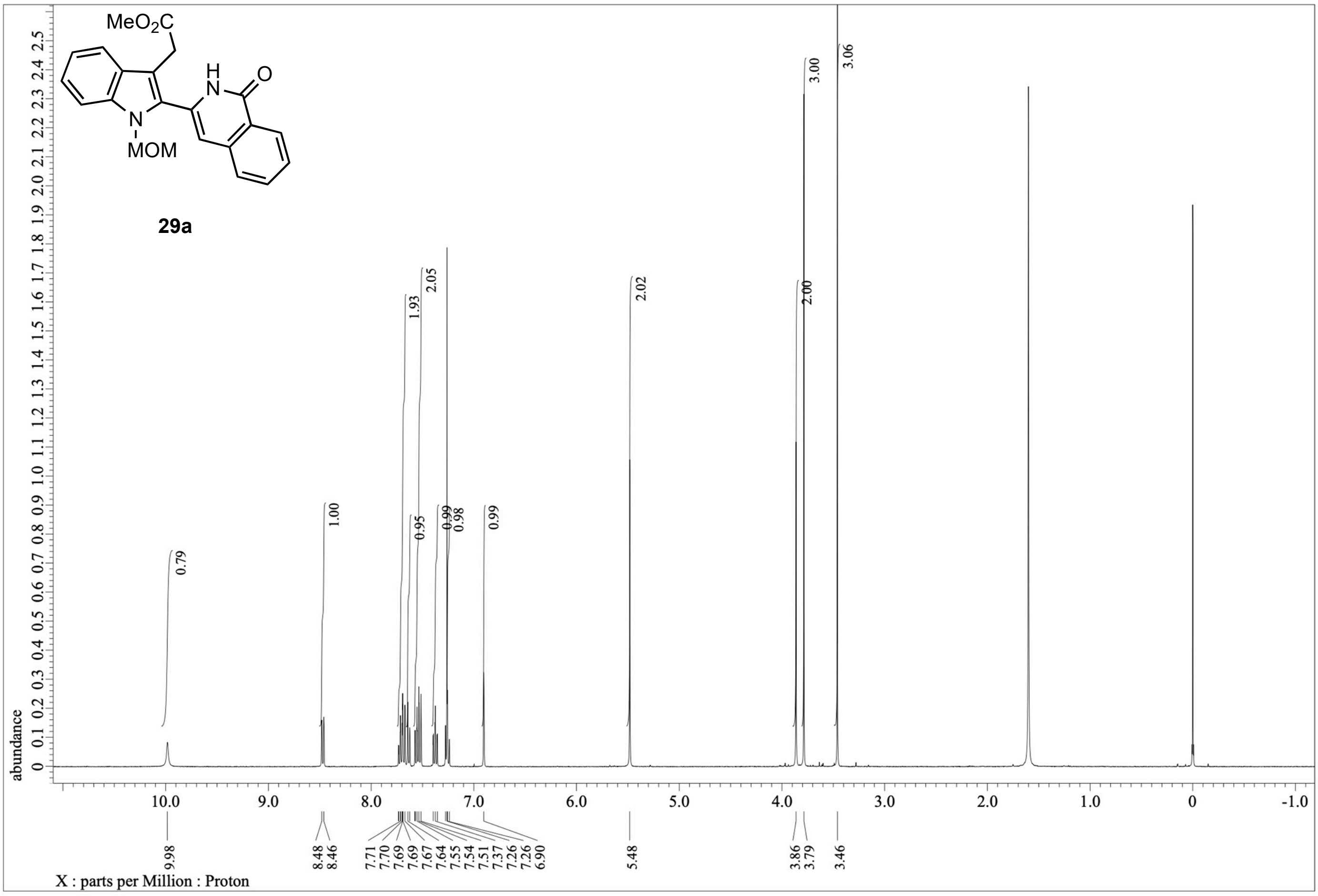
MeO2C
H
N
O
N
MOM
29a
abundance
0.79
1.00
0.95
1.93
2.05
0.99
0.98
0.99
2.02
2.00
3.00
3.06
10.0 9.0 8.0 7.0 6.0 5.0 4.0 3.0 2.0 1.0 0 -1.0
9.98
8.48
8.46
7.71
7.70
7.69
7.69
7.67
7.64
7.55
7.54
7.51
7.37
7.26
7.26
6.90
5.48
3.86
3.79
3.46
X : parts per Million : Proton

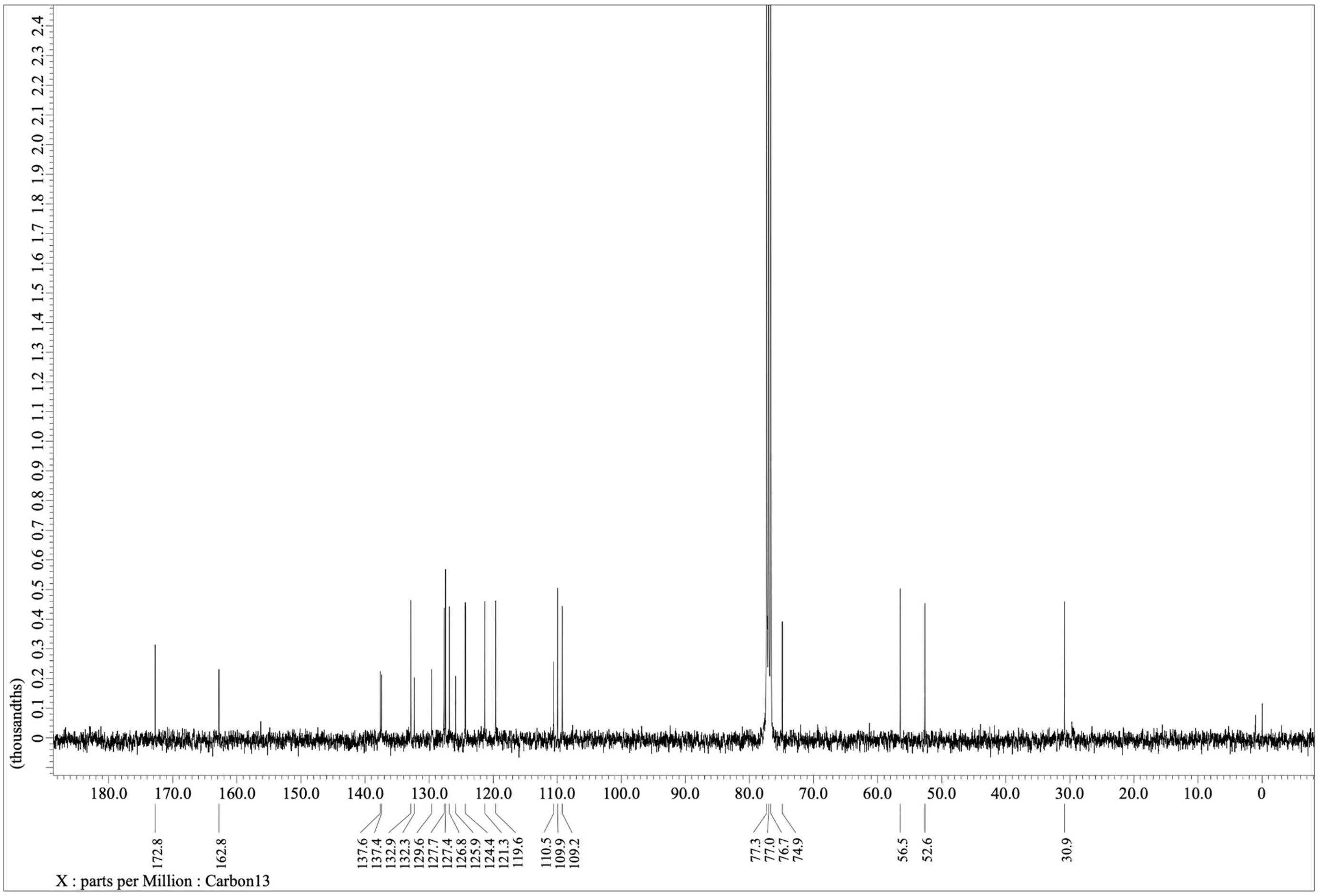
(thousandths)
180.0 170.0 160.0 150.0 140.0 130.0 120.0 110.0 100.0 90.0 80.0 70.0 60.0 50.0 40.0 30.0 20.0 10.0 0
172.8
162.8
137.6
137.4
132.9
132.3
129.6
127.7
127.4
126.8
125.9
124.4
121.3
119.6
110.5
109.9
109.2
77.3
77.0
76.7
74.9
56.5
52.6
30.9
X : parts per Million : Carbon13

***3.1.22.** Methyl 2-[2-(4-acetoxyisoquinolin-3-yl)-1-(methoxymethyl)indol-3-yl]acetate* (**31a**)

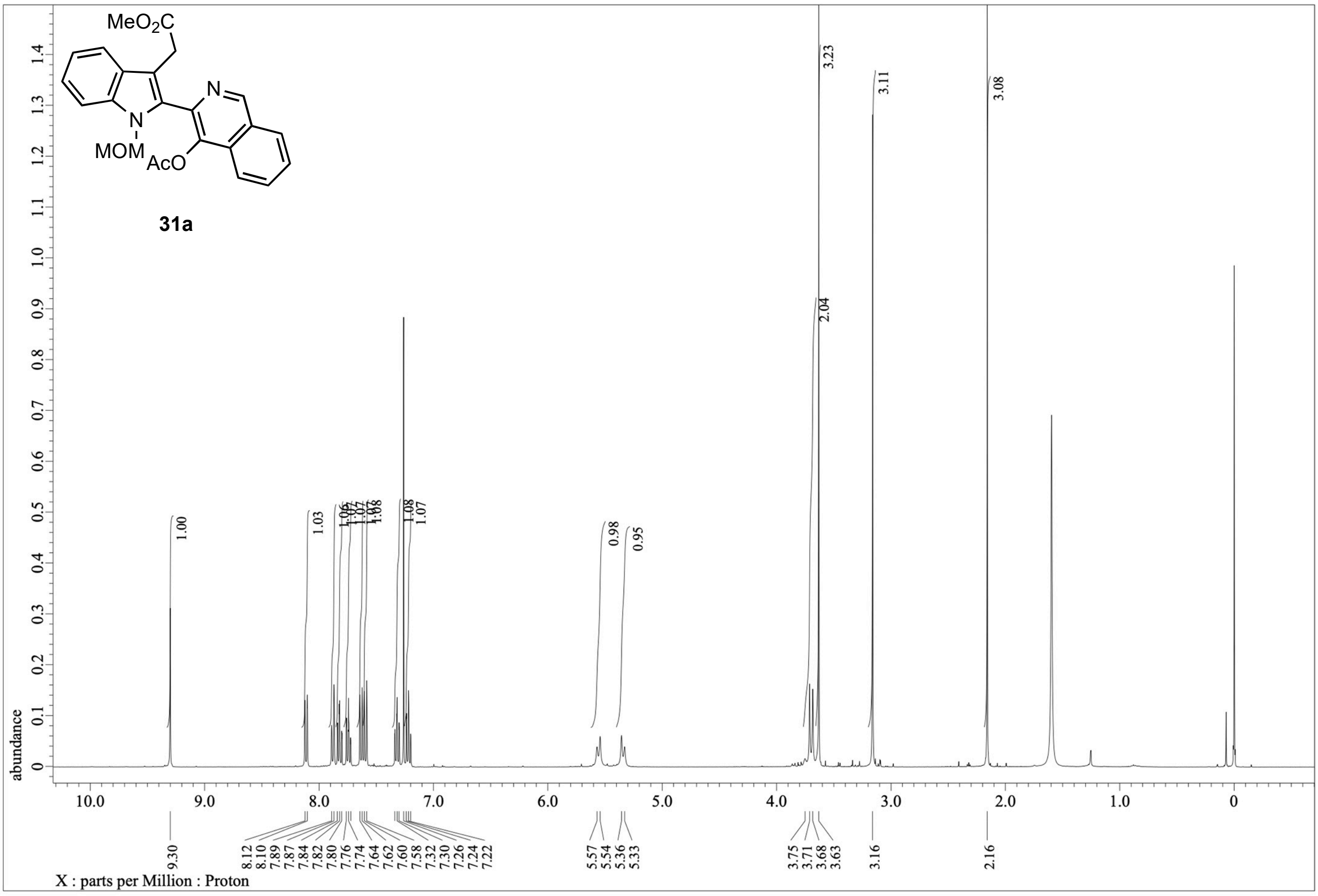

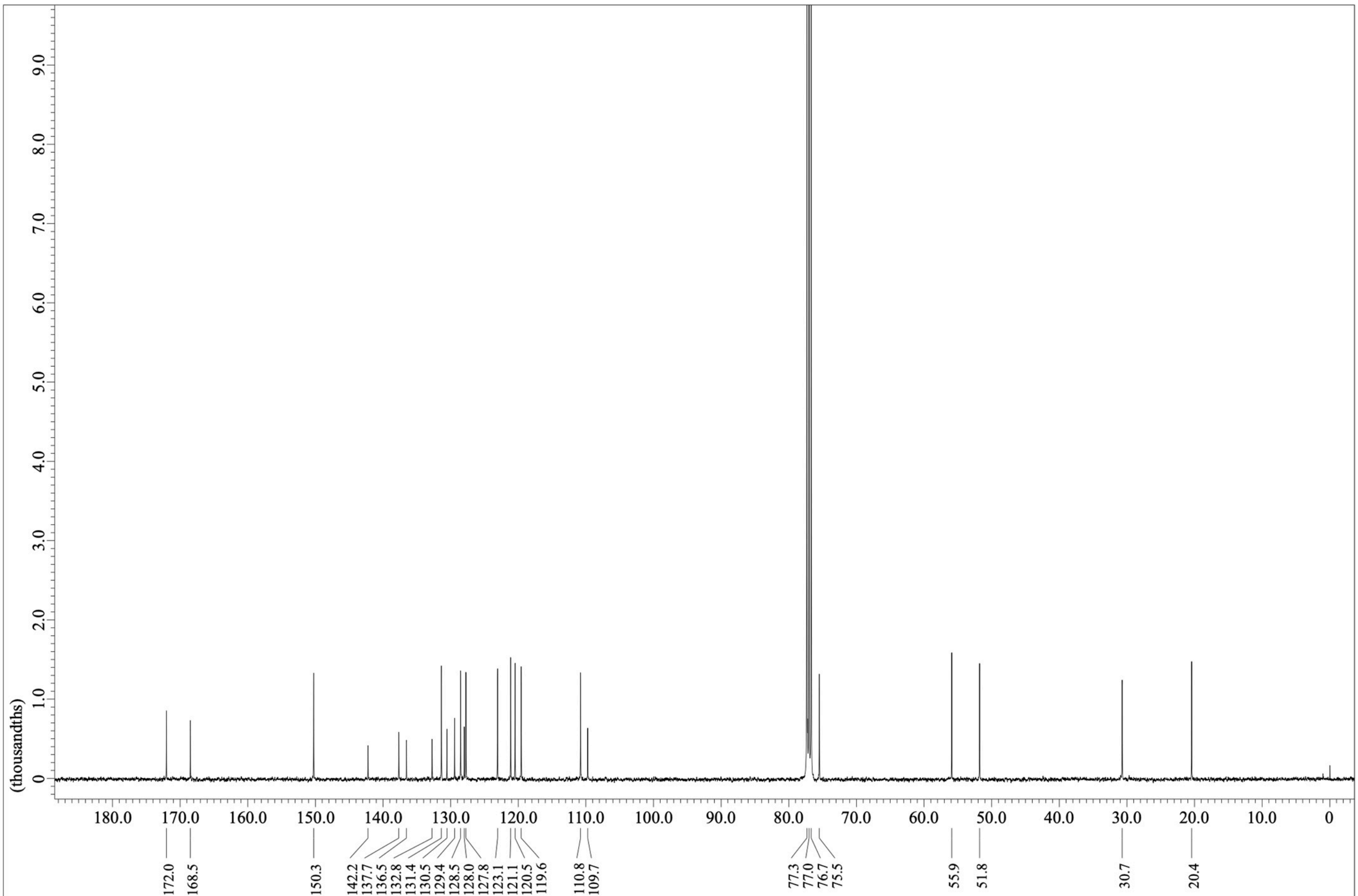

***3.1.23.** Methyl 2-{2-[5-[(methoxymethoxy)methyl]-1-oxo-1,2-dihydroisoquinolin-3-yl]*

-1-(methoxymethyl)indol-3-yl}acetate (**29b**)

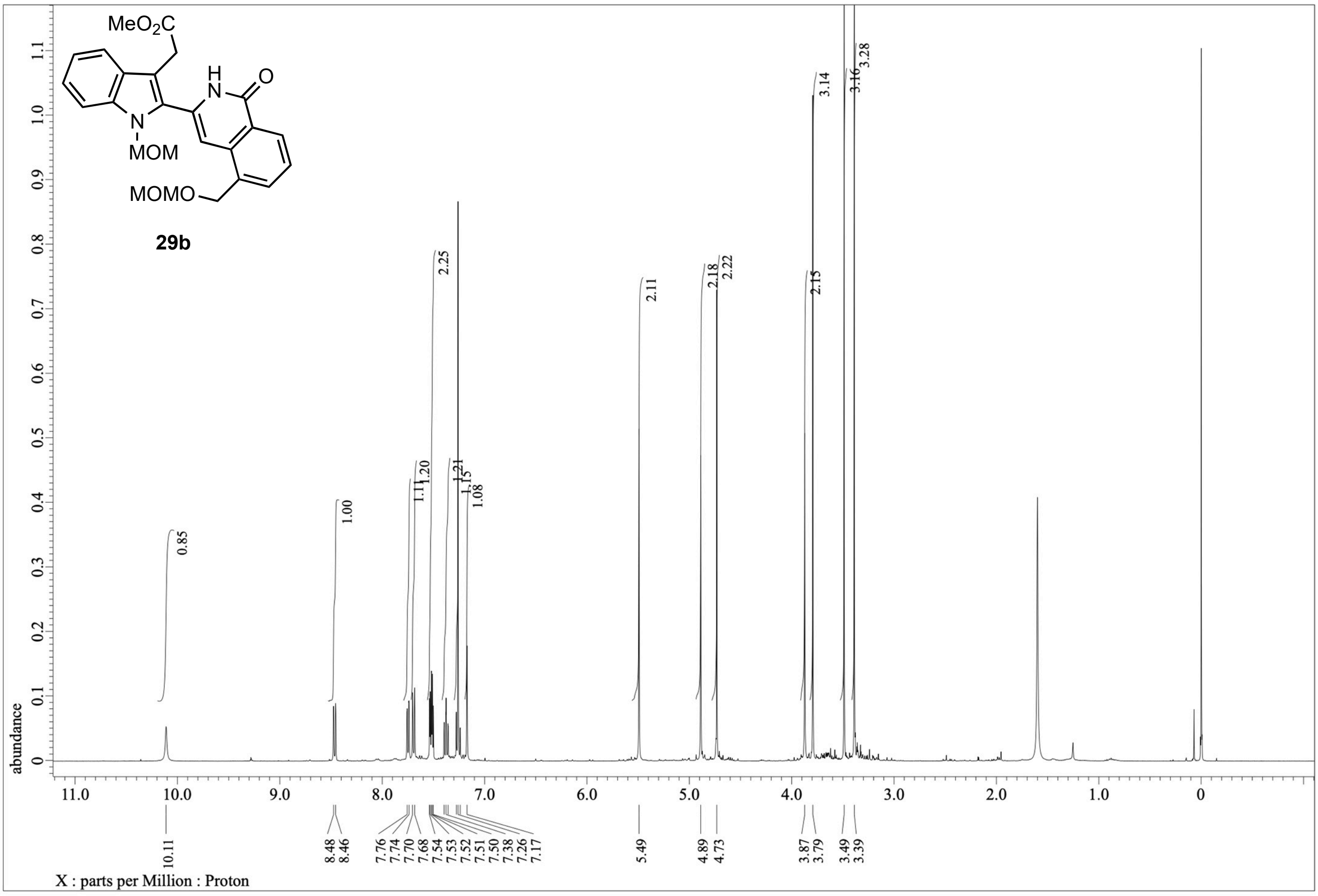

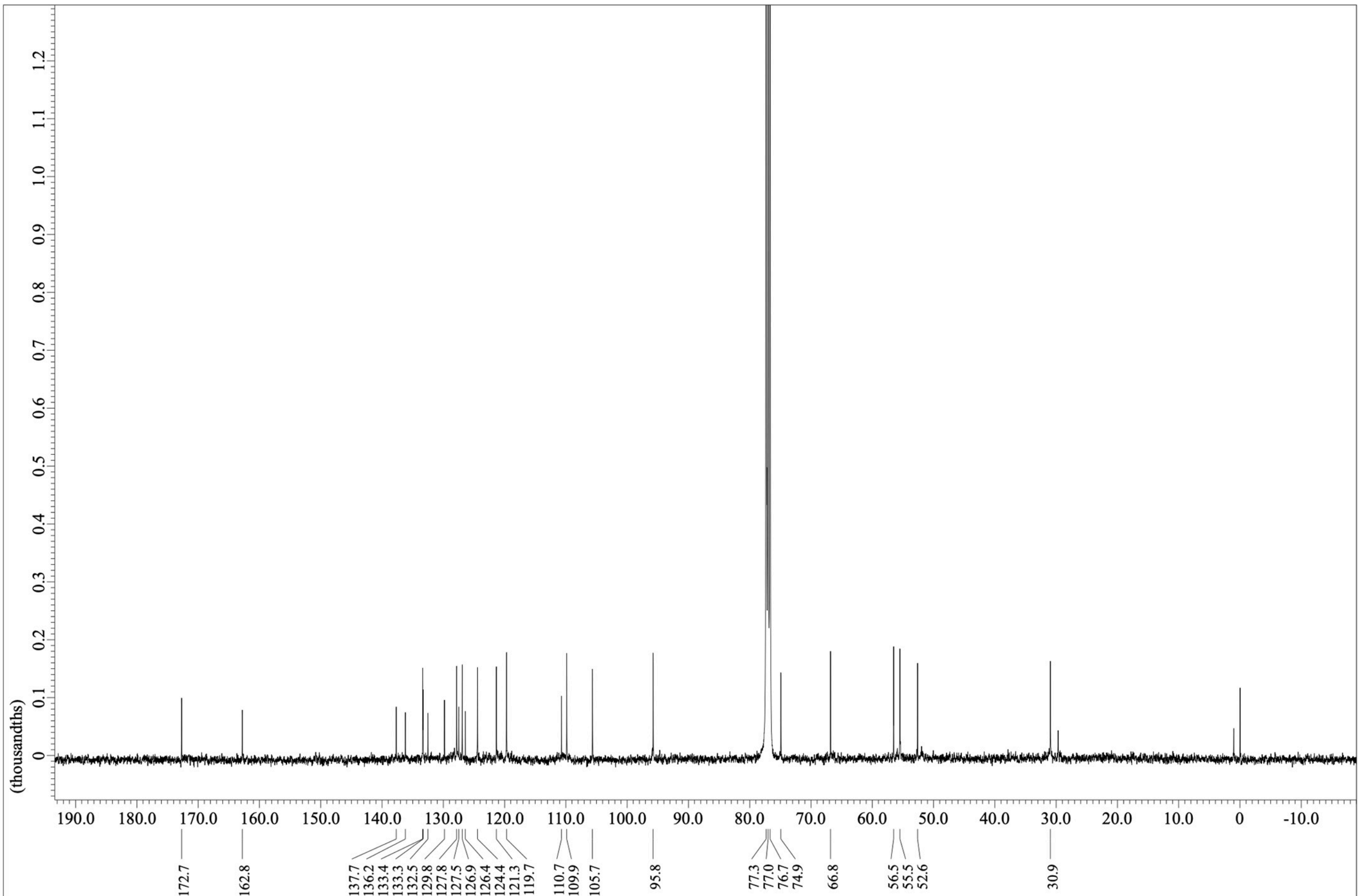

3.1.23. *1-Acetoxy-3-[3-methoxycarbonylmethyl-N-(methoxymethyl)indol-2-yl]*

-5-[(methoxymethoxy)methyl]isoquinoline (**30b**)

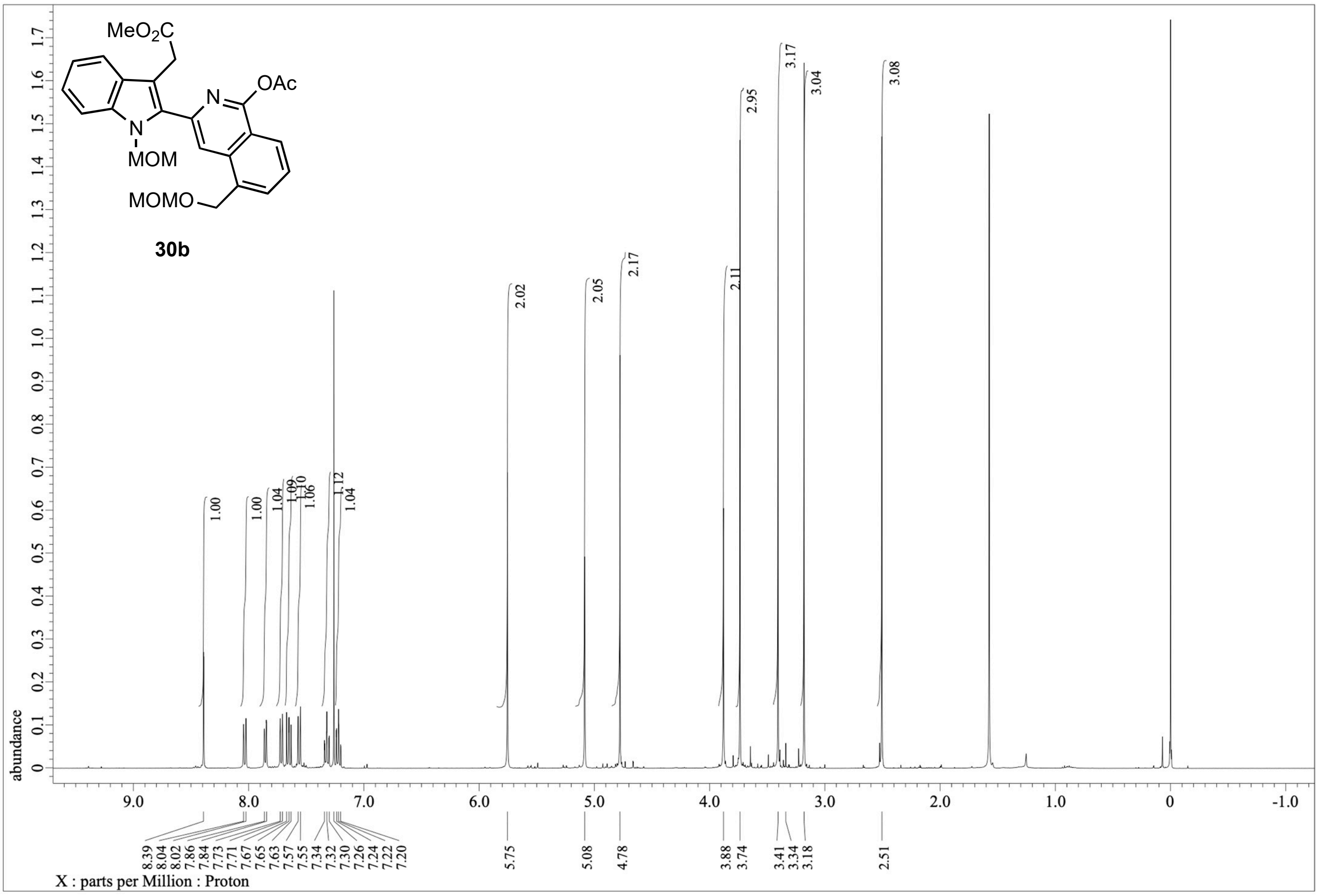

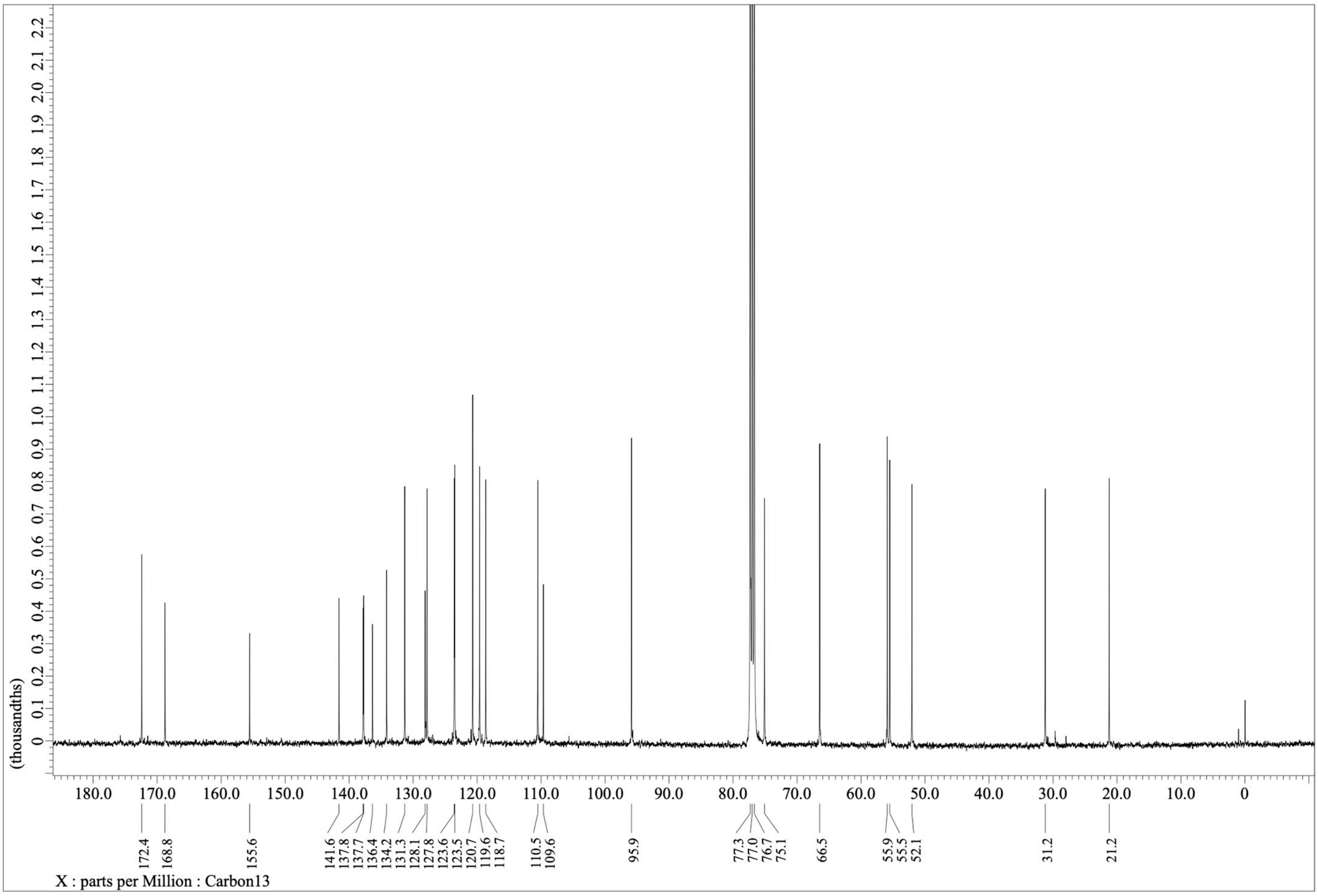

3.1.23. *4-Acetoxy-3-[3-methoxycarbonylmethyl-N-(methoxymethyl)indol-2-yl]*

-5-[(methoxymethoxy)methyl]isoquinoline (**31b**)

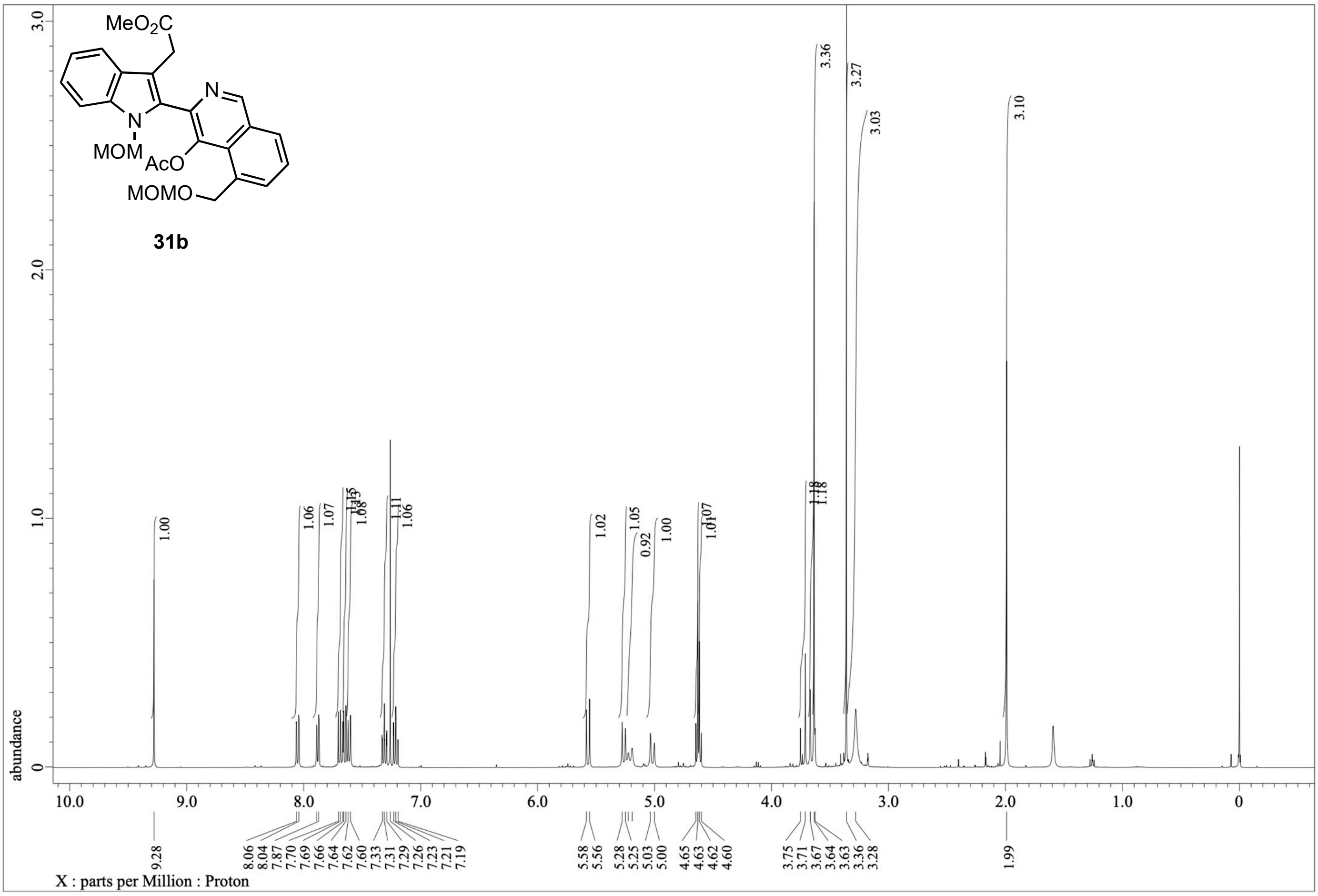

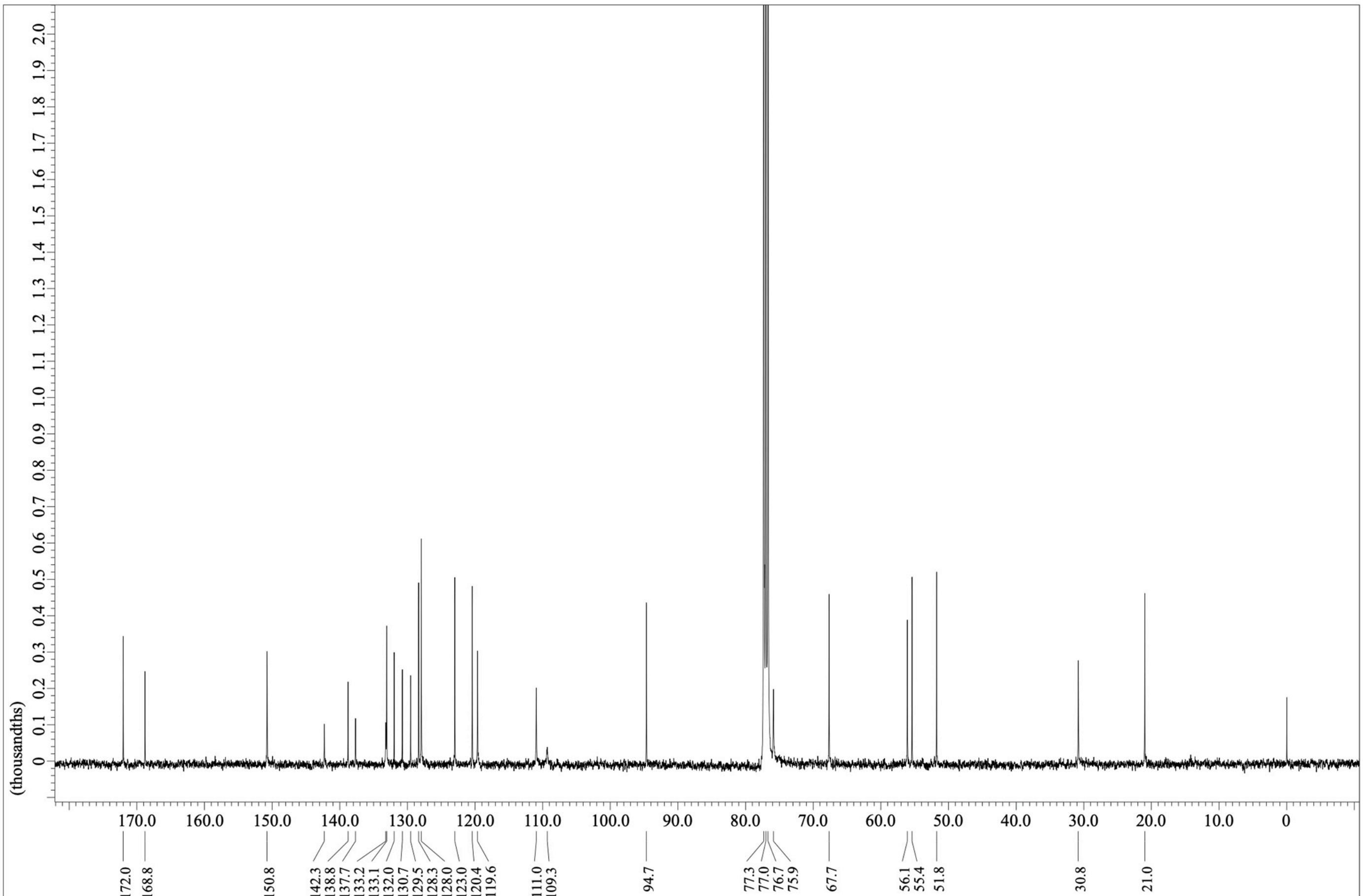

3.1.24. *3-[3-(2-Hydroxyethyl)-N-(methoxymethyl)indol-2-yl]isoquinolin-1-one* (**32a**)

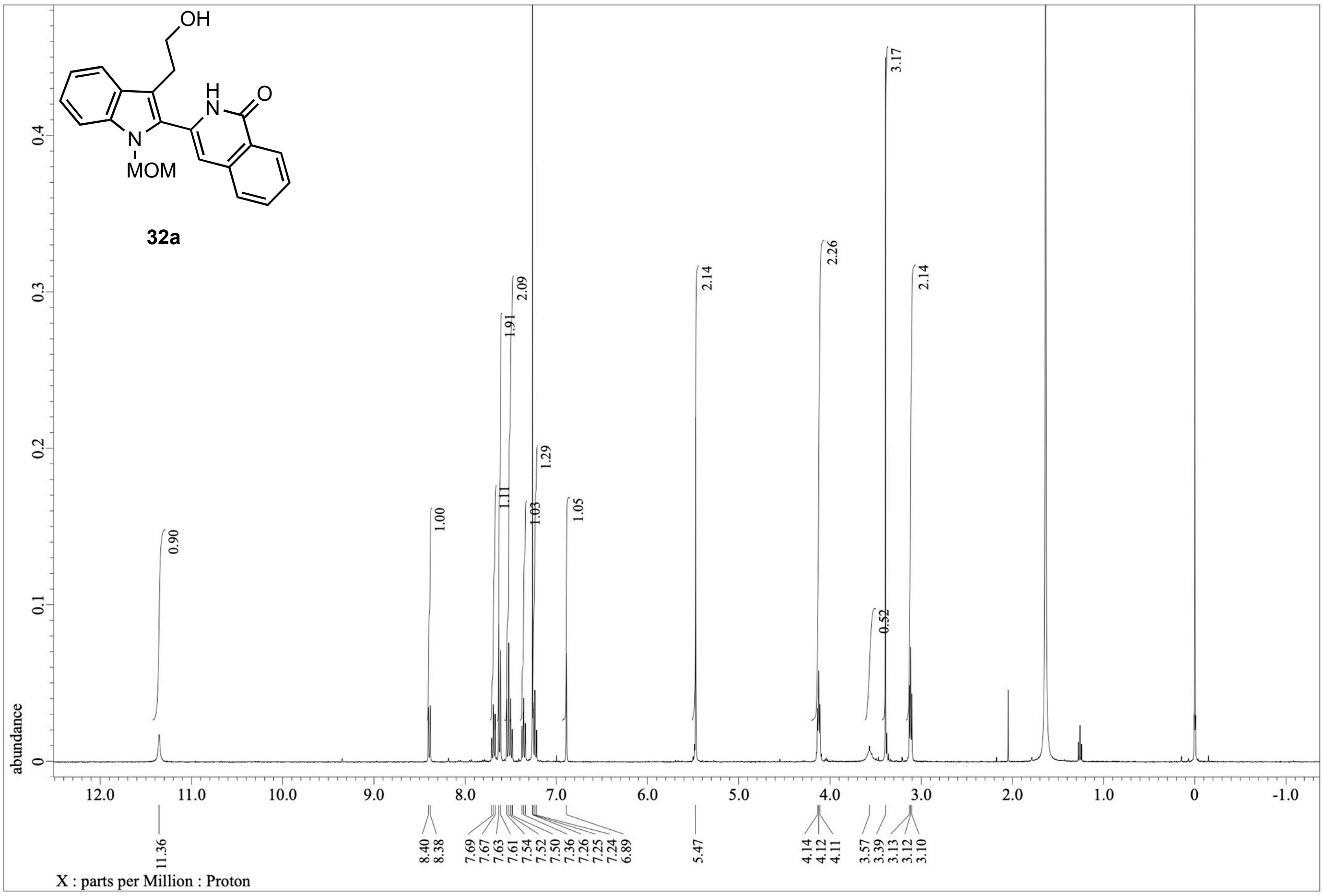

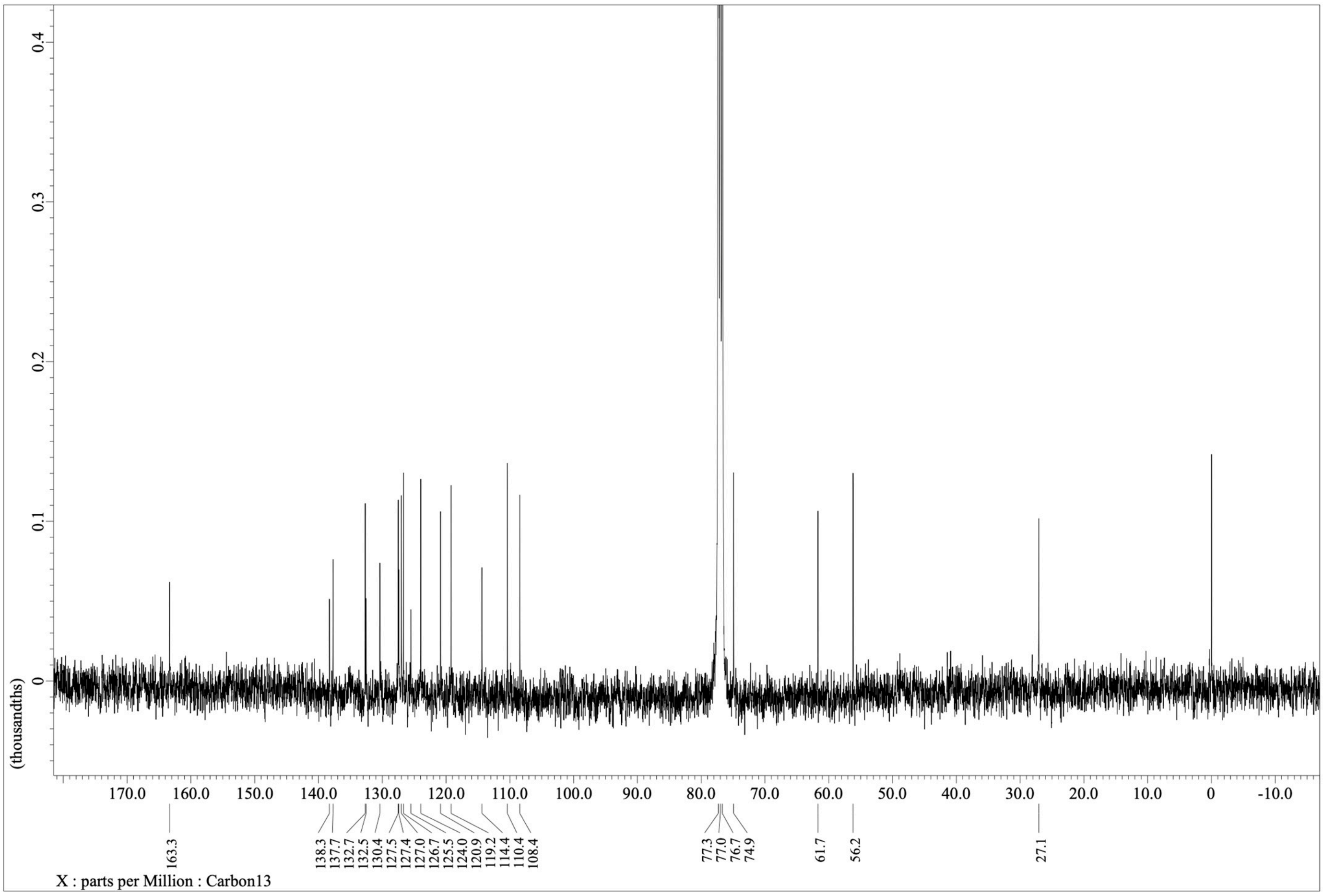

3.1.25. *3-[3-(2-Hydroxyethyl)-N-(methoxymethyl)indol-2-yl]*

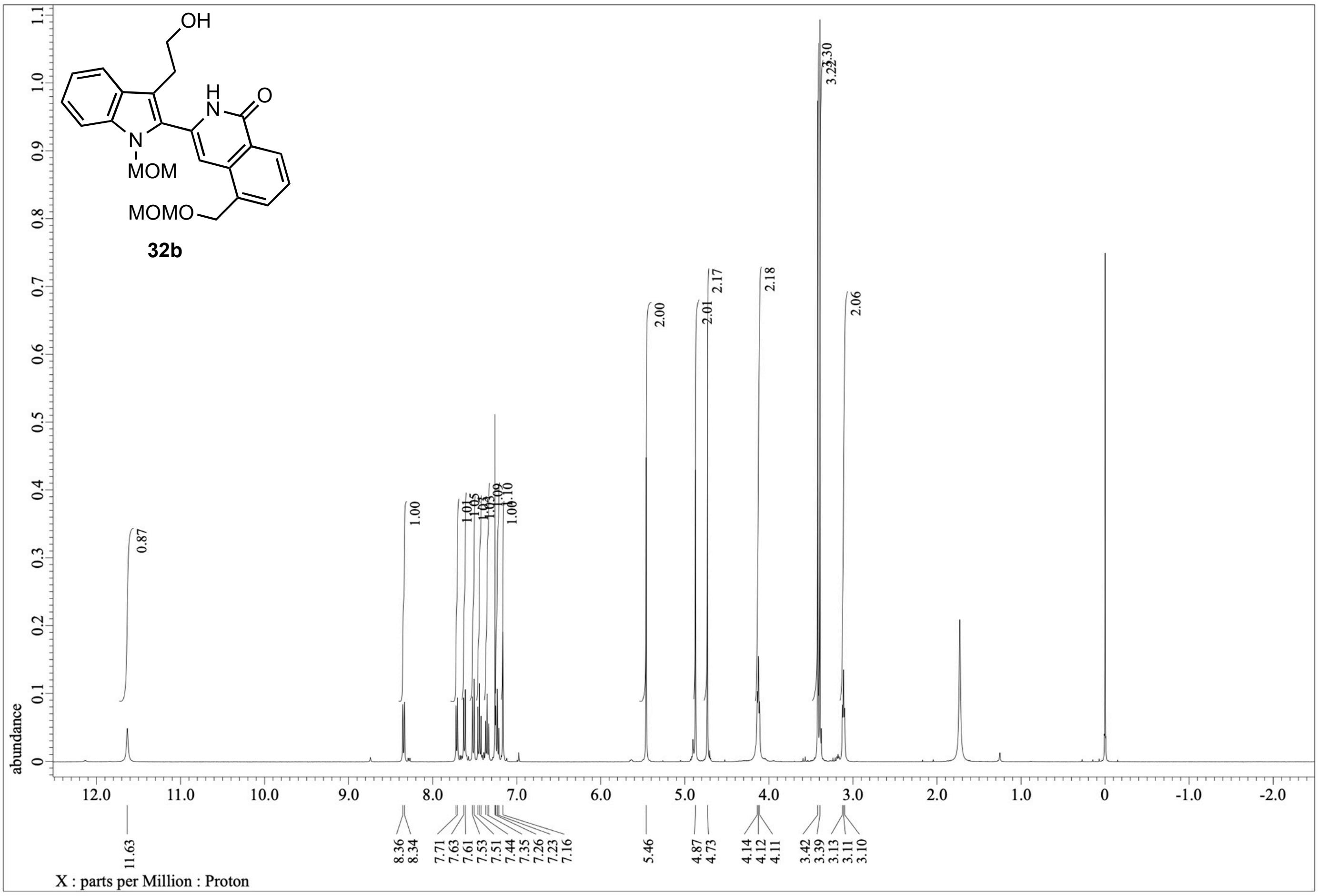

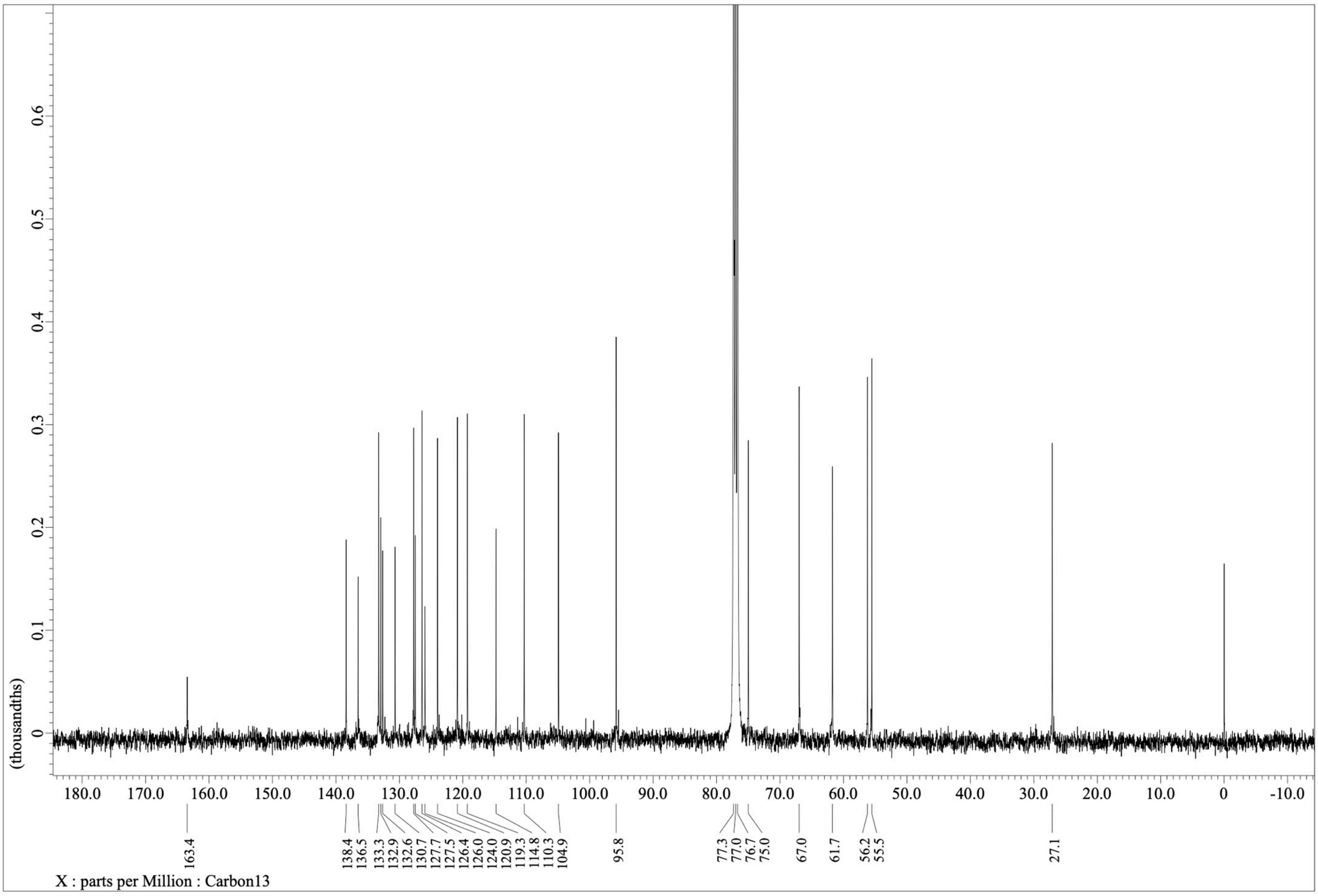

3.1.26. *N-Methoxymethyl-3,14,15,16,17,18,19,20-octadehydroyohimban-21-one* (**33a**)

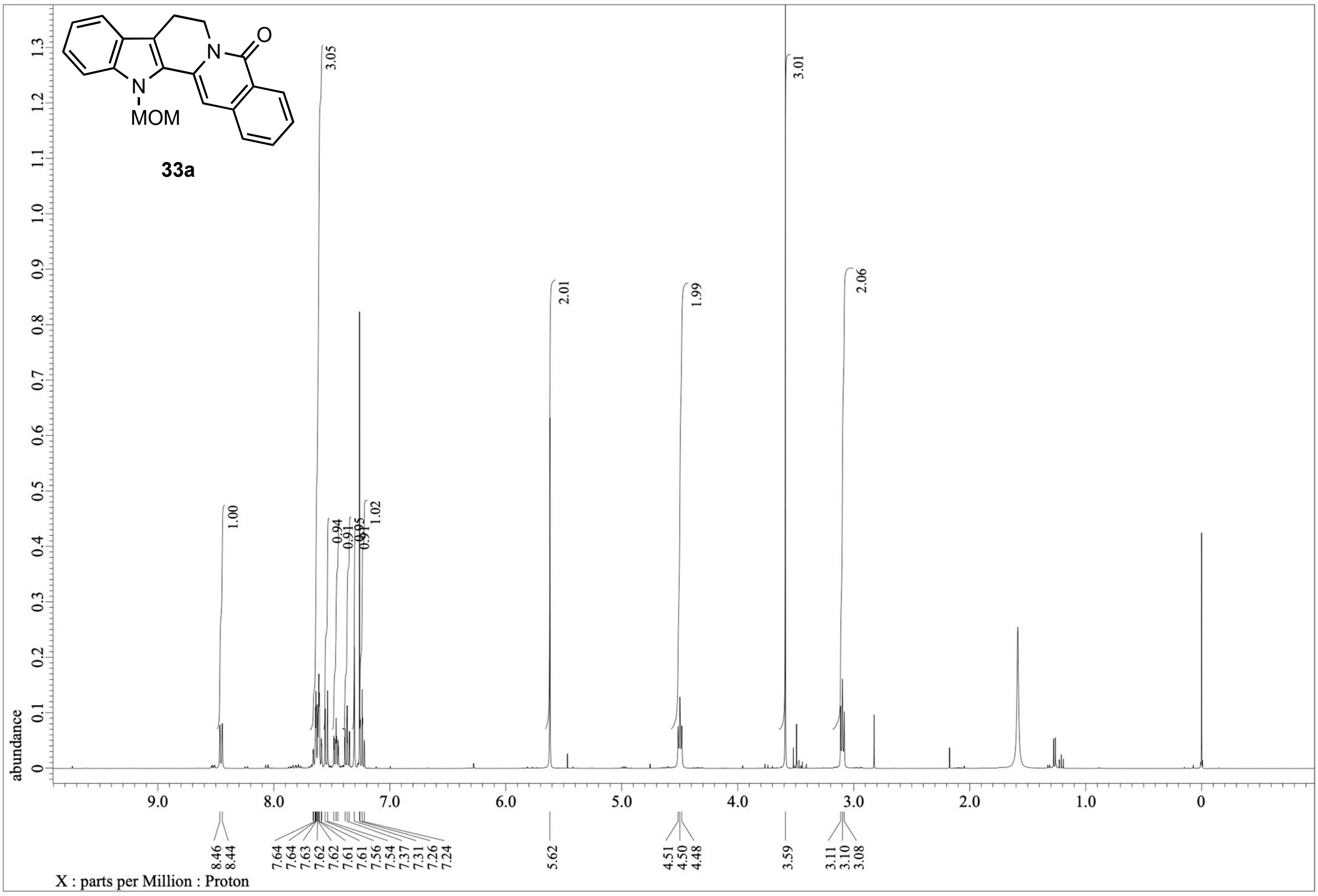

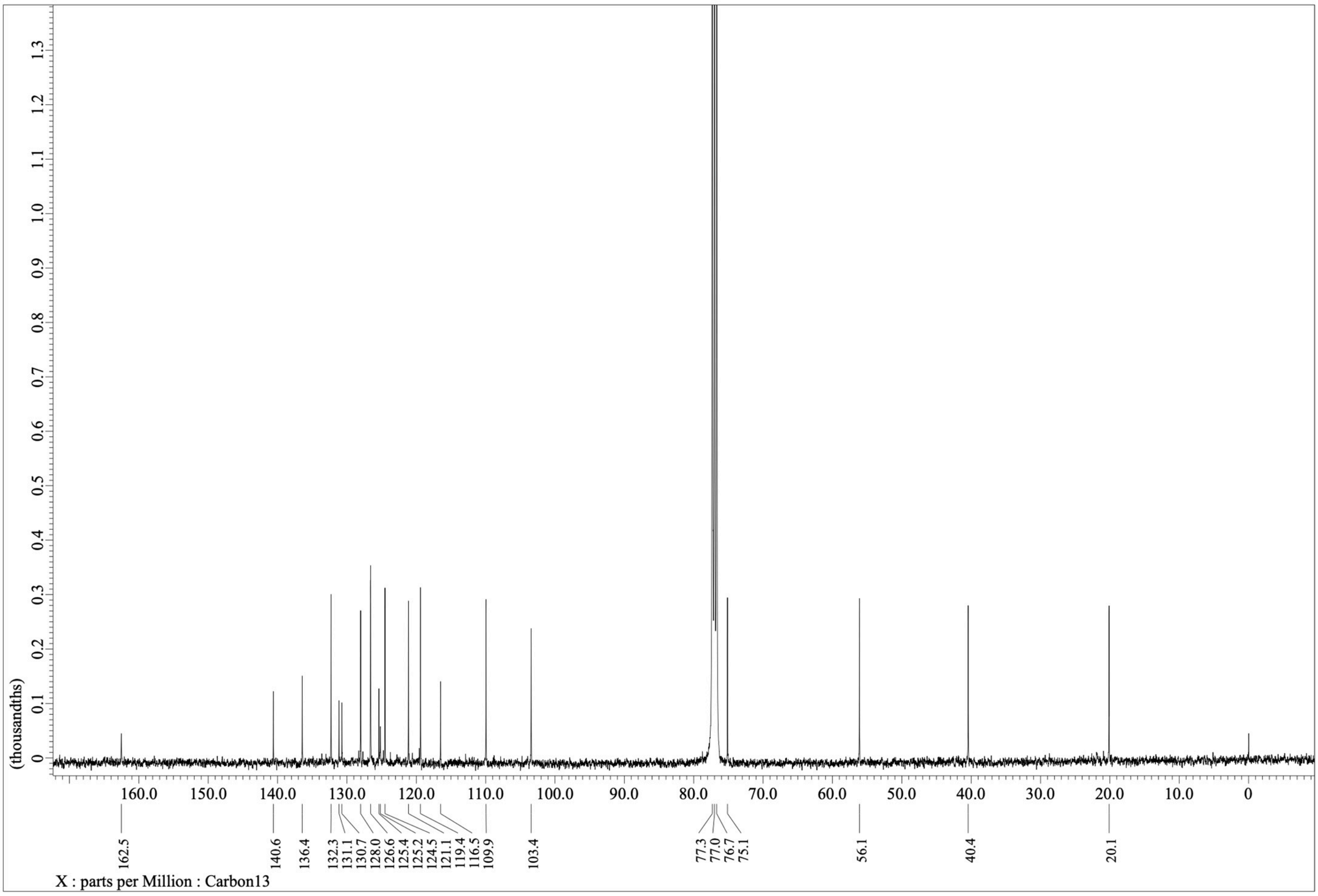

3.1.27. *N-Methoxymethyl-16-[(methoxymethoxy)methyl]*

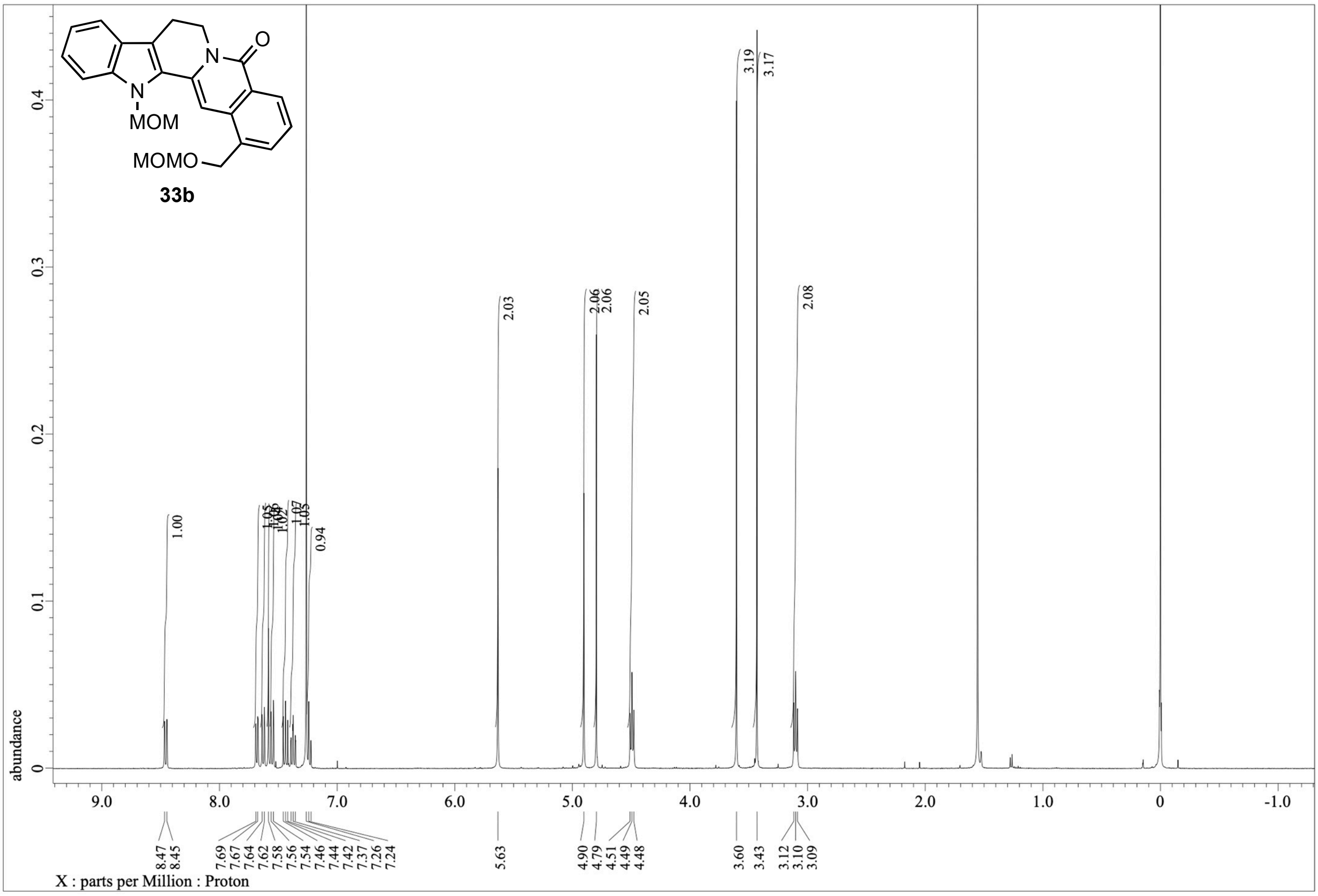

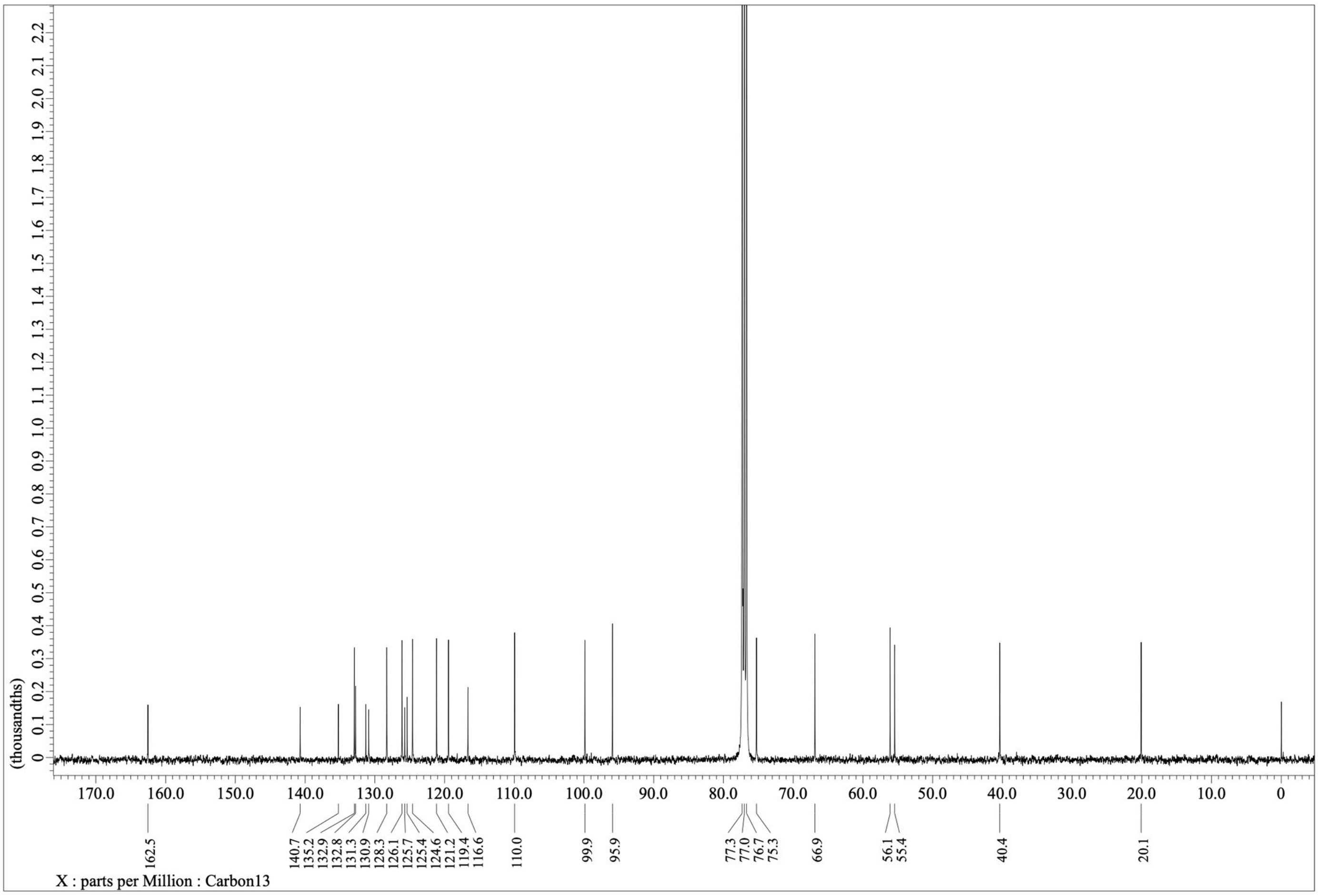

3.1.28. *Norketoyobyrine* (**5**)

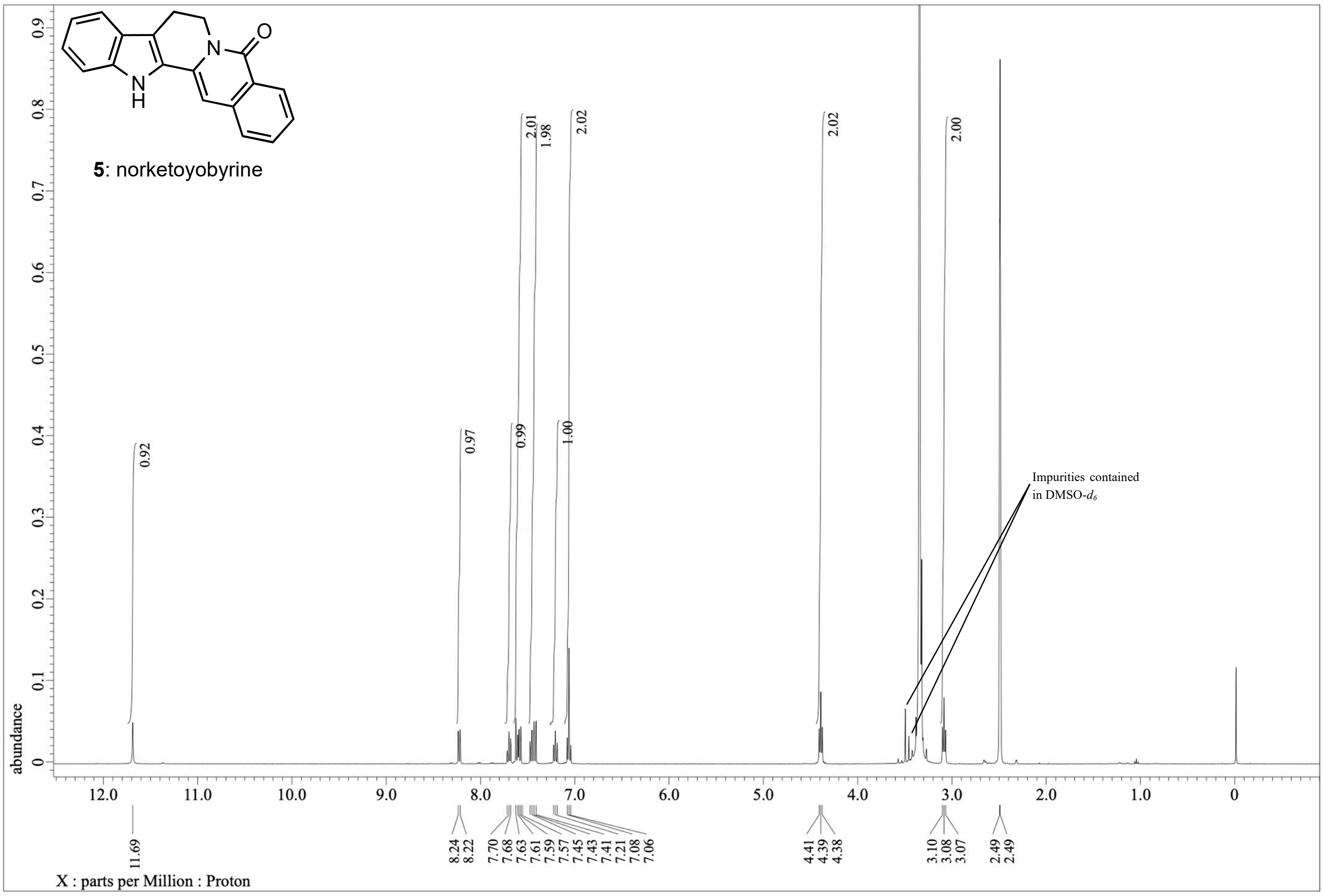

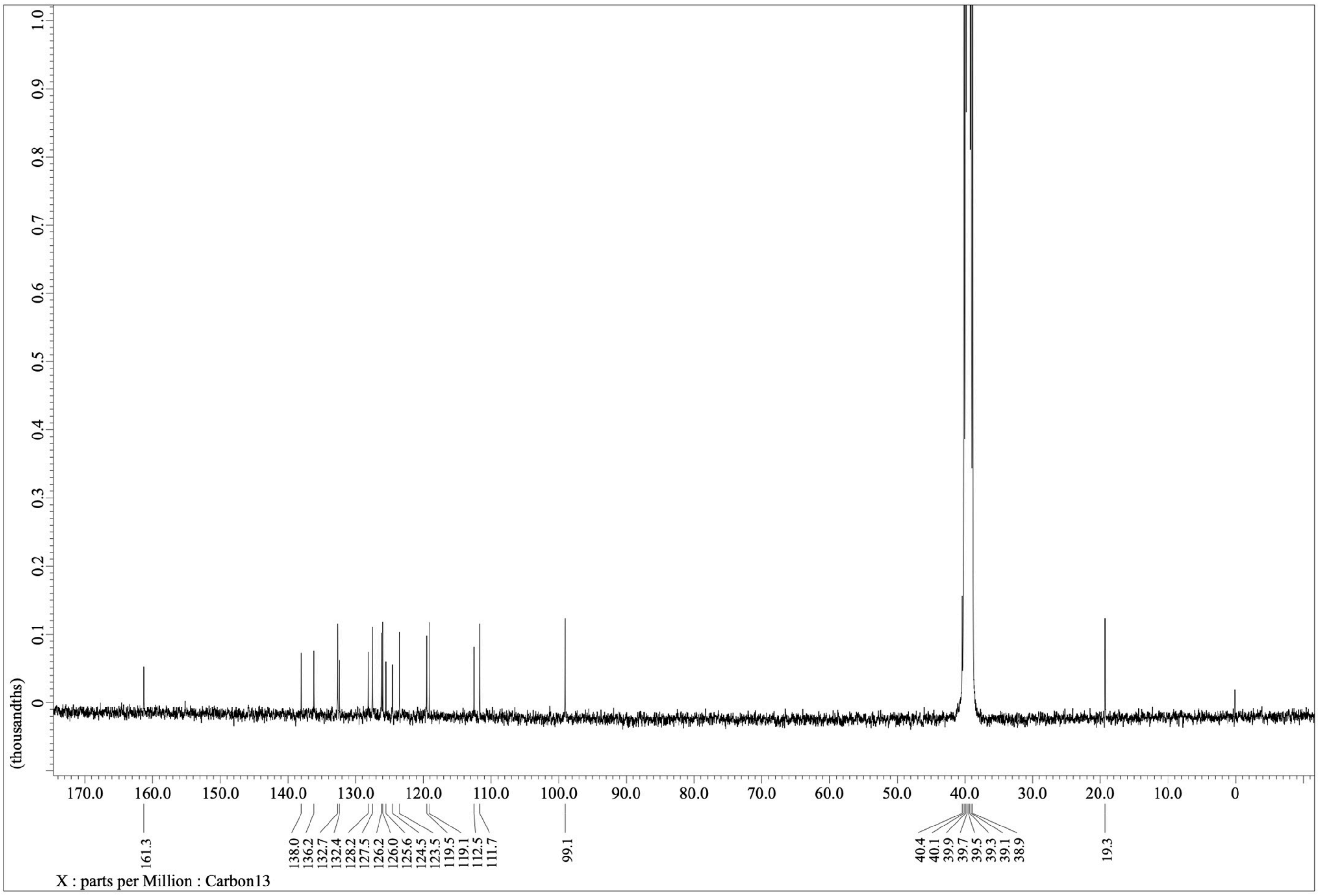

3.1.29. *16-Hydroxymethyl-3,14,15,16,17,18,19,20-octadehydroyohimban-21-one* (**34**)

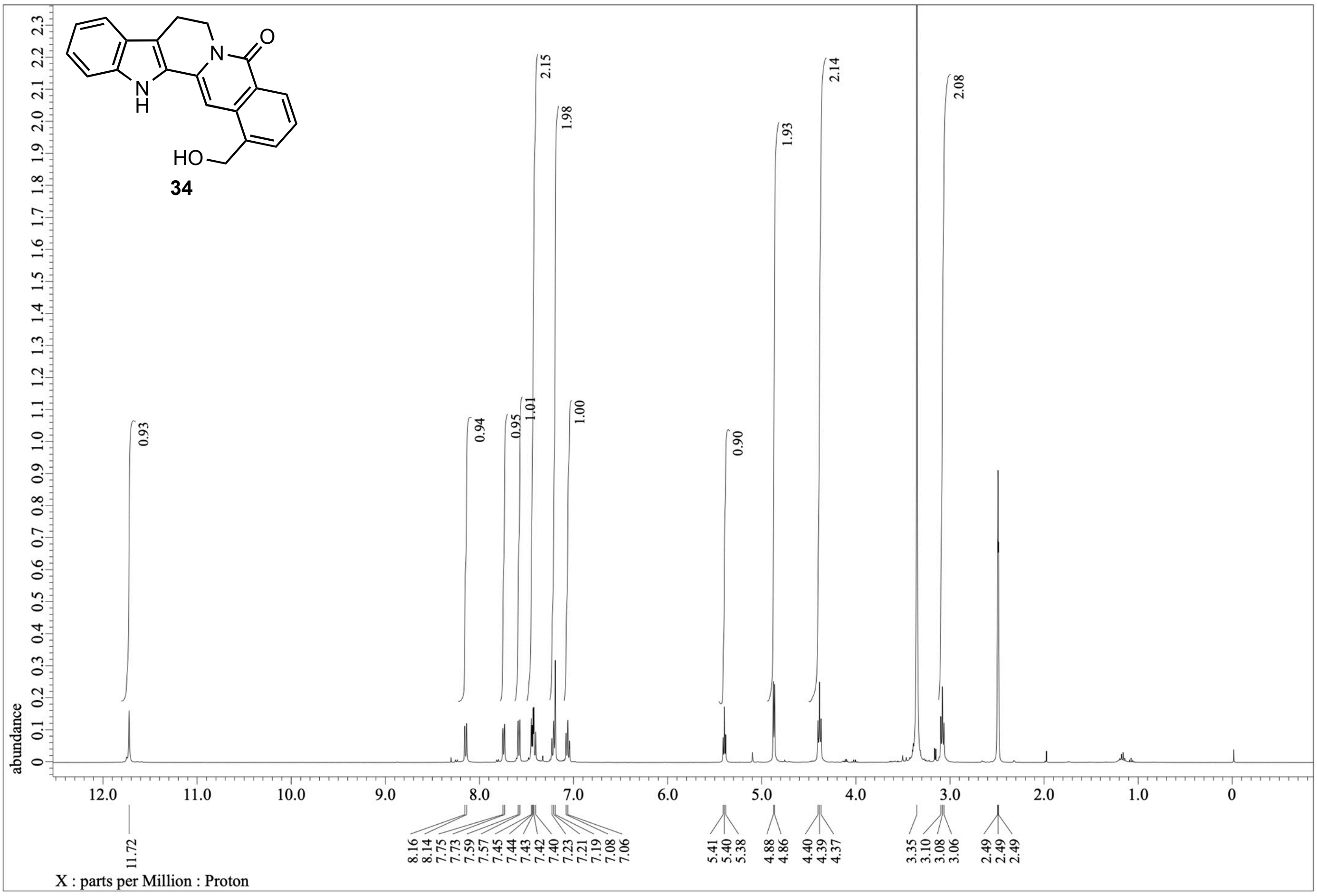

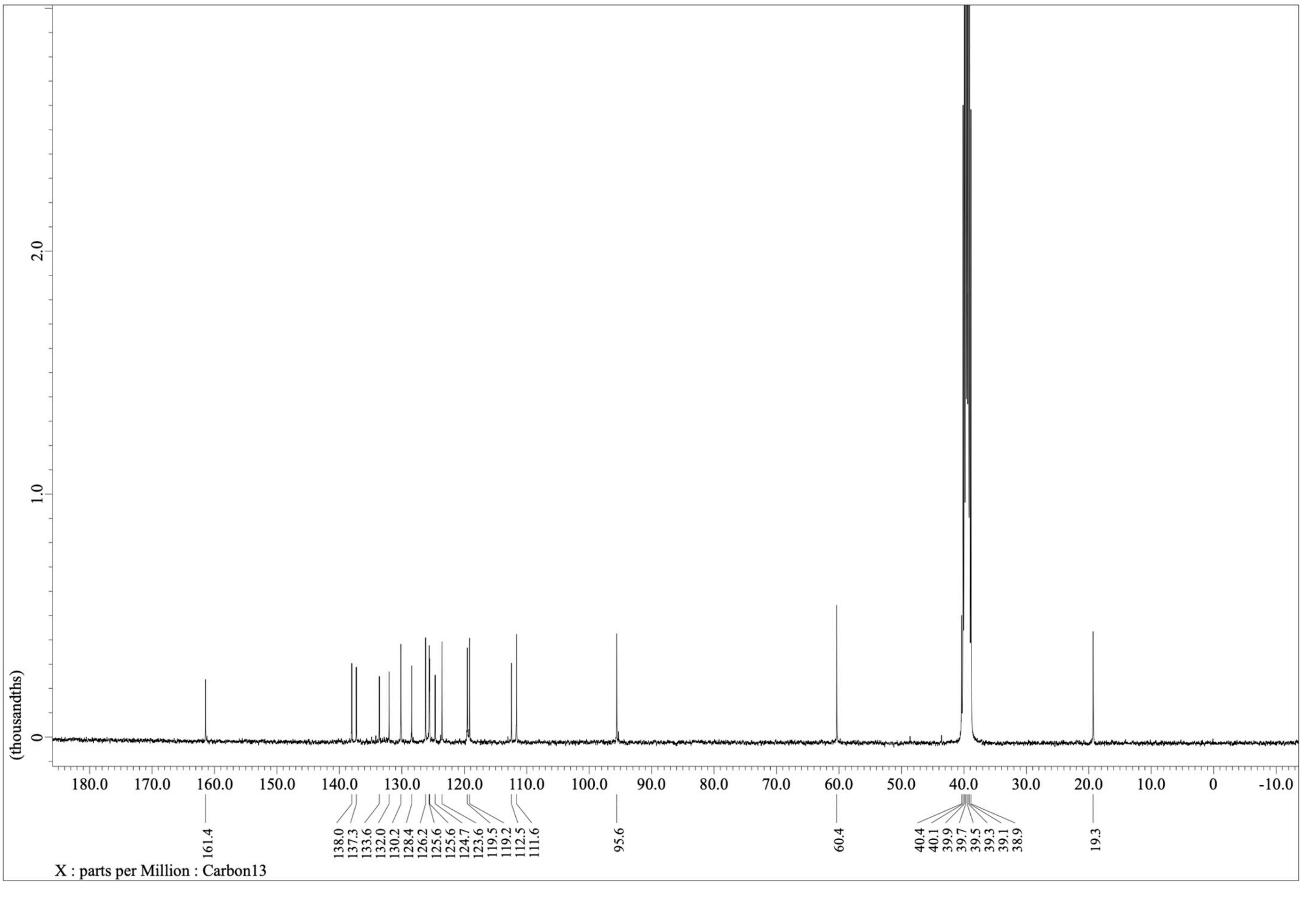

3.1.30. *Naucleficine* (**7**) ^{1}H-NMR (DMSO-d_6)

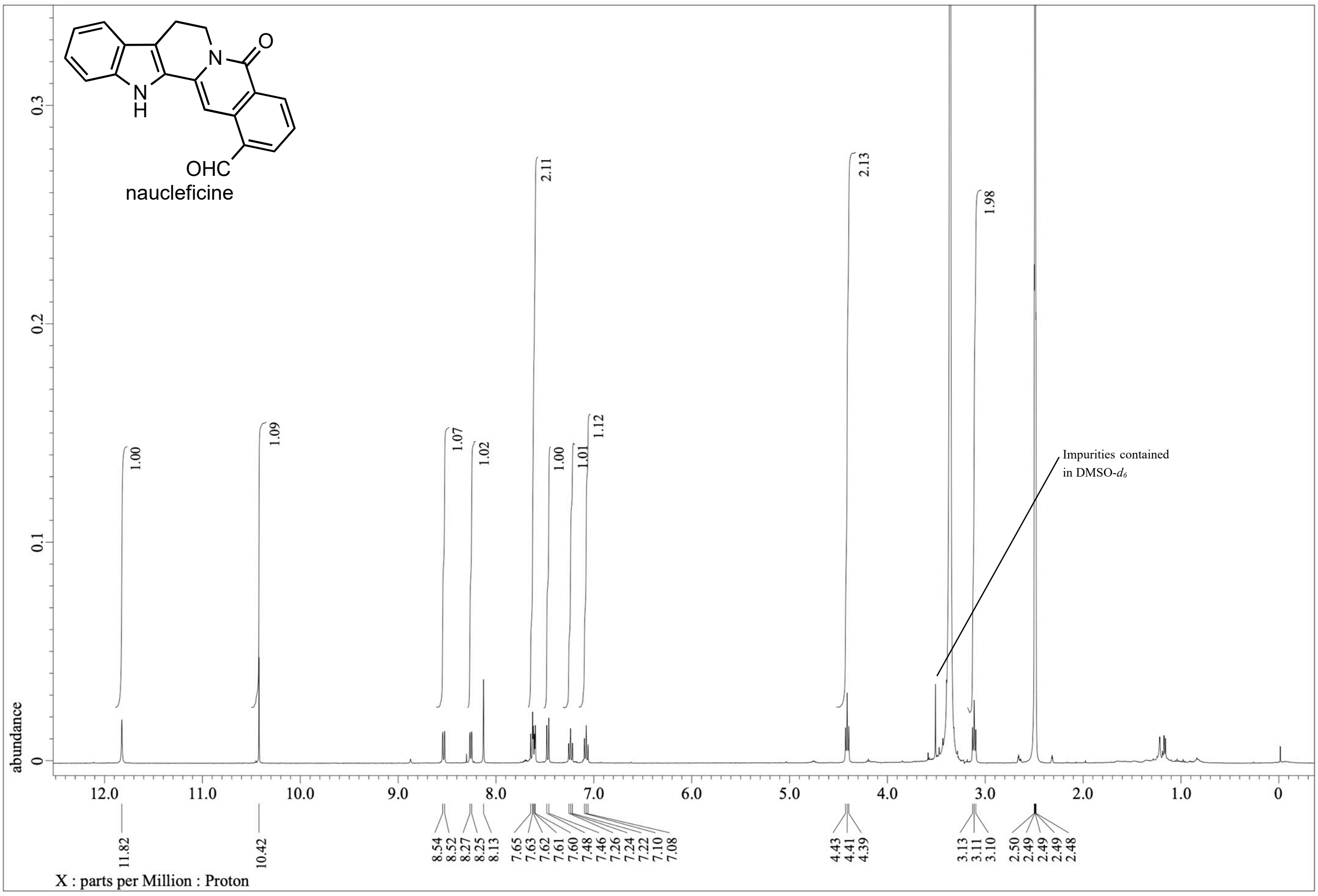

Naucleficine (**7**) ^{1}H-NMR ($CDCl_3$)

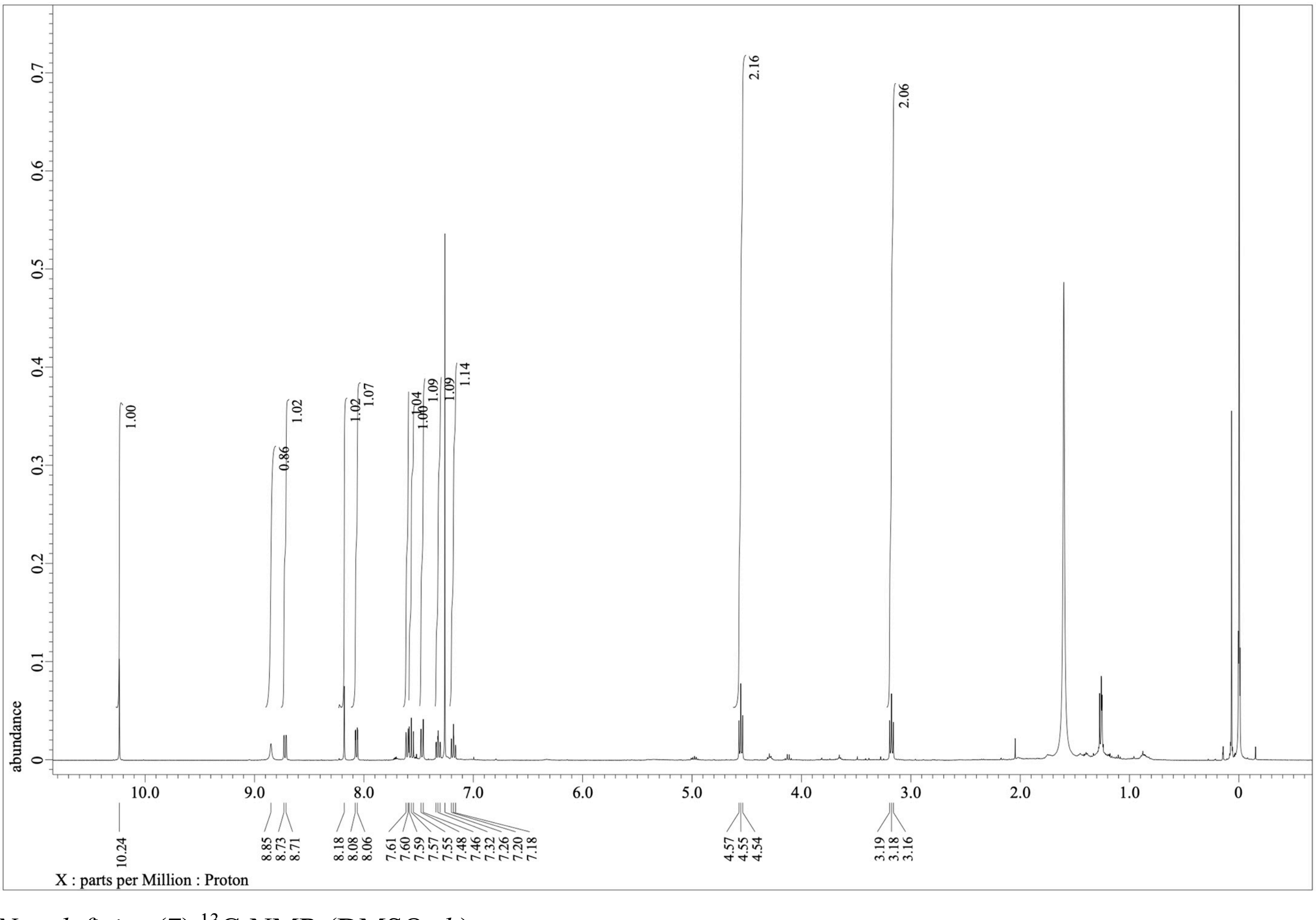

Naucleficine (**7**) ^{13}C-NMR (DMSO-d_6)

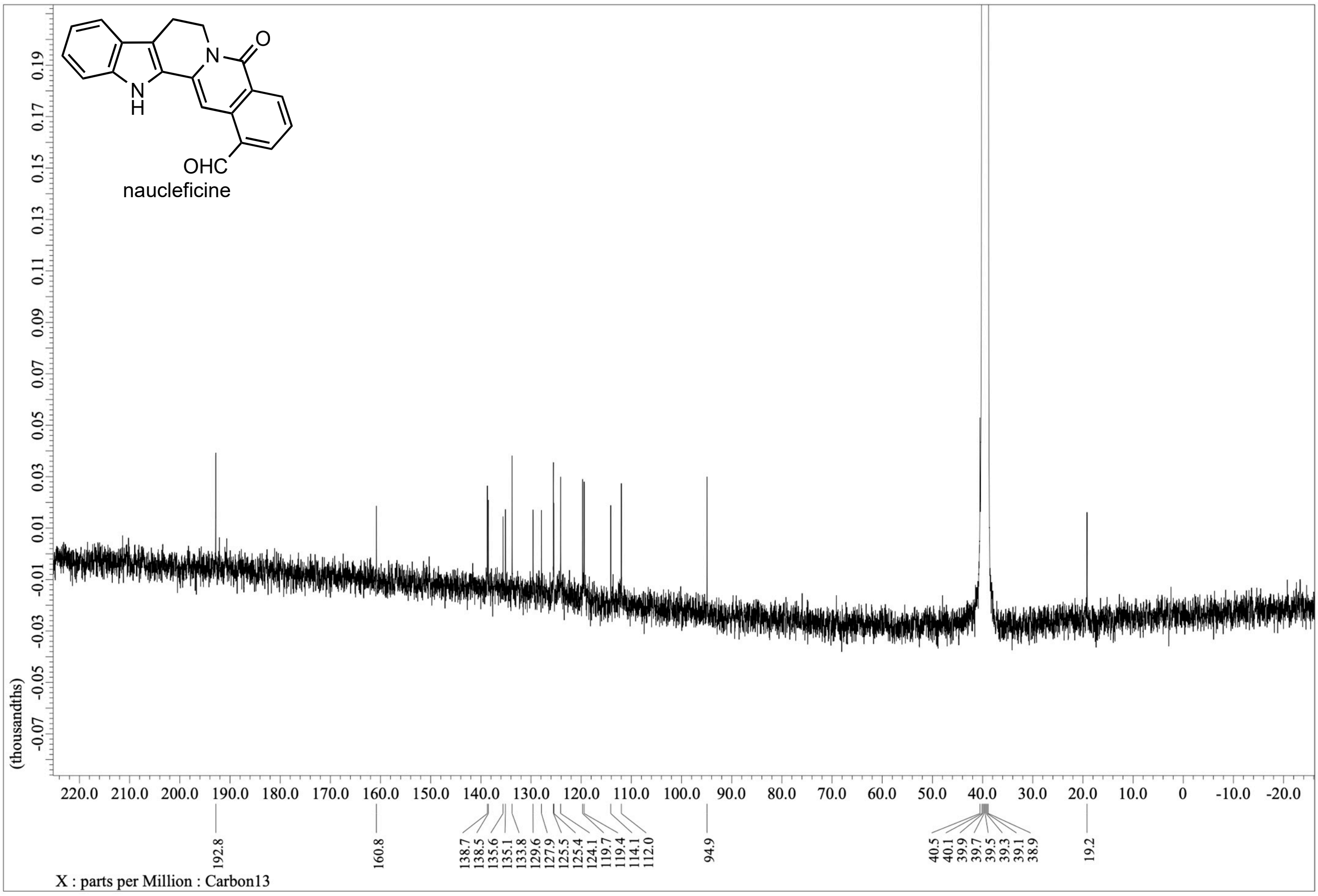

OHC
naucleficine
(thousandths)
X : parts per Million : Carbon13
192.8
160.8
138.7
138.5
135.6
135.1
133.8
129.6
127.9
125.5
125.4
124.1
119.7
119.4
114.1
112.0
94.9
40.5
40.1
39.9
39.7
39.5
39.3
39.1
38.9
19.2

Comparison of ^{1}H NMR and ^{13}C NMR spectroscopic data of Gao' synthetic

and our synthetic 22-hydroxyacuminatine.

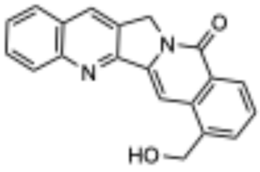

22-hydroxyacuminatine

Gao's Synthetic 22-hydroxyacuminatine		Our Synthetic 22-hydroxyacuminatine (**4**)	
^{1}H NMR (400 MHz, DMSO-d_6)	^{13}C NMR	^{1}H NMR (400 MHz, DMSO- d_6)	^{13}C NMR
4.95 (d, *J* = 5.4 Hz, 2H)	49.5	4.95 (d, *J* = 5.5 Hz, 2H)	49.6
5.38 (s, 2H)	61.1	5.36 (s, 2H)	61.2
5.51 (t, *J* = 5.4 Hz, 1H)	96.2	5.50 (t, *J* = 5.5 Hz, 1H)	96.2
7.58 (t, *J* = 7.7 Hz, 1H)	125.8	7.54–7.63 (m, 1H)	125.9 (2C)
7.69 (td, *J* = 7.9, 1.0 Hz, 1H)	125.9	7.69 (t, *J* = 7.8 Hz, 1H)	
7.75 (s, 1H)	126.7	7.73 (s, 1H)	126.7
7.81 (d, *J* = 6.9 Hz, 1H)	127.2	7.81 (d, *J* = 7.8 Hz, 1H)	127.3
7.86 (td, *J* = 7.7, 1.3 Hz, 1H)	127.8	7.83–7.87 (m, 1H)	127.9
8.12 (d, *J* = 7.9 Hz, 1H)	128.4	8.11 (d, *J* = 7.8 Hz, 1H)	128.5
8.21 (d, *J* = 8.5 Hz, 1H)	128.8	8.19 (d, *J* = 7.8 Hz, 1H)	128.9
8.31 (d, *J* = 8.2 Hz, 1H)	129.7	8.30 (d, *J* = 7.8 Hz, 1H)	129.7
8.67 (s, 1H)	130.2	8.66 (s, 1H)	130.2
	131.0		131.1
	131.2		131.3
	135.2		135.3
	138.4		138.5
	140.0		140.1
	148.0		148.0
	153.3		153.4
	159.9		159.9

Li, K.; Ou, J.; Gao, S. Total Synthesis of Camptothecin and Related Natural Products by a Flexible Strategy. *Angew. Chem., Int. Ed.* **2016**, *55*, 14778–14783.
